# Fast and accurate estimation of multidimensional site frequency spectra from low-coverage high-throughput sequencing data

**DOI:** 10.1093/gigascience/giac032

**Published:** 2022-05-17

**Authors:** Alex Mas-Sandoval, Nathaniel S Pope, Knud Nor Nielsen, Isin Altinkaya, Matteo Fumagalli, Thorfinn Sand Korneliussen

**Affiliations:** Department of Life Sciences, Silwood Park campus, Imperial College London, SL5 7PY, Ascot, UK; Department of Entomology, The Pennsylvania State University, 201 Old Main, University Park, PA 16802, USA; Department of Plant and Environmental Sciences, University of Copenhagen, Thorvaldsensvej 40, 1871 Frederiksberg C, Denmark; GLOBE, Section for Geogenetics, Øster Voldgade 5-7, 1350, Copenhagen, Denmark; Department of Life Sciences, Silwood Park campus, Imperial College London, SL5 7PY, Ascot, UK; School of Biological and Behavioural Sciences, Queen Mary University of London, London, UK; GLOBE, Section for Geogenetics, Øster Voldgade 5-7, 1350, Copenhagen, Denmark

**Keywords:** site frequency spectrum, high-throughput sequencing, genotype likelihoods, next-generation sequencing, maximum likelihood, population genetics, threading

## Abstract

**Background:**

The site frequency spectrum summarizes the distribution of allele frequencies throughout the genome, and it is widely used as a summary statistic to infer demographic parameters and to detect signals of natural selection. The use of high-throughput low-coverage DNA sequencing data can lead to biased estimates of the site frequency spectrum due to high levels of uncertainty in genotyping.

**Results:**

Here we design and implement a method to efficiently and accurately estimate the multidimensional joint site frequency spectrum for large numbers of haploid or diploid individuals across an arbitrary number of populations, using low-coverage sequencing data. The method maximizes a likelihood function that represents the probability of the sequencing data observed given a multidimensional site frequency spectrum using genotype likelihoods. Notably, it uses an advanced binning heuristic paired with an accelerated expectation-maximization algorithm for a fast and memory-efficient computation, and can generate both unfolded and folded spectra and bootstrapped replicates for haploid and diploid genomes. On the basis of extensive simulations, we show that the new method requires remarkably less storage and is faster than previous implementations whilst retaining the same accuracy. When applied to low-coverage sequencing data from the fungal pathogen *Neonectria neomacrospora*, results recapitulate the patterns of population differentiation generated using the original high-coverage data.

**Conclusion:**

The new implementation allows for accurate estimation of population genetic parameters from arbitrarily large, low-coverage datasets, thus facilitating cost-effective sequencing experiments in model and non-model organisms.

## Introduction

Over the past 2 decades, next-generation sequencing (NGS) technologies have allowed researchers to generate large amounts of genomic data for both model and non-model species [1]. Across various experimental settings, low-coverage whole-genome sequencing (lcWGS) is becoming one of the most popular approaches in population genomics studies [2], with short-read data being the most feasible option at the moment. At a fixed experimental budget, sequencing a larger sample size at the cost of decreasing the individual read depth has been the preferred strategy in population genetics because it is associated with less biased estimates of notable parameters [3]. However, under these conditions, the high degree of uncertainty that inherently exists for lcWGS data prevents the assignment of individual genotypes and single-nucleotide polymorphisms (SNPs) [4].

To solve this issue, statistical methods that compute a probability measure for each of the possible genotypes (the genotype likelihoods) and integrate over these probabilities in the downstream analyses have been proposed [5]. In general a genotype likelihood is calculated independently for each individual for each site and is the probability of the read data *D* given the true unobserved genotype *G*, which in a diploid context is given by

\begin{eqnarray*}
L(G=\lbrace A_1,A_2\rbrace \mid D) \propto \mathrm{\rm {Pr}}(D\mid G=\lbrace A_1,A_2\rbrace ),~ A_1,A_2 \in \lbrace A,C,G,T\rbrace
\end{eqnarray*}

Many genotype likelihood models exist [6–9], and the canonical genotype likelihood model is shown below; *M* denotes sequencing depth, *b_i_* is the nucleotide for the *i*th read, and *e_i_* is the associated error rate, which is in practice given by the phred-scaled base quality score of the nucleotides of the read:

\begin{eqnarray*}
\mathrm{Pr}(D\mid G=A_1A_2) & = & \prod\nolimits _{i=1}^M \mathrm{Pr}(b_i\mid G=A_1A_2) \\ && = \prod\nolimits _{i=1}^M\left[2^{-1} \mathrm{Pr}(b_i\mid A_1)+ 2^{-1} \mathrm{Pr}(b_i\mid A_2)\right], \\ \mathrm{Pr}(b_i \mid A) & = & \left\lbrace \begin{array}{@{}l@{\quad }l@{}}\frac{e_i}{3} & \mathrm{if~} b_i \ne A \\ 1-e_i & \mathrm{if~} b_i = A \end{array}\right.. \end{eqnarray*}

Previous studies have shown that summary statistics commonly used in population genetics can be reliably estimated from lcWGS data using genotype likelihoods [10–19]. The calculation of these estimators is implemented in the dedicated software packages ngsTools [20] and ANGSD [21]. Whilst being regarded as the gold standard tool kit for population genetic inferences from lcWGS data, these implementations tend to be computationally expensive and require a large file storage capacity when applied to large numbers of sequenced samples, limiting their scalability with modern experimental datasets.

The estimation of the site frequency spectrum (SFS) is one of the analyses most affected by poor scalability. The SFS is arguably one of the most important summary statistics of population genetic data because it summarizes the distribution of allele frequencies throughout the genome. The SFS contains invaluable information on the demographic and adaptive processes that shaped the evolution of the population under investigation [22]. For instance, an SFS showing an overrepresentation of rare alleles is an indication of an expanding population, while bottleneck events tend to deplete low-frequency variants. Complex scenarios of repeated bottlenecks and gene flow may also generate an excess of rare alleles [23, 24]. Similarly, a locus targeted by positive selection will exhibit an excess of rare variants, while balancing selection will cause an increase of common (i.e., intermediate-frequency) alleles.

The calculation of the joint, or multidimensional, SFS allows for the inference of the evolutionary relationships between populations [25]. In fact, many statistical methods to estimate demographic parameters from population genetic data use the multidimensional SFS as the sole input [26]. Additionally, widely used metrics of genetic differentiation between populations can be directly calculated from the multidimensional SFS, including estimators of the fixation index (*F*_ST_) and the population branch statistic (PBS) [27].

Here, we propose a method to efficiently estimate the multidimensional SFS (and statistics thereof) for an arbitrary number of populations of either haploids or diploids, given lcWGS data. We evaluate its performance over a range of experimental scenarios and describe its new features in terms of speed and data storage. This novel implementation greatly reduces the computational cost and storage requirements through an accelerated expectation maximization (EM) algorithm that uses a subset of sample allele frequency likelihoods for any given SNP and allows for the calculation of *F*_ST_ and PBS values on the fly. As an illustration, we demonstrate the applicability of this tool by calculating metrics of genetic differentiation between strains of haploid fungus *Neonectria neomacrospora* from NGS data. This novel method is part of the ANGSD pipeline [21, 28].

## Materials and Methods

### Fast calculation of site frequency likelihoods

We seek to compute likelihoods *y* of possible sample allele frequencies for a single site, given a set of genotype likelihoods across samples. For a sample of diploids with *n* individuals, *y* is a 2*n* + 1 vector containing the likelihood of observing zero derived alleles, 1 derived allele, etc., up to 2*n* derived alleles. It follows that first and last elements of this vector represent monomorphic alleles. Each element of *y* is a very large combinatorial product and sum, even for a moderate number of individuals *n*. A dynamic programming algorithm described by Fumagalli et al. [5] and implemented by Korneliussen et al. [21] computes the entire vector efficiently in $\mathcal {O} (n^2)$. Assuming that the likelihood vector is unimodal (which is frequently the case and easy to verify on the fly), Han et al. [19] proposed an algorithm that only updates entries around the mode, reducing cost to $\mathcal {O} (n)$. We have implemented this low-cost version of the original algorithm in ANGSD. We here emphasize that the novelty lies not in the development of the dynamic programming algorithm presented by Han et al. [19] but in the extension of this to a haploid and multidimensional population context. To our knowledge, there is no other readily available implementation.

We have also developed an analogous algorithm for haploids, in which case *y* has *n* + 1 elements and *y*[*i*] is the likelihood of *i* derived alleles in a sample of *n* haploids. The quantity *y* is initialized using the genotype likelihoods for the ancestral and derived states in the first haploid sample ($x^{(1)}_{0}$ and $x^{(1)}_1$, respectively) so that $y^{(1)} = [x^{(1)}_{0}, x^{(1)}_1]$, and then is incrementally updated with genotype likelihoods from subsequent samples: at the *i*th iteration, given the output *y*^(*i* − 1)^ from the previous iteration and the genotype likelihoods $x^{(i)}_{0},x^{(i)}_{1}$ for the *i*th sample, the *j*th element of the updated likelihood vector is equal to,

(1) \begin{eqnarray*}
\phi (i,j) = y^{(i)}[j] = \left\lbrace \begin{array}{@{}l@{\quad }l@{}}x^{(i)}_0 y^{(i-1)}[0] & \text{if } j = 0 \\ \\ x^{(i)}_1 y^{(i-1)}[i-1] & \text{if } j = i \\ \\ \frac{(i-j)}{i}x^{(i)}_0 y^{(i-1)}[j]+ \frac{j}{i} x^{(i)}_1 y^{(i-1)}[j-1]& \text{otherwise,} \\ \end{array}\right. \end{eqnarray*}

so that the length of *y* increases by 1 with each iteration (e.g., the superscript (*i*) indicates that the vector incorporates genotype likelihoods up to the *i*th sample and thus has *i* + 1 elements). As for the diploid case, the full recursion (on *n* haploids) can be performed in $\mathcal {O}(n)$ by only updating the *y* in a band of allele frequencies wherein the likelihoods exceed some predefined threshold ϵ (Algorithm 1). In the rare cases where the site frequency likelihoods are not unimodal, we revert to the original $\mathcal {O}(n^2)$ algorithm. The derivation for equation (1) is in the Supplementary Information section 1.

**Figure:**
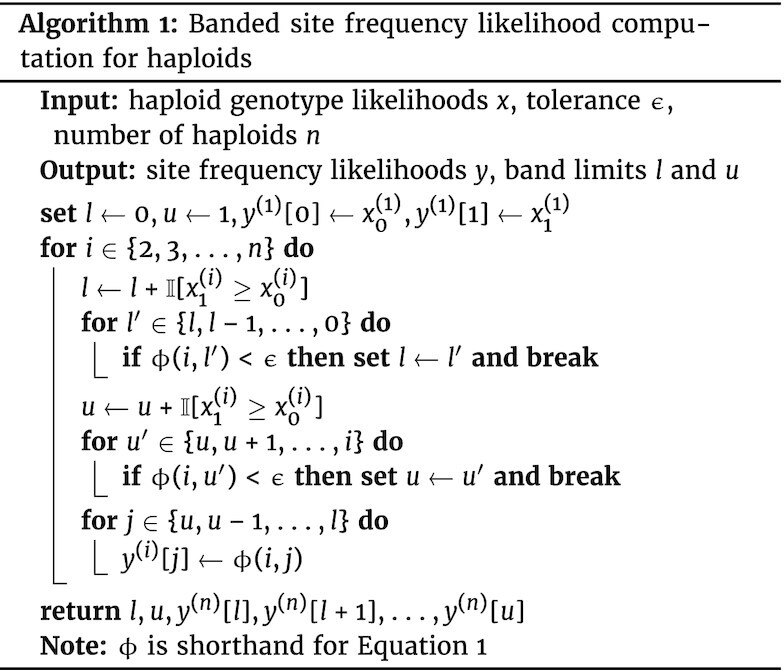


### Applications of site frequency likelihoods

Given vectors of site frequency likelihoods for sites across an arbitrarily large genomic region such as a segment of a chromosome, we can obtain a maximum likelihood estimate of the associated SFS (or its multidimensional analogue for multiple populations) via EM [21]. Many statistics of interest are either linear combinations of elements of the SFS (e.g., various estimators of the population-scaled mutation rate θ) or are ratios involving such linear combinations (e.g., *F*_ST_). In many cases, we are interested in the local behaviour of these statistics within an interval around a locus of interest.

However, these local estimates may involve few segregating sites and thus may be particularly sensitive to low-coverage data and/or sequencing errors. We can reduce the variance in these local estimates by leveraging genome-wide information and using the globally estimated SFS as the prior in an empirical Bayes procedure [12]. Specifically, let $l^{(s)}_k,u^{(s)}_k$, and $y^{(s)}_k$ be the lower bound, upper bound, and likelihood band, respectively, for the sample allele frequency at site *s* in population *k*, as output by Algorithm 1 or its diploid variant. Let Θ(*i*_1_, …, *i_P_*) be a linear statistic of allele frequencies *i* across *P* populations, and *z* be the *P*-dimensional global SFS. The empirical Bayes estimate of Θ across an arbitrarily small interval $\mathcal {M}$ is as follows:

\begin{eqnarray*}
\hat{\Theta }_{\mathrm{ EB}} & = & \sum\nolimits _{s \in \mathcal {M}} C_s^{-1} \sum\nolimits _{i_1=l_1^{(s)}}^{u_1^{(s)}} \dots \sum\nolimits _{i_P=l_P^{(s)}}^{u_P^{(s)}} \Theta (i_1,\dots ,i_P) z[i_1,\dots ,i_P] \prod\nolimits _{k=1}^P y_k^{(s)}[i_k] \\ && \quad\quad C_s^{-1} = \sum\nolimits _{i=l_1^{(s)}}^{u_1^{(s)}} \dots \sum\nolimits _{i=l_P^{(s)}}^{u_P^{(s)}} z[i_1,\dots ,i_P] \prod\nolimits _{k=1}^P y_k^{(s)}[i_k]
\end{eqnarray*}

For many organisms, the polarization of alleles into ancestral and derived states is not possible owing to lack of ancestral genomic material or a recently diverged outgroup. In this case, it is preferable to fold the SFS such that the frequency of the minor allele is estimated instead. To this end, we generalized the single-population probability model for the folded SFS in [5] to an arbitrary number of populations and derived an EM update for efficient optimization (Algorithm 2). Briefly, this is accomplished by introducing per-site latent variables that indicate the number of non-ancestral alleles in the sample and whether the site is correctly polarized, then taking the expectation of the joint log probability function with regard to these latent variables to find the EM update [21,29] (further details are in the Supplementary Information). Local statistics that are symmetric with regard to allele polarization may then be estimated using the (global) folded SFS and the empirical Bayes procedure described above.

**Figure:**
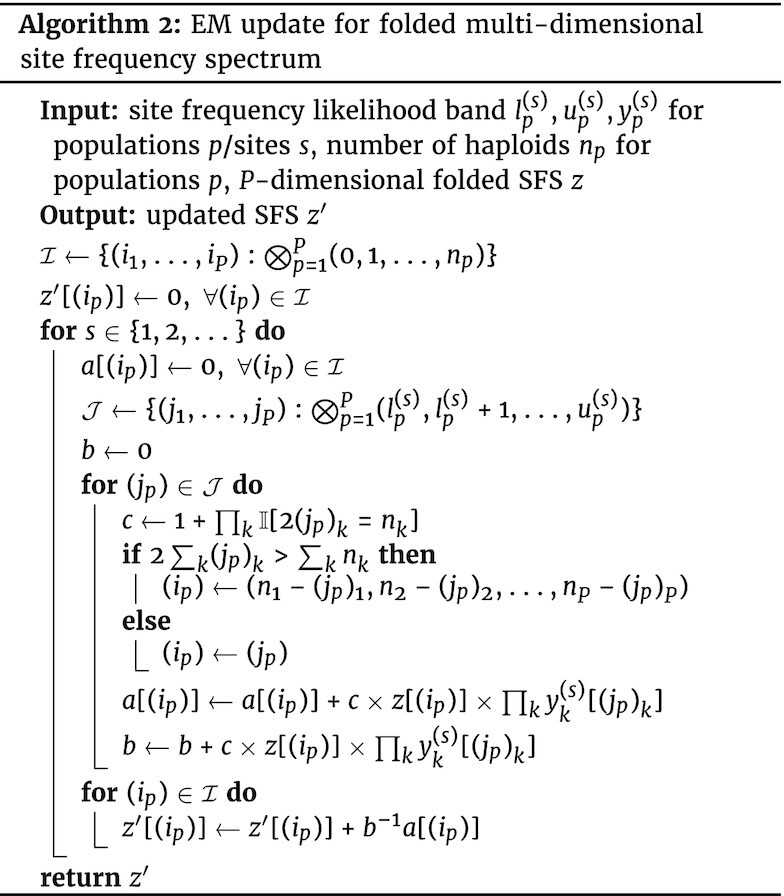


### Benchmarking

To compare the computational performance of the new implementation with the existing method [21], we examined the elapsed real time of the site allele frequency likelihood calculation (“-doSaf”), disk usage of BGZF compressed site allele frequency likelihood files (“saf.gz”), maximum memory usage, and the elapsed real time of the maximum likelihood estimation of the SFS (“realSFS”). We performed these analyses on NGS data for 5, 10, 25, 50, 100, 150, and 200 samples from the 1000 Genomes Project Phase 3 dataset [30] using Chromosome 1. The dataset consists of 14 individuals from Southern Han Chinese (CHS) group, 99 individuals from Finnish in Finland (FIN) group, and 87 individuals from British in England and Scotland (GBR) group [30]. We emphasize that we are subsampling across all 200 individuals assuming that they are from a single population: the purpose of this specific benchmark is to assess the computational performance rather than the accuracy of SFS estimation.

We used 5 replicates for each step in the analyses and retained the lowest value for elapsed real times, to minimize the influence of concurrent processes on our multiuser system. All analyses were conducted on a Red Hat Enterprise Linux Server 7.7 (Maipo) with Intel(R) Xeon(R) Gold 6152 CPUs at 2.10 GHz (x86_64) for benchmarking purposes. The commands used are angsd -b ${\$}${FILE} -anc ancestral.fq -doSaf 1 -gl 1 -r 1 -out ${\$}${FILE} and realSFS ${\$}${FILE}.saf.idx.

### Accuracy on simulated data

To test the accuracy of the new implementation for the estimation of the multidimensional SFS, we simulated 60 pseudo-chromosomes for 3 equally sampled populations under a realistic demographic model of recent human history [26] using the software ms [31]. Simulated data consisted of sequences that were 1/10 of the length of human chromosome 22 (≈5 Mb) with realistic values of mutation and recombination rates. The command line used was “ms 60 1 -t 1935 -r 2167 5130456 -I 3 20 20 20 -n 1 1.682020 -n 2 3.736830 -n 3 7.292050 -eg 0 2 116.010723 -eg 0 3 160.246047 -ma x 0.881098 0.561966 0.881098 x 2.797460 0.561966 2.797460 x -ej 0.028985 3 2 -en 0.028985 2 0.287184 -ema 0.028985 3 x 7.293140 x 7.293140 x x x x x -ej 0.197963 2 1 -en 0.303501 1 1” with seed numbers “44349 37512 34833.” The “ms” command produced 1 replicate of 60 sampled haplotypes (ms 60 1) for 3 populations with equal sample size (-I 3 20 20 20) with fixed mutation and recombination rates (-t 1935 -r 2167) scaled by the region length (5,130,456 bp). These values correspond to realistic average values of mutation [32] and recombination rates [33] in the human genome. The 3 populations experienced changes in effective sizes (switches -n, -eg, and -en), gene flow (-ma and -ema), and splits (-ej) following a previously proposed demographic model [26]. Simulations can be run using “msprime” [34] with the program “mspms,” which allows “ms” commands to be replaced. The simulation generated data for 12,335 diallelic SNPs, which were then converted into genotype likelihoods using the utility program called msToGlf found in the ANGSD software suite [21] (for details regarding the simulation algorithm we refer to [12]). We choose an unrealistic high error rate of $1\%$ to show the performance of our method in a worst-case setting [35]. From these simulated genomes and SNPs, we generated 100 distinct replicates of genotype likelihood data for each tested scenario of average per-site read depth (1×, 2×, 10×, and 20×) and considered only variables sites for ease of computation. For each replicate, we estimated SFS using ANGSD following the aforementioned new implementation and compared the results at different depths and against the ground truth. We assessed performance by calculating the root mean squared deviation (RMSD) and standardized bias (SB), the latter being the difference of estimated and true values divided by the true value.

### Application to real data

We analysed whole-genome sequencing data from the haploid fungal pathogen *N. neomacrospora* [36]. We analysed 70 samples for 3 sampling areas, corresponding to British Columbia (BC, 6 samples), Quebec (QC, 15 samples), and Europe (EU, 49 samples). We filtered out reads with mapping quality <30 and nucleotides with a base quality score <20 (in Phred scale). We used ANGSD to estimate the multidimensional SFS for use as prior information in the local estimation of *F*_ST_ [37] and PBS [27] in overlapping sliding windows of 20 kb with a step of 2 kb. To assess the accuracy at lower sequencing depth, we repeated the analyses on a randomly downsampled dataset where we retained only 25% of sequenced reads.

## Results

### Computational performance

We first compared the computational performance between the original and new (labeled “banded”) implementation for estimating the site frequency spectrum at different sample sizes. We observe an almost linear increase of runtime and memory usage with the number of samples using the original implementation (Table 1). On the other hand, we observe a lower disk and memory usage and runtime for large sample sizes using the new banded implementation, which no longer exhibits a proportional increase of memory with sample size.

**Table 1.** Benchmarking

		doSaf		realSFS	
Sample size	Version	Time (min)	File size (GB)	Time (min)	Memory usage (GB)
5	Original	76	7.3	5	15.3
	Banded	79	2.2	7	10.5
10	Original	135	16.8	13	21.7
	Banded	122	3.9	11	12.0
25	Original	279	39.2	81	47.1
	Banded	238	5.6	66	12.7
50	Original	547	64.3	123	85.3
	Banded	421	6.5	88	13.4
100	Original	1,292	105.9	283	164.7
	Banded	965	7.2	126	14.1
150	Original	2,055	142.3	315	244.2
	Banded	1,342	8.0	156	15.1
200	Original	2,991	162.0	492	323.7
	Banded	2,022	8.0	178	15.0

Benchmarking of original and the novel banded implementation of the SFS estimation using data from Chromosome 1 of the individuals randomly selected from 1000 Genomes Project Phase 3 Dataset [30]. realSFS time: minimum elapsed time among 5 replicates; memory usage: maximum value of maximum memory usage among 5 replicates.

### Estimation of site frequency spectra

We estimated multidimensional SFS from simulated sequencing data and compared results across different sequencing depths (Figs 1 and2 and Supplementary Figs S1–S28). For the interpretation of these figures, the high-depth scenarios can be assumed to be the true SFS.

**Figure 1:**
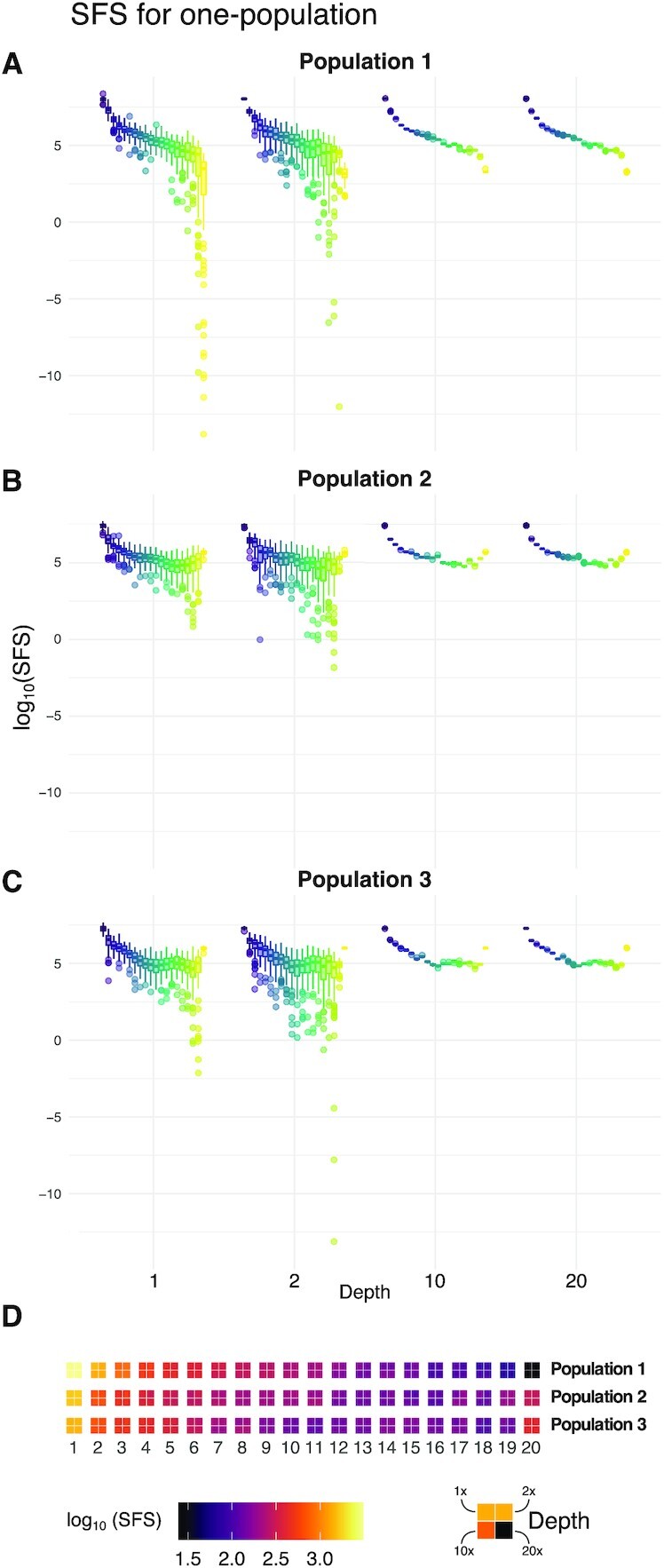
: Estimation of 1D SFS. Distribution of estimated SFS for individual populations at 4 different sequencing average depths (1×, 2×, 10×, and 20×) across 100 simulations. Each box plot represents the distribution of the estimated number of alleles with a certain derived allele frequency, in log_10_ scale, across the 100 simulated data replicates. **D** shows a 4-tile plot of the mean values of the distributions, where each tile corresponds to a different depth. Notice that we do not observe any difference in any 4-tiles across any population or sequencing depth. The monomorphic positions are omitted in all the panels.

**Figure 2:**
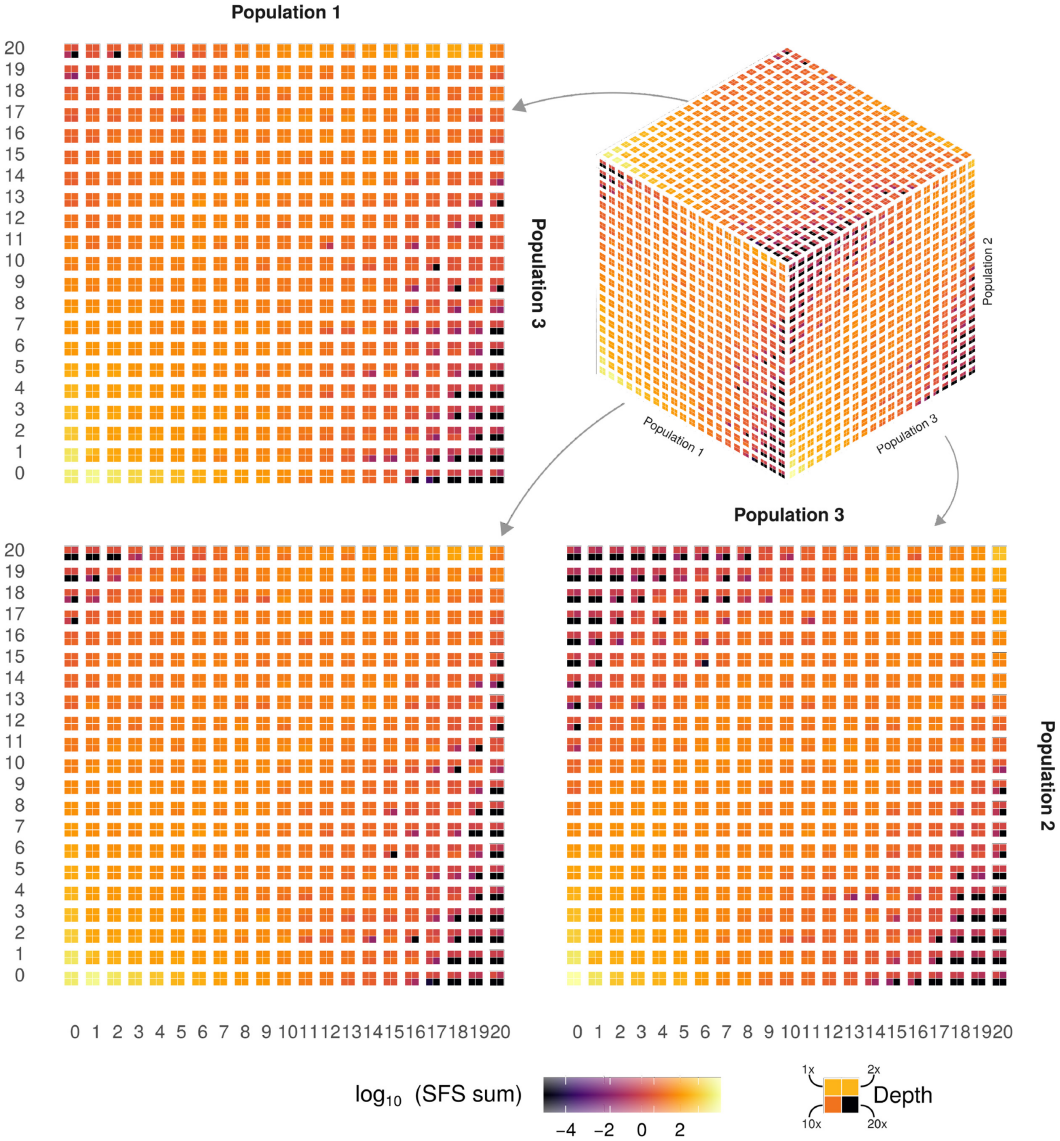
: Estimation of 3D SFS. Joint distribution of estimated SFS for 3 populations in 4-tile plot, where each tile corresponds to a different depth. SFS is represented in 3 dimensions (top right panel) and as marginal 2D SFS (top left and bottom panels), where the third axis is marginalized by its sum value. Values are reported in in log_10_ scale.

We observe that the mean estimates of SFS across replicates do not differ between sequencing depths except for a few private and low-frequency bins, where lower depths tend to overestimate. Estimates from low depths (1× and 2×) present a larger variance in their distributions and, therefore, show higher RMSD than estimates from high depths (10× and 20×) (Figs 1, 2 and Supplementary Figs S1, S4). This pattern is observed in 1D, 2D, and 3D SFS (Supplementary Figs S7 and S9).

Within each SFS, low frequencies exhibit high RMSD (Supplementary Figs S1, S4), while high frequencies have higher absolute values of SB (Supplementary Figs S2, S5) as a result of having low counts. In 2D and 3D SFS, most of the errors are concentrated in population-private (high absolute SB) or low-frequency bins (high RMSD) (Supplementary Figs S7–S10). We replicated all these findings for both unfolded and folded multidimensional spectra (Supplementary Figs S14–S28).

Finally, the choice of tolerance for calculation of site frequency likelihood bands had a minimal impact on the estimated SFS, in both folded and unfolded cases (Table 2), as previously suggested [19].

**Table 2.** Effect of tolerance values on estimated SFS

SFS	Depth	Tolerance	Mean KL
Unfolded	2	1e−4	3.51e−08
		1e−6	3.79e−12
		1e−8	1.035e−15
	5	1e−4	4.69e−08
		1e−6	4.75e−13
		1e−8	3.78e−16
	10	1e−4	6.32e−10
		1e−6	6.92e−14
		1e−8	3.011e−16
Folded	2	1e−4	1.67e−08
		1e−6	2.011e−12
		1e−8	3.63e−16
	5	1e−4	2.69e−09
		1e−6	2.88e−13
		1e−8	1.15e−16
	10	1e−4	6.43e−10
		1e−6	6.67e−14
		1e−8	1.38e−16

For each tested scenario, we calculated the average KL divergence over 100 repetitions between the 2D-SFS with tolerance equal to 0 and several alternative values.

### Population differentiation in *Neonectria neomacrospora*

We used the method described herein to estimate the SFS from whole-genome sequences of the fungal pathogen *N. neomacrospora*. We analyzed 70 samples for 3 main sampling areas (BC, QC, EU) [36] and downsampled the original sequencing data to mimic the challenges associated with low-coverage settings. From the estimated SFS, we sought to estimate the levels of genetic differentiation, as measured by PBS [27] in sliding windows.

**Figure 3:**
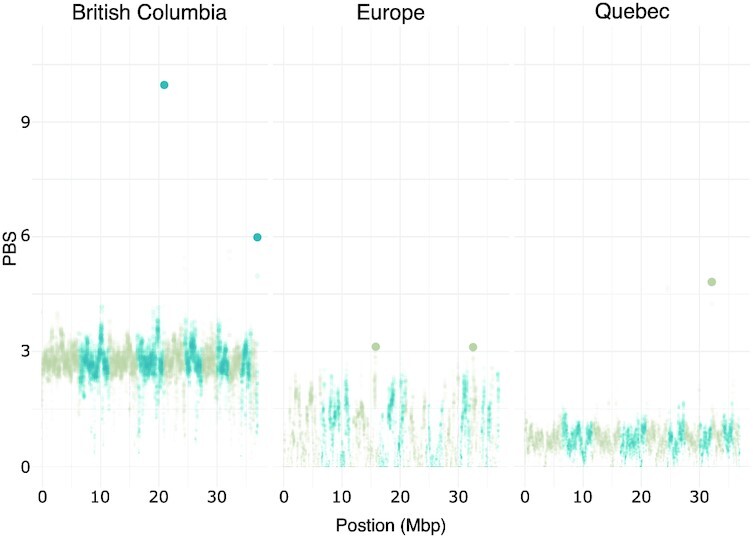
: Sliding windows scan for genetic differentiation in 3 populations of *Neonectria neomacrospora*. We calculated PBS in sliding windows of 20 kb with a step of 2 kb. Each panel represents a separate scan where each population was considered the target and the remaining 2 controls. The highlighted points indicate windows with an empirical rank *P* < 10^−3^ in each population. Sequencing data were randomly downsampled to 25% of their original amount.

Results show that when BC is the target population in PBS calculation, we observe greater levels of differentiation (Fig. 3) than those obtained when QC or EU are considered target populations, in line with recent findings [36]. We also highlighted outlier windows with exceptionally high values of PBS compared to the empirical distribution (Fig. 3). Notably, we obtained similar results when using the full-coverage sequencing data (Supplementary Fig. S29), although the scale of PBS values differs.

## Conclusion

In this study, we present a novel implementation for the estimation of multidimensional SFS from lcWGS data. We show that the new method is faster and requires less memory and data storage than the currently available solution. Notably, these improvements do not come at the cost of accuracy because estimated spectra display low error rates on simulated low-depth data or artificially downsampled real sequencing data.

We foresee several avenues for improving the methods and software developed for this study. For instance, additional metrics of genetic differentiation can be easily extracted from the estimated multidimensional SFS, such as genome divergence *D*_XY_ [38]. Likewise, extending this framework to an arbitrary ploidy would allow the estimation of SFS for polyploid species. Finally, a future user-friendly GUI for the ANGSD pipeline would make these analyses accessible to a broader user base.

The estimation of fundamental population genetic parameters over genomic intervals is crucial for elucidating how various evolutionary forces have acted to shape contemporary genetic polymorphism across species and populations. The development and implementation of sound statistical and bioinformatic methods that are robust to the uncertainty inherent in low-coverage sequence data and that leverage genome-wide information to improve local estimates are necessary for maintaining cost-effectiveness without sacrificing accuracy in the generation of large-scale population genomic data.

## Implementation Details

The program is implemented in a fast multi-threaded C++ program and takes as input either BAM/CRAM files or BCF/VCF files containing genotype likelihood files as produced from standard tools such as GATK [7] or SAMtools [6].

## Availability of Source Code and Requirements

- Project name: ANGSD/realSFS
- Project home page: https://github.com/ANGSD, https://www.popgen.dk/angsd
- Operating systems: Platform independent
- Programming language: C/C++
- Other requirements: htslib
- License: GPL
- RRID:SCR_021865
- biotools: angsd

## Data Availability

Supporting data and an archival copy of the code are available via the *GigaScience* repository, GigaDB [39].

## Additional Files

**Supplementary Figure S1**: Root mean squared deviation of population-based SFS values shown in Fig. 1. Each panel represents the root mean squared deviation of the SFS for a different population. Each of the coloured distributions of the box plot shows the root mean squared deviation values for the log_10_ of the number of occurrences of each derived allele frequency.

**Supplementary Figure S2**: Standardized bias of population-based SFS values shown in Fig. 1. Each panel represents the standardized bias of the SFS for a different population, at different depths (1×, 2×, 10×, and 20×). Each of the coloured distributions of the box plot shows the values for the standardized bias of the log_10_ of the number of occurrences of each derived allele frequency.

**Supplementary Figure S3**: Population-based SFS. Each panel represents the SFS for a different population, for each derived allele frequency. Each of the coloured distributions of the box plot shows the log_10_ of the number of occurrences in depths 1×, 2×, 10×, and 20×.

**Supplementary Figure S4**: Root mean squared deviation of population-based SFS values shown in Supplementary Fig. S3. Each panel represents the root mean squared deviation of the SFS for a different population, for each derived allele frequency. Each of the coloured distributions of the box plot shows root mean squared deviation of the log_10_ of the number of occurrences in depths 1×, 2×, 10×, and 20×.

**Supplementary Figure S5**: Standardized bias of population-based SFS values shown in Supplementary Fig. S3. Each panel represents the standardized bias of the SFS for a different population, for each derived allele frequency. Each of the coloured distributions of the box plot shows the standardized bias of the log_10_ of the number of occurrences in depths 1×, 2×, 10×, and 20×.

**Supplementary Figure S6**: Two-population joint SFS. Each panel represents the 2-population joint SFS for a different pair of populations, for each derived allele frequency. Each colour of the 4-tile squares is according to the log_10_ of the number of occurrences at depths 1×, 2×, 10×, and 20×, like in Fig. 2.

**Supplementary Figure S7**: Root mean squared deviation of the 2-population joint SFS values shown in Supplementary Fig. S6. Each panel represents the root mean squared deviation of the 2-population joint SFS for a different pair of populations, for each derived allele frequency. Each colour of the 4-tile squares is according to the root mean squared deviation of the log_10_ of the number of occurrences at depths 1×, 2×, 10×, and 20×, like in Fig. 2.

**Supplementary Figure S8**: Standardized bias of the 2-population joint SFS values shown in Supplementary Fig. S6. Each panel represents the standardized bias of the 2-population joint SFS for a different pair of populations, for each derived allele frequency. Each colour of the 4-tile squares is according to the standardized bias of the log_10_ of the number of occurrences at depths 1×, 2×, 10×, and 20×, like in Fig. 2.

**Supplementary Figure S9**: Root mean squared deviation of the 3-population joint SFS shown at Fig. 2. Each panel represents the root mean squared deviation of the 3-population joint SFS for a different pair of populations, for each derived allele frequency. Each colour of the 4-tile squares is according to the root mean squared deviation of the log_10_ of the marginal sum of the number of occurrences across the third axis at depths 1×, 2×, 10×, and 20×, like in Fig. 2.

**Supplementary Figure S10**: Standardized bias of 3-population joint SFS shown at Fig. 2. Each panel represents the standardized bias of the 3-population joint SFS for a different pair of populations, for each derived allele frequency. Each colour of the 4-tile squares is according to the standardized bias of the log_10_ of the marginal sum of the number of occurrences across the third axis at depths 1×, 2×, 10×, and 20×, like in Fig. 2.

**Supplementary Figure S11**: Three-population joint SFS. Each panel represents the 3-population joint SFS for a different pair of populations, for each derived allele frequency. Each colour of the 4-tile squares is according to the log_10_ of the mean of the number of occurrences across the third axis at depths 1×, 2×, 10×, and 20×, like in Fig. 2.

**Supplementary Figure S12**: Root mean squared deviation of the 3-population joint SFS shown in Supplementary Fig. S11. Each panel represents the root mean squared deviation of the 3-population joint SFS for a different pair of populations, for each derived allele frequency. Each colour of the 4-tile squares is according to the root mean squared deviation of the log_10_ of the mean of the number of occurrences across the third axis at depths 1×, 2×, 10×, and 20×, like in Fig. 2.

**Supplementary Figure S13**: Standardized bias of 3-population joint SFS shown in Supplementary Fig. S11. Each panel represents the standardized bias of the 3-population joint SFS for a different pair of populations, for each derived allele frequency. Each colour of the 4-tile squares is according to the standardized bias of the log_10_ of the mean of the number of occurrences across the third axis at depths 1×, 2×, 10×, and 20×, like in Fig. 2.

**Supplementary Figure S14**: Population-based folded SFS. Each panel represents the folded SFS for a different population, for depths 1×, 2×, 10×, and 20×. Each of the coloured distributions of the box plot shows the values for the log_10_ of the number of each allele frequency.

**Supplementary Figure S15**: Root mean squared deviation of the population-based folded SFS. Each panel represents the root mean squared deviation of the folded SFS for a different population. Each of the coloured distributions of the box plot shows the values for root mean squared deviation of the log_10_ of the number of each allele frequency.

**Supplementary Figure S16**: Standardized bias of the population-based folded SFS. Each panel represents the standardized bias of the folded SFS for a different population. Each of the coloured distributions of the box plot shows the values for the standardized bias of the log_10_ of the number of each allele frequency.

**Supplementary Figure S17**: Popualtion-based folded SFS. Each panel represents the folded SFS for a different population, for each allele frequency. Each of the coloured distributions of the box plot shows the log_10_ of the number of occurrences in depths 1×, 2×, 10×, and 20×.

**Supplementary Figure S18**: Root mean squared deviation of population-based folded SFS values shown in Supplementary Fig. S17. Each panel represents the standardized bias of the folded SFS for a different population, for each allele frequency. Each of the coloured distributions of the box plot shows the standardized bias of the log_10_ of the number of occurrences in depths 1×, 2×, 10×, and 20×.

**Supplementary Figure S19**: Standardized bias of population-based folded SFS values shown in Supplementary Fig. S17. Each panel represents the standardized bias of the folded SFS for a different population, for each allele frequency. Each of the coloured distributions of the box plot shows the standardized bias of the log_10_ of the number of occurrences in depths 1×, 2×, 10×, and 20×.

**Supplementary Figure S20**: Two-population joint folded SFS. Each panel represents the 2-population joint folded SFS for a different pair of populations, for each allele frequency. Each colour of the 4-tile squares is according to the log_10_ of the number of occurrences at depths 1×, 2×, 10×, and 20×, like in Fig. 2.

**Supplementary Figure S21**: Root mean squared deviation of the 2-population joint folded SFS values shown at Supplementary Fig. S20. Each panel represents the root mean squared deviation of the 2-population joint SFS for a different pair of populations, for each allele frequency. Each colour of the 4-tile squares is according to the root mean squared deviation of the log_10_ of the number of occurrences at depths 1×, 2×, 10×, and 20×, like in Fig. 2.

**Supplementary Figure S22**: Standardized bias of the 2-population joint folded SFS values shown in Supplementary Fig. S20. Each panel represents the standardized bias of the 2-population joint SFS for a different pair of populations, for each allele frequency. Each colour of the 4-tile squares is according to the standardized bias of the log_10_ of the number of occurrences at depths 1×, 2×, 10×, and 20×, like in Fig. 2.

**Supplementary Figure S23**: Three-population joint folded SFS. Each panel represents the 3-population joint folded SFS for a different pair of populations, for each allele frequency. Each colour of the 4-tile squares is according to the log_10_ of the marginal sum of the number of occurrences across the third axis at depths 1×, 2×, 10×, and 20×, like in Fig. 2.

**Supplementary Figure S24**: Root mean squared deviation of the 3-population joint folded SFS shown in Supplementary Fig. S23. Each panel represents the root mean squared deviation of the 3-population joint SFS for a different pair of populations, for each allele frequency. Each colour of the 4-tile squares is according to the root mean squared deviation of the log_10_ of the marginal sum of the number of occurrences across the third axis at depths 1×, 2×, 10×, and 20×, like in Fig. 2.

**Supplementary Figure S25**: Standardized bias of 3-population joint folded SFS shown in Supplementary Fig. S20. Each panel represents the standardized bias of the 3-population joint SFS for a different pair of populations, for each derived allele frequency. Each colour of the 4-tile squares is according to the standardized bias of the log_10_ of the marginal sum of the number of occurrences across the third axis at depths 1×, 2×, 10×, and 20×, like in Fig. 2.

**Supplementary Figure S26**: Three-population joint SFS. Each panel represents the 3-population joint folded SFS for a different pair of populations, for each derived allele frequency. Each colour of the 4-tile squares is according to the log_10_ of the mean of the number of occurrences across the third axis at depths 1×, 2×, 10×, and 20×, like in Fig. 2.

**Supplementary Figure S27**: Root mean squared deviation of the 3-population joint SFS shown in Supplementary Fig. S11. Each panel represents the root mean squared deviation of the 3-population joint SFS for a different pair of populations, for each derived allele frequency. Each colour of the 4-tile squares is according to the root mean squared deviation of the log_10_ of the mean of the number of occurrences across the third axis at depths 1×, 2×, 10×, and 20×, like in Fig. 2.

**Supplementary Figure S28**: Standardized bias of 3-population joint SFS shown in Supplementary Fig. S11. Each panel represents the standardized bias of the 3-population joint SFS for a different pair of populations, for each derived allele frequency. Each colour of the 4-tile squares is according to the standardized bias of the *l*og_10_ of the mean of the number of occurrences across the third axis at depths 1×, 2×, 10×, and 20×, like in Fig. 2.

**Supplementary Figure S29**: Sliding windows scan for genetic differentiation in 3 populations of *Neonectria neomacrospora*. We calculated PBS in sliding windows of 20 kb with a step of 2 kb. Each panel represents a separate scan where each population was considered the target and the remaining 2 controls. The highlighted points indicate windows with an empirical rank *P* < 10^−3^ in each population. Compared to Fig. 3, sequencing data are not downsampled.

giac032_GIGA-D-21-00341_Original_Submission

giac032_GIGA-D-21-00341_Revision_1

giac032_GIGA-D-21-00341_Revision_2

giac032_GIGA-D-21-00341_Revision_3

giac032_Response_to_Reviewer_Comments_Original_Submission

giac032_Response_to_Reviewer_Comments_Revision_1

giac032_Response_to_Reviewer_Comments_Revision_2

giac032_Reviewer_1_Report_Original_Submission Bjarki Eldon, PhD -- 12/3/2021 Reviewed

giac032_Reviewer_1_Report_Revision_1 Bjarki Eldon, PhD -- 1/6/2022 Reviewed

giac032_Reviewer_2_Report_Original_Submission Ryan Gutenkunst -- 12/3/2021 Reviewed

giac032_Reviewer_2_Report_Revision_1 Ryan Gutenkunst -- 12/16/2021 Reviewed

giac032_Supplemental_File

## Abbreviations

BC: British Columbia
bp: base pairs
CHS: Southern Han Chinese
CPU: central processing unit
EM: expectation maximization
EU: Europe
FIN: Finland
*F*_ST_
: fixation index
GATK: Genome Analysis Toolkit
GBR: British in England and Scotland
GUI: graphical user interface
kb: kilobase pairs
lcWGS: low-coverage whole-genome sequencing
NGS: next-generation sequencing
PBS: population branch statistic
QC: Quebec
RMSD: root mean square deviation
SB: standardized bias
SFS: site frequency spectrum
SNP: single-nucleotide polymorphism

## Competing Interests

The authors declare that they have no competing interests.

## Funding

T.S.K. is funded by Carlsberg grant CF19-0712. M.F. and A.M.S. are funded by The Leverhulme Research Project Grant RPG-2018-208. I.A. is funded by the Lundbeck Foundation Centre for Disease Evolution Grant id: R302-2018-2155. We acknowledge support from Erasmus+ programme and Imperial College FoNS European Partners award to M.F. and I.A.

## Authors' Contributions

T.S.K. developed the model. M.F., A.M.S., and I.A. ran all analyses. K.N.N. assisted with the analysis of real data. N.S.P. implemented the banded algorithm together with the generalized folding algorithm. All authors contributed to writing the manuscript.
