# Supplementary material for: Fast and accurate estimation of multidimensional site frequency spectra from low-coverage high-throughput sequencing data
Source: Gigascience. 2022 May 17;11:giac032. doi: 10.1093/gigascience/giac032 (PMC9112775; doi:10.1093/gigascience/giac032)
Supplement: giac032_GIGA-D-21-00341_Revision_2 [file giac032_giga-d-21-00341_revision_2.pdf]

GigaScience

Fast and accurate estimation of multidimensional site frequency spectra from low-coverage high-throughput sequencing data

--Manuscript Draft--

**Manuscript Number:**	GIGA-D-21-00341R2
**Full Title:**	Fast and accurate estimation of multidimensional site frequency spectra from low-coverage high-throughput sequencing data
**Article Type:**	Technical Note
**Funding Information:**	Carlsberg grant (CF19-0712) — Dr Thorfinn Sand Korneliussen<br>Leverhulme Trust (RPG-2018-208) — Dr Alex Mas-Sandoval, Dr Matteo Fumagalli<br>Erasmus+ — BSc Isin Altinkaya, Dr Matteo Fumagalli<br>HSE University Basic Research Program — Dr Thorfinn Sand Korneliussen<br>Imperial College FoNS European Partners — BSc Isin Altinkaya, Dr Matteo Fumagalli<br>Lundbeck Foundation Centre for Disease Evolution (R302-2018-2155) — BSc Isin Altinkaya

Funding	Recipient
Carlsberg grant (CF19-0712)	Dr Thorfinn Sand Korneliussen
Leverhulme Trust (RPG-2018-208)	Dr Alex Mas-Sandoval<br>Dr Matteo Fumagalli
Erasmus+	BSc Isin Altinkaya<br>Dr Matteo Fumagalli
HSE University Basic Research Program	Dr Thorfinn Sand Korneliussen
Imperial College FoNS European Partners	BSc Isin Altinkaya<br>Dr Matteo Fumagalli
Lundbeck Foundation Centre for Disease Evolution (R302-2018-2155)	BSc Isin Altinkaya

Abstract:

Background: The site frequency spectrum summarises the distribution of allele frequencies throughout the genome, and it is widely used as a summary statistic to infer demographic parameters and to detect signals of natural selection. The use of high-throughput low-coverage DNA sequencing data can lead to biased estimates of the site frequency spectrum due to high levels of uncertainty in genotyping.
Results: Here we design and implement a method to efficiently and accurately estimate the multidimensional site frequency spectrum for large numbers of haploid or diploid individuals across an arbitrary number of populations, using low-coverage sequencing data. The method maximises a likelihood function that represents the probability of the sequencing data observed given a multi-dimensional site frequency spectrum using genotype likelihoods. Notably, it uses an advanced binning heuristic paired with an accelerated expectation-maximisation algorithm for a fast and memory-efficient computation, and can generate both unfolded and folded spectra and bootstrapped replicates for haploid and diploid genomes. Based on extensive simulations, we show that the new method requires remarkably less storage and is faster than previous implementations whilst retaining the same accuracy. When applied to low-coverage sequencing data from the fungal pathogen \textit{Neonectria neomacrospora}, results recapitulate the patterns of population differentiation generated using the original high-coverage data.
Conclusions: The new implementation allows for accurate estimation of population genetic parameters from arbitrarily large, low-coverage data sets, thus facilitating cost-effective sequencing experiments in model and non-model organisms.

**Corresponding Author:**	Thorfinn Sand Korneliussen, Ph.D<br>Natural History Museum of Denmark<br>Copenhagen, DENMARK
**Corresponding Author Secondary Information:**	
**Corresponding Author's Institution:**	Natural History Museum of Denmark
**Corresponding Author's Secondary Institution:**	
**First Author:**	Alex Mas-Sandoval
**First Author Secondary Information:**	

Powered by Editorial Manager® and ProduXion Manager® from Aries Systems Corporation

**Order of Authors:**	Alex Mas-Sandoval
	Nathaniel S. Pope
	Knud Nor Nielsen
	Isin Altinkaya
	Matteo Fumagalli
	Thorfinn Sand Korneliussen
**Order of Authors Secondary Information:**	
**Response to Reviewers:**	Dear Editor and reviewers. We added the RRID in last resubmission and we have answered to the last two comments from reviewer1 below:<br><br>1) we have now expanded the paragraph with the explanation and information of genotype likelihoods:<br><br>"Many genotype likelihood models exists \cite{samtools,gatk,atlas,soapsnp} and the canonical genotype likelihood model is shown below, M denotes sequencing depth, b_i is the nucleotide for the ith read and e_i is the associated error rate which is in practice given by the phred scaled base quality score of the nucleotides of the read:"<br><br>2) We have now expanded the paragraph:<br><br>"Given a sample of n haploids with sequence data D_j. Here D_j denotes all the information that goes into the calculation of the genotype likelihoods. This includes the sequenced bases, quality scores, position within the reads but possibly also the mapping quality and external information such as library preparation type. $G_j \in \{0,1\}$ are the (unknown) genotypesfor the jth haploid, the likelihood that the number of derived alleles in the sample equals i is,"<br><br>Best<br>Thorfinn<br><br>GIGA-D-21-00341R1<br>Fast and accurate estimation of multidimensional site frequency spectra from low-coverage high-throughput sequencing data<br>Alex Mas-Sandoval; Nathaniel S. Pope; Knud Nor Nielsen; Isin Altinkaya; Matteo Fumagalli; Thorfinn Sand Korneliussen<br>GigaScience<br><br>Dear Dr Korneliussen,<br><br>Your revised manuscript "Fast and accurate estimation of multidimensional site frequency spectra from low-coverage high-throughput sequencing data" (GIGA-D-21-00341R1) has been assessed by our reviewers. I am pleased to inform you that it is acceptable for publication in GigaScience, in principle.<br><br>Before we proceed, please address the minor, final reviewer's comments below.<br>Our data curators will also contact you shortly to prepare the supporting data for GigaDB. Please include the GigaDB citation (including doi), which you will get from our curators, in the bibliography and cite it by number from the "availability" section.<br><br>In addition, please register any new software application in the bio.tools and SciCrunch.org databases to receive RRID (Research Resource Identification Initiative ID) and biotoolsID identifiers, and include these in your manuscript. This will facilitate tracking, reproducibility and re-use of your tool. (If the ANGSD platform already has RRID and biotool accessions, you can also include this).<br><br>Once you have made the necessary corrections, please submit a revised manuscript online at:<br><br>https://www.editorialmanager.com/giga/

Powered by Editorial Manager® and ProduXion Manager® from Aries Systems Corporation

	If you have forgotten your username or password please use the "Send Login Details" link to get your login information. For security reasons, your password will be reset.<br><br>Please include a point-by-point within the 'Response to Reviewers' box in the submission system. Please ensure you describe additional experiments that were carried out and include a detailed rebuttal of any criticisms or requested revisions that you disagreed with. Please also ensure that your revised manuscript conforms to the journal style, which can be found in the Instructions for Authors on the journal homepage. If the data and code has been modified in the revision process please be sure to update the public versions of this too.<br><br>The due date for submitting the revised version of your article is 07 Apr 2022.<br><br>We look forward to receiving your revised manuscript soon.<br><br>Best wishes,<br><br>Hans Zauner<br>GigaScience<br>www.gigasciencejournal.com<br><br>Reviewer reports:<br>Reviewer #1: thank you for responding to the comments;<br><br>I have only a few very minor additional comments, which can be dealt with without going through another round of revision<br><br>1 : maybe explain b_i in your equation for a genotype likelihood; is b_i the base at site i ?<br><br>2: In the derivation of Eq 1, maybe relate 'sequence data D_j' more clearly to the genotypes G_j, i.e.<br>explain the format of each D_j<br><br><br>Reviewer #2: The authors have addressed my concerns.
**Additional Information:**	
**Question**	**Response**
Are you submitting this manuscript to a special series or article collection?	No
**Experimental design and statistics**<br><br><br>Full details of the experimental design and statistical methods used should be given in the Methods section, as detailed in our Minimum Standards Reporting Checklist. Information essential to interpreting the data presented should be made available in the figure legends.<br><br><br>Have you included all the information requested in your manuscript?	Yes
**Resources**	Yes

Powered by Editorial Manager® and ProduXion Manager® from Aries Systems Corporation

A description of all resources used, including antibodies, cell lines, animals and software tools, with enough information to allow them to be uniquely identified, should be included in the Methods section. Authors are strongly encouraged to cite Research Resource Identifiers (RRIDs) for antibodies, model organisms and tools, where possible.<br><br>Have you included the information requested as detailed in our Minimum Standards Reporting Checklist?	
**Availability of data and materials**<br><br>All datasets and code on which the conclusions of the paper rely must be either included in your submission or deposited in publicly available repositories (where available and ethically appropriate), referencing such data using a unique identifier in the references and in the "Availability of Data and Materials" section of your manuscript.<br><br>Have you have met the above requirement as detailed in our Minimum Standards Reporting Checklist?	Yes

Manuscript

Click here to access/download;Manuscript;Multi_SFS_paper-main.pdf

OXFORD (GIGA)^n SCIENCE

GigaScience, 2021, 1–8

doi: xx.xxxx/xxxx
Manuscript in Preparation
Technical Note

TECHNICAL NOTE

Fast and accurate estimation of multidimensional site frequency spectra from low-coverage high-throughput sequencing data

Alex Mas-Sandoval†[1], Nathaniel S. Pope†[2], Knud Nor Nielsen[3], Isin Altinkaya[4], Matteo Fumagalli[1,5] and Thorfinn Sand Korneliussen*[4,6]

[1]Department of Life Sciences, Silwood Park campus, Imperial College London, SL5 7PY, Ascot, U.K. and [2]Department of Entomology, The Pennsylvania State University, State College, Pennsylvania, U.S.A. and [3]Department of Plant and Environmental Sciences, University of Copenhagen, Thorvaldsensvej 40, 1871 Frederiksberg C, Denmark. and [4]GLOBE, Section for Geogenetics, Øster Voldgade 5-7, 1350, Copenhagen, Denmark. and [5]School of Biological and Behavioural Sciences, Queen Mary University of London, London, U.K. and [6]National Research University Higher School of Economics, 20 Myasnitskaya Ulitsa, 101000, Moscow, Russia.

*tskorneliussen@sund.ku.dk
†Contributed equally.

Abstract

Background The site frequency spectrum summarises the distribution of allele frequencies throughout the genome, and it is widely used as a summary statistic to infer demographic parameters and to detect signals of natural selection. The use of high-throughput low-coverage DNA sequencing data can lead to biased estimates of the site frequency spectrum due to high levels of uncertainty in genotyping. **Results** Here we design and implement a method to efficiently and accurately estimate the multidimensional joint site frequency spectrum for large numbers of haploid or diploid individuals across an arbitrary number of populations, using low-coverage sequencing data. The method maximises a likelihood function that represents the probability of the sequencing data observed given a multi-dimensional site frequency spectrum using genotype likelihoods. Notably, it uses an advanced binning heuristic paired with an accelerated expectation-maximisation algorithm for a fast and memory-efficient computation, and can generate both unfolded and folded spectra and bootstrapped replicates for haploid and diploid genomes. Based on extensive simulations, we show that the new method requires remarkably less storage and is faster than previous implementations whilst retaining the same accuracy. When applied to low-coverage sequencing data from the fungal pathogen *Neonectria neomacrospora*, results recapitulate the patterns of population differentiation generated using the original high-coverage data. **Conclusion** The new implementation allows for accurate estimation of population genetic parameters from arbitrarily large, low-coverage data sets, thus facilitating cost-effective sequencing experiments in model and non-model organisms.

Key words: site frequency spectrum; high-throughput sequencing; genotype likelihoods; next-generation sequencing; maximum likelihood; population genetics; threading.

Compiled on: January 10, 2022.
Draft manuscript prepared by the author.

Introduction

Over the last two decades, next-generation sequencing (NGS) technologies have allowed researchers to generate large amount of genomic data for both model and non-model species [1]. Across various experimental settings, low-coverage whole genome sequencing (lcWGS) is becoming one of the most popular approaches in population genomics studies [2], with short-read data being the most feasible option at the moment. At a fixed experimental budget, sequencing a larger sample size at the cost of decreasing the individual read depth has been the preferred strategy in population genetics, as it is associated with less biased estimates of notable parameters [3]. However, under these conditions, the high degree of uncertainty that inherently exists for lcWGS data prevents the assignment of individual genotypes and Single Nucleotide Polymorphisms (SNPs) [4].

To solve this issue, statistical methods that compute a probability measure for each of the possible genotypes (the genotype likelihoods) and integrate over these probabilities in the downstream analyses have been proposed [5]. In general a genotype likelihood is calculated independently for each individual for each site and is the probability of the read data D given the true unobserved genotype G, which in a diploid context is given by

$$L(G = \{A_1, A_2\} \mid D) \propto Pr(D \mid G = \{A_1, A_2\}),\ A_1, A_2 \in \{A, C, G, T\}$$

Many genotype likelihood models exists [6, 7, 8, 9] and the canonical genotype likelihood model is shown below, M denotes sequencing depth, b_i is the nucleotide for the ith read and e_i is the associated error rate which is in practice given by the phred scaled base quality score of the nucleotides of the read:

$$\begin{aligned}\Pr(D \mid G = A_1A_2) &= \prod_{i=1}^{M} \Pr(b_i \mid G = A_1A_2)\\ &= \prod_{i=1}^{M} \left(2^{-1}\Pr(b_i \mid A_1) + 2^{-1}\Pr(b_i \mid A_2)\right),\\ \Pr(b_i \mid A) &= \begin{cases} \frac{e_i}{3} & \text{if } b_i \neq A \\ 1 - e_i & \text{if } b_i = A \end{cases}.\end{aligned}$$

Previous studies have shown that summary statistics commonly used in population genetics can be reliably estimated from lcWGS data using genotype likelihoods [10, 11, 12, 13, 14, 15, 16, 17, 18, 19]. The calculation of these estimators is implemented in the dedicated software packages `ngsTools` [20] and ANGSD [21]. Whilst being regarded as the gold-standard toolkit for population genetic inferences from lcWGS data, these implementations tend to be computationally expensive and require a large file storage capacity when applied to large numbers of sequenced samples, limiting their scalability with modern experimental data sets.

The estimation of the site frequency spectrum (SFS) is one of the analyses most affected by poor scalability. The SFS is arguably one of the most important summary statistics of population genetic data, as it summarizes the distribution of allele frequencies throughout the genome. The SFS contains invaluable information on the demographic and adaptive processes that shaped the evolution of the population under investigation [22]. For instance, a SFS showing an over-representation of rare alleles is an indication of an expanding population, while bottleneck events tend to deplete low-frequency variants. Complex scenarios of repeated bottlenecks and gene flow may also generate an excess of rare alleles [23, 24]. Similarly, a locus targeted by positive selection will exhibit an excess of rare variants, while balancing selection will cause an increase of common (i.e. intermediate-frequency) alleles.

The calculation of the joint, or multidimensional, SFS allows for the inference of the evolutionary relationships between populations [25]. In fact, many statistical methods to estimate demographic parameters from population genetic data employ the multidimensional SFS as the sole input [26]. Additionally, widely-used metrics of genetic differentiation between populations can be directly calculated from the multidimensional SFS, including estimators of the fixation index (F_{ST}) and the population branch statistic (PBS) [27].

Here, we propose a method to efficiently estimate the multidimensional SFS (and statistics thereof) for an arbitrary number of populations of either haploids or diploids, given lcWGS data. We evaluate its performance over a range of experimental scenarios and describe its new features in terms of speed and data storage. This novel implementation greatly reduces the computational cost and storage requirements through an accelerated expectation maximization (EM) algorithm that uses a subset of sample allele frequency likelihoods for any given SNP, and allows for the calculation of F_{ST} and PBS values on-the-fly. As an illustration, we demonstrate the applicability of this tool by calculating metrics of genetic differentiation between strains of haploid fungus *Neonectria neomacrospora* from NGS data. This novel method is part of the ANGSD pipeline [21] available at https://github.com/ANGSD.

Materials and Methods

Fast calculation of site frequency likelihoods

We seek to compute likelihoods y of possible sample allele frequencies for a single site, given a set of genotype likelihoods across samples. For a sample of diploids with n individuals, y is a $2n + 1$ vector containing the likelihood of observing zero derived alleles, one derived allele, etc., up to $2n$ derived alleles. It follows that first and last elements of this vector represent monomorphic alleles. Each element of y is a very large combinatorial product and sum, even for moderate number of individuals n. A dynamic programming algorithm described in [5] and implemented in [21] computes the entire vector efficiently in $\mathcal{O}(n^2)$. Assuming that the likelihood vector is unimodal (which is frequently the case and easy to verify on-the-fly), Han *et al.* [19] proposed an algorithm that only updates entries around the mode, reducing cost to $\mathcal{O}(n)$. We have implemented this low-cost version of the original algorithm in ANGSD. We here emphasize that the novelty lies not in the development of the dynamic programming algorithm presented in [19], but in the extension of this to a haploid and multidimensional population context. To our knowledge, there is no other readily available implementation.

We have also developed an analogous algorithm for haploids, in which case y has $n + 1$ elements and $y[i]$ is the likelihood of i derived alleles in a sample of n haploids. y is initialized using the genotype likelihoods for the ancestral and derived states in the first haploid sample ($x_0^{(1)}$ and $x_1^{(1)}$ respectively) so that $y^{(1)} = [x_0^{(1)}, x_1^{(1)}]$, and then is incrementally updated with genotype likelihoods from subsequent samples: at the ith iteration, given the output $y^{(i-1)}$ from the previous iteration and the genotype likelihoods $x_0^{(i)}, x_1^{(i)}$ for the ith sample, the jth element of the updated likelihood vector is equal to,

$$\phi(i, j) = y^{(i)}[j] = \begin{cases} x_0^{(i)} y^{(i-1)}[0] & \text{if } j = 0 \\ x_1^{(i)} y^{(i-1)}[i-1] & \text{if } j = i \\ \frac{(i-j)}{i} x_0^{(i)} y^{(i-1)}[j] + \frac{j}{i} x_1^{(i)} y^{(i-1)}[j-1] & \text{otherwise,} \end{cases} \quad (1)$$

so that the length of y increases by one with each iteration (e.g. the superscript (i) indicates that the vector incorporates genotype likelihoods up to the ith sample and thus has $i+1$ elements). As for the diploid case, the full recursion (on n haploids) can be performed in $\mathcal{O}(n)$ by only updating the y in a band of allele frequencies wherein the likelihoods exceed some predefined threshold ϵ (algorithm 1). In the rare cases where the site frequency likelihoods are not unimodal, we revert to the original $\mathcal{O}(n^2)$ algorithm. The derivation for equation 1 is in the Supplementary Information.

Algorithm 1: Banded site frequency likelihood computation for haploids

```
Input: haploid genotype likelihoods x, tolerance ϵ, number of haploids n
Output: site frequency likelihoods y, band limits l and u
set l ← 0, u ← 1, y^(1)[0] ← x_0^(1), y^(1)[1] ← x_1^(1)
for i ∈ {2, 3, ..., n} do
    l ← l + 𝕀[x_1^(i) ≥ x_0^(i)]
    for l' ∈ {l, l − 1, ..., 0} do
        if ϕ(i, l') < ϵ then set l ← l' and break
    u ← u + 𝕀[x_1^(i) ≥ x_0^(i)]
    for u' ∈ {u, u + 1, ..., i} do
        if ϕ(i, u') < ϵ then set u ← u' and break
    for j ∈ {u, u − 1, ..., l} do
        y^(i)[j] ← ϕ(i, j)
return l, u, y^(n)[l], y^(n)[l + 1], ..., y^(n)[u]
Note: ϕ is shorthand for Equation 1
```

Applications of site frequency likelihoods

Given vectors of site frequency likelihoods for sites across an arbitrarily large genomic region such as a segment of a chromosome, we can obtain a maximum likelihood estimate of the associated SFS (or its multi-dimensional analogue for multiple populations) via EM [21]. Many statistics of interest are either linear combinations of elements of the SFS (e.g. various estimators of the population-scaled mutation rate θ) or are ratios involving such linear combinations (e.g. F_{ST}). In many cases, we are interested in the local behaviour of these statistics within an interval around a locus of interest.

However, these local estimates may involve few segregating sites and thus may be particularly sensitive to low-coverage data and/or sequencing errors. We can reduce the variance in these local estimates by leveraging genome-wide information and using the globally-estimated SFS as the prior in an Empirical Bayes procedure [12]. Specifically, let $l_k^{(s)}, u_k^{(s)}$ and $y_k^{(s)}$ be the lower bound, upper bound, and likelihood band for the sample allele frequency at site s in population k, as output by Algorithm 1 or its diploid variant. Let $\Theta(i_1,\ldots,i_P)$ be a linear statistic of allele frequencies i across P populations, and z be the P-dimensional global SFS. The Empirical Bayes estimate of Θ across an arbitrarily small interval $\mathcal{M}$ is,

$$\hat{\Theta}_{EB} = \sum_{s\in\mathcal{M}} C_s^{-1} \sum_{i_1=l_1^{(s)}}^{u_1^{(s)}} \cdots \sum_{i_P=l_P^{(s)}}^{u_P^{(s)}} \Theta(i_1,\ldots,i_P) z[i_1,\ldots,i_P] \prod_{k=1}^{P} y_k^{(s)}[i_k]$$

$$C_s^{-1} = \sum_{i=l_1^{(s)}}^{u_1^{(s)}} \cdots \sum_{i=l_P^{(s)}}^{u_P^{(s)}} z[i_1,\ldots,i_P] \prod_{k=1}^{P} y_k^{(s)}[i_k]$$

For many organisms, the polarization of alleles into ancestral and derived states is not possible due to lack of ancestral genomic material or a recently-diverged outgroup. In this case, it is preferable to fold the SFS such that the frequency of the minor allele is estimated instead. To this end, we generalized the single-population probability model for the folded SFS in [5] to an arbitrary number of populations and derived an EM update for efficient optimization (Algorithm 2). Briefly, this is accomplished by introducing per-site latent variables that indicate the number of non-ancestral alleles in the sample and whether the site is correctly polarized, then taking the expectation of the joint log probability function with regard to these latent variables to find the EM update [28, 21] (further details are in the Supplementary Information). Local statistics that are symmetric with regard to allele polarization may then be estimated using the (global) folded SFS and the Empirical Bayes procedure described above.

Algorithm 2: EM update for folded multi-dimensional site frequency spectrum

```
Input: site frequency likelihood band l_p^(s), u_p^(s), y_p^(s) for populations p/sites s, number of haploids n_p for populations p, P-dimensional folded SFS z
Output: updated SFS z'
𝓘 ← {(i_1, ..., i_P) : ⊗_{p=1}^P (0, 1, ..., n_p)}
z'[(i_p)] ← 0, ∀(i_p) ∈ 𝓘
for s ∈ {1, 2, ...} do
    a[(i_p)] ← 0, ∀(i_p) ∈ 𝓘
    𝓙 ← {(j_1, ..., j_P) : ⊗_{p=1}^P (l_p^(s), l_p^(s) + 1, ..., u_p^(s))}
    b ← 0
    for (j_p) ∈ 𝓙 do
        c ← 1 + ∏_k 𝕀[2(j_p)_k = n_k]
        if 2 ∑_k (j_p)_k > ∑_k n_k then
            (i_p) ← (n_1 − (j_p)_1, n_2 − (j_p)_2, ..., n_P − (j_p)_P)
        else
            (i_p) ← (j_p)
        a[(i_p)] ← a[(i_p)] + c × z[(i_p)] × ∏_k y_k^(s)[(j_p)_k]
        b ← b + c × z[(i_p)] × ∏_k y_k^(s)[(j_p)_k]
    for (i_p) ∈ 𝓘 do
        z'[(i_p)] ← z'[(i_p)] + b^{-1} a[(i_p)]
return z'
```

Benchmarking

To compare the computational performance of the new implementation with the existing method [21], we examined the elapsed real time of the site allele frequency likelihood calculation ("-doSaf"), disk usage of BGZF compressed site allele frequency likelihood files ("saf.gz"), maximum memory usage, and the elapsed real time of the maximum likelihood estimation of the SFS ("realSFS"). We performed these analyses on NGS data for 5, 10, 25, 50, 100, 150, and 200 samples from 1000 Genomes Project Phase 3 dataset [29] using chromosome 1. The dataset consists of 14 individuals from Southern Han Chinese (CHS) group, 99 individuals from Finnish in Finland (FIN) group, and 87 individuals from British in England and Scotland (GBR) group [29]. We emphasize that we are subsampling across all 200 individuals assuming they are from a single population: the purpose of this specific benchmark is to assess the computational performance rather than the accuracy of SFS estimation.

We used 5 replicates for each step in the analyses and re-

tained the lowest value for elapsed real times, to minimize the influence of concurrent processes on our multiuser system. All analyses were conducted on a Red Hat Enterprise Linux Server 7.7 (Maipo) with Intel(R) Xeon(R) Gold 6152 CPUs @ 2.10GHz (x86_64) for benchmarking purposes. The commands used are `angsd -b ${FILE} -anc ancestral.fq -doSaf 1 -gl 1 -r 1 -out ${FILE}` and `realSFS ${FILE}.saf.idx`.

Accuracy on simulated data

To test the accuracy of the new implementation for the estimation of the multidimensional SFS, we simulated 60 pseudo-chromosomes for three equally sampled populations under a realistic demographic model of recent human history [26] using the software `ms` [30]. Simulated data consisted of sequences that were 1/10 of the length of human chromosome 22 ($\approx$ 5 Mb) with realistic values of mutation and recombination rates. The command line used was ‘ms 60 1 -t 1935 -r 2167 5130456 -I 3 20 20 20 -n 1 1.682020 -n 2 3.736830 -n 3 7.292050 -eg 0 2 116.010723 -eg 0 3 160.246047 -ma x 0.881098 0.561966 0.881098 x 2.797460 0.561966 2.797460 x -ej 0.028985 3 2 -en 0.028985 2 0.287184 -ema 0.028985 3 x 7.293140 x 7.293140 x x x x x -ej 0.197963 2 1 -en 0.303501 1 1‘ with seed numbers ‘44349 37512 34833‘. The ‘ms‘ command produced 1 replicate of 60 sampled haplotypes (ms 60 1) for 3 populations with equal sample size (-I 3 20 20 20) with fixed mutation and recombination rates (-t 1935 -r 2167) scaled by the region length (5130456 bp). These values correspond to realistic average values of mutation [31] and recombination rates [32] in the human genome. The three populations experienced changes in effective sizes (switches -n, -eg and -en), gene flow (-ma and -ema), and splits (-ej) following a previously proposed demographic model [26]. Simulations can be run using ‘msprime‘ [33] with the program ‘mspms‘ which allows to replace ‘ms‘ commands. The simulation generated data for 12335 diallelic SNPs which were then converted into genotype likelihoods using the utility program called `msToGlf` found in the ANGSD software suite [21], for details regarding the simulation algorithm we refer to [12]. We choose an unrealistic high error rate of 1% to show the performance of our method in a worst case setting [34]. From these simulated genomes and SNPs, we generated 100 distinct replicates of genotype likelihood data for each tested scenario of average per-site read depth (1X, 2X, 10X and 20X) and considered only variables sites for ease of computation. For each replicate, we estimated SFS using ANGSD following the new implementation aforementioned, and compared the results at different depths and against the ground truth. We assessed performance by calculating the root mean squared deviation (RMSD) and standardised bias (SB), the latter being the difference of estimated and true values divided by the true value.

Application to real data

We analysed whole-genome sequencing data from the haploid fungal pathogen *Neonectria neomacrospora* [35]. We analysed 70 samples for three sampling areas, corresponding to British Columbia (BC, 6 samples), Quebec (QC, 15 samples) and Europe (EU, 49 samples) We filtered out reads with mapping quality lower than 30 and nucleotides with a base quality score lower than 20 (in Phred scale). We used ANGSD to estimate the multidimensional SFS for use as prior information in the local estimation of F_{ST} [36] and *PBS* [27] in overlapping sliding windows of 20kbp with a step of 2kbp. To assess the accuracy at lower sequencing depth, we repeated the analyses on a randomly downsampled data set where we retained only 25% of sequenced reads.

Results

Computational performance

We first compared the computational performance between the original and new (labeled ’banded’) implementation for estimating the site frequency spectrum at different sample sizes. We observe an almost linear increase of runtime and memory usage with the number of samples using the original implementation (Table 1). On the other hand, we observe a lower disk and memory usage and runtime for large sample sizes using the new banded implementation, which no longer exhibits a proportional increase of memory with sample size.

Estimation of site frequency spectra

We estimated multi-dimensional SFS from simulated sequencing data and compared results across different sequencing

Table 1. Benchmarking

		doSaf		realSFS	
Sample size	Version	Time (minutes)	File size (GB)	Time (minutes)	Memory usage (GB)
5	original	76	7.3	5	15.3
	banded	79	2.2	7	10.5
10	original	135	16.8	13	21.7
	banded	122	3.9	11	12.0
25	original	279	39.2	81	47.1
	banded	238	5.6	66	12.7
50	original	547	64.3	123	85.3
	banded	421	6.5	88	13.4
100	original	1292	105.9	283	164.7
	banded	965	7.2	126	14.1
150	original	2055	142.3	315	244.2
	banded	1342	8.0	156	15.1
200	original	2991	162.0	492	323.7
	banded	2022	8.0	178	15.0

Benchmarking of original and the novel banded implementation of the SFS estimation using data from chromosome 1 of the individuals randomly selected from 1000 Genomes Project Phase 3 Dataset [29]. “realSFS time”: Minimum elapsed time among 5 replicates, “memory usage”: Maximum value of maximum memory usage among 5 replicates.

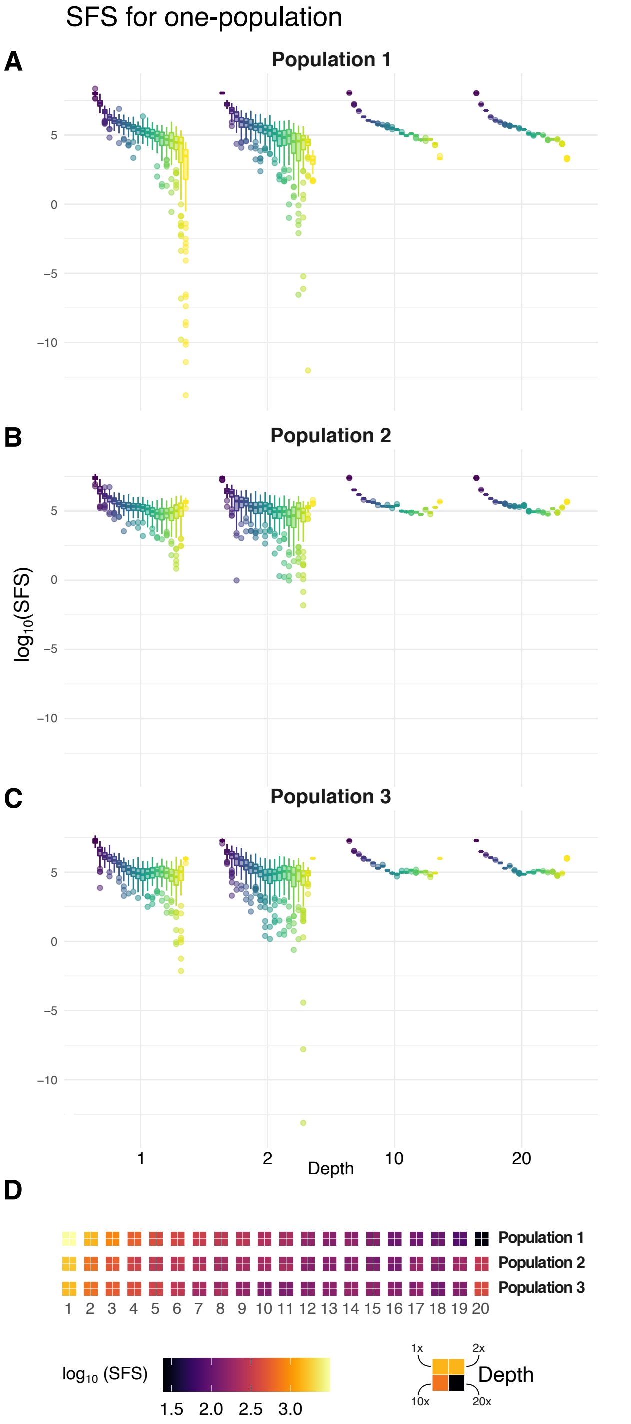

Figure 1. Estimation of one-dimensional SFS. Distribution of estimated SFS for individual populations (1 and 2 on top panel, 3 on bottom left panel) at four different sequencing average depths (1X, 2X, 10X and 20X) across 100 simulations. Each boxplot represents the distribution of the estimated number of alleles with a certain derived allele frequency, in $\log_{10}$ scale, across the 100 simulated data replicates. Bottom right panel **D** show a four-tile plot of the mean values of the distributions where each tile corresponds to a different depth. Notice that we do not observe any difference in any four-tiles across any population or sequencing depth. The monomorphic positions are omitted in all the panels.

depths (Figures 1, 2 and S1-S28). For the interpretation of these figures, the high depth scenarios can be assumed to be the true SFS.

Table 2. Effect of tolerance values on estimated SFS. For each tested scenario, we calculated the average KL divergence over 100 repetitions between the 2D-SFS with tolerance equal to 0 and several alternative values.

SFS	Depth	Tolerance	average KL
unfolded	2	1e-4	3.51e-08
		1e-6	3.79e-12
		1e-8	1.035e-15
unfolded	5	1e-4	4.69e-08
		1e-6	4.75e-13
		1e-8	3.78e-16
unfolded	10	1e-4	6.32e-10
		1e-6	6.92e-14
		1e-8	3.011e-16
folded	2	1e-4	1.67e-08
		1e-6	2.011e-12
		1e-8	3.63e-16
folded	5	1e-4	2.69e-09
		1e-6	2.88e-13
		1e-8	1.15e-16
folded	10	1e-4	6.43e-10
		1e-6	6.67e-14
		1e-8	1.38e-16

We observe that the mean estimates of SFS across replicates do not differ between sequencing depths except for few private and low frequency bins, where lower depths tend to overestimate. Estimates from low depths (1X and 2X) present a larger variance in their distributions and, therefore, show higher RMSD than estimates from high depths (10X and 20X) (Figures 1, 2 S1, S4). This pattern is observed in one-dimensional, two-dimensional and three-dimensional SFS (S7 and S9).

Within each SFS, low frequencies exhibit high RMSD (Figures S1, S4), while high frequencies have higher absolute values of SB (Figures S2, S5) as result of having low counts. In two-dimensional and three dimensional SFS, most of the errors are concentrated in population-private (high absolute SB) or low frequency bins (high RMSD) (Figures S7-S10). We replicated all these findings for both unfolded and folded multi-dimensional spectra (Figures S14-S28).

Finally, the choice of tolerance for calculation of site frequency likelihood bands had a minimal impact on the estimated SFS, in both folded and unfolded cases (Table 2), as previously suggested [19].

Population differentiation in *Neonectria neomacrospora*

We employed the method described herein to estimate the SFS from whole-genome sequences of a fungal pathogen *Neonectria neomacrospora*. We analyzed 70 samples for three main sampling areas (BC, QC, EU) [35], and downsampled the original sequencing data to mimic the challenges associated to low-coverage settings. From the estimated SFS, we sought to estimate the levels of genetic differentiation, as measured by *PBS* [27] in sliding windows.

Results show that when BC is the target population in PBS calculation, we observe greater levels of differentiation (Figure 3) than those obtained when QC or EU are considered target populations, in line with recent findings [35]. We also highlighted outlier windows with exceptionally high values of PBS compared to the empirical distribution (Figure 3). Notably, we obtained similar results when employing the full coverage sequencing data (Figure S29), although the scale of PBS values differs.

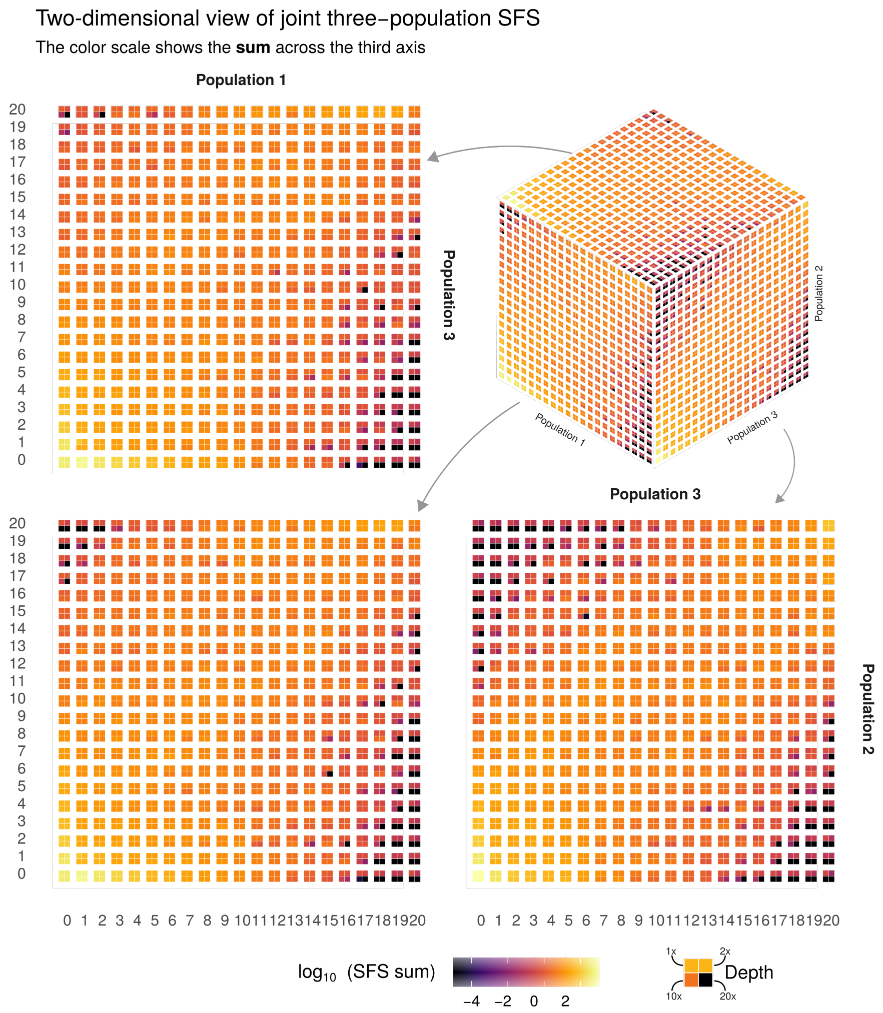

Figure 2. Estimation of three-dimensional SFS. Joint distribution of estimated SFS for three populations in four-tile plot, where each tile corresponds to a different depth. SFS is represented in three dimensions (top right panel) and as marginal two-dimensional SFS (top left and bottom panels) where the third axis is marginalised by its sum value. Values are reported in in $\log_{10}$ scale.

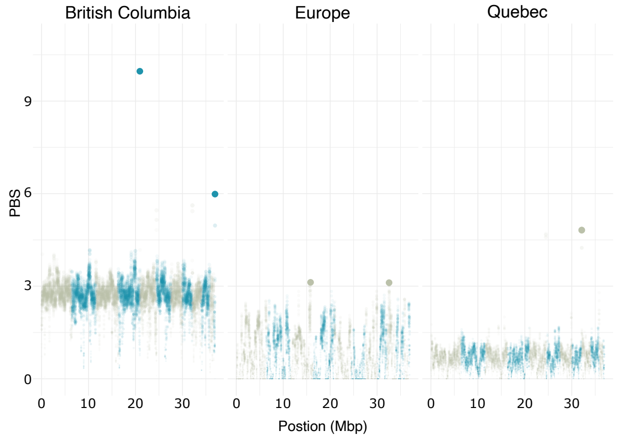

Figure 3. Sliding windows scan for genetic differentiation in three populations of *Neonectria neomacrospora*. We calculated *PBS* in sliding windows of 20kbp with a step of 2kbp. Each panel represents a separate scan where each population was considered the target and the remaining two controls. The highlighted points indicate windows with an empirical rank p-value lower than 10^{-3} in each population. Sequencing data were randomly downsampled to 25% of its original amount.

Conclusion

In this study, we present a novel implementation for the estimation of multi-dimensional SFS from lcWGS data. We show that the new method is faster and requires less memory and data storage than the currently available solution. Notably, these improvements do not come at the cost of accuracy, as estimated spectra display low error rates on simulated low-depth data or artificially downsampled real sequencing data.

We foresee several avenues for improving the methods and software developed for this study. For instance, additional metrics of genetic differentiation can be easily extracted from the estimated multi-dimensional SFS, such as genome divergence D_{XY} [37]. Likewise, extending this framework to an arbitrary ploidy would allow the estimation of SFS for polyploid species. Finally, a future user-friendly GUI for the ANGSD pipeline would make these analyses accessible to a broader user base.

The estimation of fundamental population genetic parameters over genomic intervals is crucial for elucidating how various evolutionary forces have acted to shape contemporary genetic polymorphism across species and populations. The development and implementation of sound statistical and bioinformatic methods that are robust to the uncertainty inherent in low-coverage sequence data and that leverage genome-wide information to improve local estimates are necessary for maintaining cost-effectiveness without sacrificing accuracy in the generation of large-scale population genomic data.

Implementation details

The program is implemented in a fast multi-threaded C++ program and takes as input either BAM/CRAM files or BCF/VCF files containing genotype likelihood files as produced from standard tools such as GATK [7] or SAMtools [6].

Availability of source code and requirements

- Project name: ANGSD/realSFS
- Project home page: e.g. https://github.com/ANGSD
- Operating system(s): Platform independent
- Programming language: C/C++
- Other requirements: htslib
- License: GPL
- RRID: *SCR*_021865

Availability of supporting data and materials

Supporting data and an archival copy of the code are available via the GigaScience repository GigaDB XXX.

List of abbreviations

SFS: site frequency spectrum; NGS: next generation sequencing; EM: expectation maximization; RMSD: root mean square deviation; SB: standardised bias; lcWGS: low-coverage whole genome sequencing; SNP: single nucleotide polymorphism; PBS: population branch statistic; F_{ST}: fixation index; BC: British Columbia; QC: Quebec; EU: Europe; CHS: Southern Han Chinese; FIN: Finland; GBR: British in England and Scotland.

Competing Interests

The author(s) declare that they have no competing interests.

Funding

TSK is funded by Carlsberg grant CF19-0712 and is prepared within the framework of the HSE University Basic Research Program. MF and AMS are funded The Leverhulme Research Project Grant RPG-2018-208. IA is funded by the Lundbeck Foundation Centre for Disease Evolution Grant id: R302-2018-2155. We acknowledge support from Erasmus+ programme and Imperial College FoNS European Partners award to MF and IA.

Author's Contributions

TSK developed the model. MF, AMS and IA ran all analyses. KNN assisted with the analysis of real data. NSP implemented the banded algorithm together with the generalized folding algorithm. All authors contributed to writing the manuscript.

Acknowledgements

We like to thank the many users of the ANGSD suite that have provided useful feedback. We would like to thank Lei Zhao for helpful feedback on mathematical description of the models and algorithms developed in this study. Preliminary analyses were conducted by Dean Ousby. We are grateful to Bjarki Eldon and Ryan Gutenkunst for reviewing the manuscript.

References

1. Levy SE, Myers RM. Advancements in Next-Generation Sequencing. Annual Review of Genomics and Human Genetics 2016;17(1):95–115. https://doi.org/10.1146/annurev-genom-083115-022413, pMID: 27362342.
2. Lou RN, Jacobs A, Wilder A, Therkildsen NO. A beginner's guide to low-coverage whole genome sequencing for population genomics. Molecular Ecology;n/a(n/a). https://onlinelibrary.wiley.com/doi/abs/10.1111/mec.16077.
3. Fumagalli M. Assessing the Effect of Sequencing Depth and Sample Size in Population Genetics Inferences. PLOS ONE 2013 11;8(11):1–11. https://doi.org/10.1371/journal.pone.0079667.
4. Nielsen R, Paul JS, Albrechtsen A, Song YS. Genotype and SNP calling from next-generation sequencing data. Nature reviews Genetics 2011 June;12(6):443—451. https://europepmc.org/articles/PMC3593722.
5. Nielsen R, Korneliussen T, Albrechtsen A, Li Y, Wang J. SNP calling, genotype calling, and sample allele frequency estimation from new-generation sequencing data. PloS one 2012;7(7):e37558.
6. Li H. A statistical framework for SNP calling, mutation discovery, association mapping and population genetical parameter estimation from sequencing data. Bioinformatics 2011 Nov;27(21):2987–2993.
7. McKenna A, Hanna M, Banks E, Sivachenko A, Cibulskis K, Kernytsky A, et al. The Genome Analysis Toolkit: a MapReduce framework for analyzing next-generation DNA sequencing data. Genome Res 2010 Sep;20(9):1297–1303.
8. Link V, Kousathanas A, Veeramah K, Sell C, Scheu A, Wegmann D, ATLAS: Analysis Tools for Low-depth and Ancient Samples. bioRxiv; 2017. https://doi.org/10.1101/105346.
9. Li R, Li Y, Fang X, Yang H, Wang J, Kristiansen K, et al. SNP detection for massively parallel whole-genome resequencing. Genome Res 2009 Jun;19(6):1124–1132.
10. Kim SY, Lohmueller KE, Albrechtsen A, Li Y, Korneliussen T, Tian G, et al. Estimation of allele frequency and association mapping using next-generation sequencing data. BMC bioinformatics 2011;12(1):231.
11. Fumagalli M, Vieira FG, Korneliussen TS, Linderoth T, Huerta-Sánchez E, Albrechtsen A, et al. Quantifying population genetic differentiation from next-generation sequencing data. Genetics 2013;195(3):979–992.
12. Korneliussen TS, Moltke I, Albrechtsen A, Nielsen R. Calculation of Tajima's D and other neutrality test statistics from low depth next-generation sequencing data. BMC bioinformatics 2013;14(1):1–14.
13. Vieira FG, Lassalle F, Korneliussen TS, Fumagalli M. Improving the estimation of genetic distances from Next-Generation Sequencing data. Biological journal of the Linnean Society 2016;117(1):139–149.
14. Vieira FG, Albrechtsen A, Nielsen R. Estimating IBD tracts from low coverage NGS data. Bioinformatics 2016 04;32(14):2096–2102. https://doi.org/10.1093/bioinformatics/btw212.
15. Fox EA, Wright AE, Fumagalli M, Vieira FG. ngsLD: evaluating linkage disequilibrium using genotype likelihoods. Bioinformatics 2019 03;35(19):3855–3856. https://doi.org/10.1093/bioinformatics/btz200.
16. Soraggi S, Rhodes J, Altinkaya I, Tarrant O, Balloux F, Fisher MC, et al. HMMploidy: inference of ploidy levels from short-read sequencing data. bioRxiv 2021;https://www.biorxiv.org/content/early/2021/06/30/2021.06.29.450340.
17. Lipatov M, Sanjeev K, Patro R, Veeramah KR. Maximum Likelihood Estimation of Biological Relatedness from Low Coverage Sequencing Data. bioRxiv 2015;https://www.

biorxiv.org/content/early/2015/07/29/023374.
18. Hanghøj K, Moltke I, Andersen PA, Manica A, Korneliussen TS. Fast and accurate relatedness estimation from high-throughput sequencing data in the presence of inbreeding. GigaScience 2019 04;8(5). https://doi.org/10.1093/gigascience/giz034, giz034.
19. Han E, Sinsheimer JS, Novembre J. Fast and accurate site frequency spectrum estimation from low coverage sequence data. Bioinformatics 2015 Mar;31(5):720–727.
20. Fumagalli M, Vieira FG, Linderoth T, Nielsen R. ngsTools: methods for population genetics analyses from next-generation sequencing data. Bioinformatics 2014 May;30(10):1486–1487.
21. Korneliussen TS, Albrechtsen A, Nielsen R. ANGSD: analysis of next generation sequencing data. BMC bioinformatics 2014;15(1):356.
22. Nielsen R. Molecular Signatures of Natural Selection. Annual Review of Genetics 2005;39(1):197–218. https://doi.org/10.1146/annurev.genet.39.073003.112420, pMID: 16285858.
23. Eldon B, Birkner M, Blath J, Freund F. Can the Site-Frequency Spectrum Distinguish Exponential Population Growth from Multiple-Merger Coalescents? Genetics 2015 01;199(3):841–856. https://doi.org/10.1534/genetics.114.173807.
24. Marchi N, Excoffier L. Gene flow as a simple cause for an excess of high-frequency-derived alleles. Evolutionary Applications 2020;13(9):2254–2263. https://onlinelibrary.wiley.com/doi/abs/10.1111/eva.12998.
25. Peter BM, Slatkin M. DETECTING RANGE EXPANSIONS FROM GENETIC DATA. Evolution 2013;67(11):3274–3289. https://onlinelibrary.wiley.com/doi/abs/10.1111/evo.12202.
26. Gutenkunst RN, Hernandez RD, Williamson SH, Bustamante CD. Inferring the Joint Demographic History of Multiple Populations from Multidimensional SNP Frequency Data. PLOS Genetics 2009 10;5(10):1–11. https://doi.org/10.1371/journal.pgen.1000695.
27. Yi X, Liang Y, Huerta-Sanchez E, Jin X, Cuo ZXP, Pool JE, et al. Sequencing of 50 human exomes reveals adaptation to high altitude. Science 2010;329(5987):75–78.
28. McLachlan GJ, Krishnan T. The EM algorithm and extensions, vol. 382. John Wiley & Sons; 2007.
29. A global reference for human genetic variation. Nature 2015 Sep;526(7571):68–74. https://doi.org/10.1038/nature15393.
30. Hudson RR. Generating samples under a Wright–Fisher neutral model of genetic variation . Bioinformatics 2002 02;18(2):337–338. https://doi.org/10.1093/bioinformatics/18.2.337.
31. Scally A. The mutation rate in human evolution and demographic inference. Current Opinion in Genetics Development 2016;41:36–43. https://www.sciencedirect.com/science/article/pii/S0959437X16301010, genetics of human origin.
32. Myers S, Bottolo L, Freeman C, McVean G, Donnelly P. A Fine-Scale Map of Recombination Rates and Hotspots Across the Human Genome. Science 2005;310(5746):321–324.
33. Baumdicker F, Bisschop G, Goldstein D, Gower G, Ragsdale AP, Tsambos G, et al. Efficient ancestry and mutation simulation with msprime 1.0. bioRxiv 2021;https://www.biorxiv.org/content/early/2021/09/21/2021.08.31.457499.
34. Stoler N, Nekrutenko A. Sequencing error profiles of Illumina sequencing instruments. NAR Genomics and Bioinformatics 2021 03;3(1). https://doi.org/10.1093/nargab/lqab019, lqab019.
35. Nielsen KN, Gopalakrishnan S, Korneliussen TS, Skovrind M, Sirén K, Petersen B, et al. Population genomics of the emerging forest pathogen Neonectria neomacrospora. bioRxiv 2020;p. 1–29. https://doi.org/10.1101/2020.12.07.407155.
36. Bhatia G, Patterson N, Sankararaman S, Price AL. Estimating and interpreting FST: The impact of rare variants. Genome Research 2013;23(9):1514–1521.
37. Nei M, Li WH. Mathematical model for studying genetic variation in terms of restriction endonucleases. Proceedings of the National Academy of Sciences 1979;76(10):5269–5273. https://www.pnas.org/content/76/10/5269.

Supplementary Material

Click here to access/download
Supplementary Material
Multi_SFS_paper-SI.pdf

Personal Cover
Click here to access/download;Personal Cover;Multi_SFS_cover_letter.pdf

Dear Editor,

We hereby submit our manuscript entitled “Fast and accurate estimation of multidimensional site frequency spectra from low-coverage high-throughput sequencing data” to be considered as an Application Note in GigaScience.

The manuscript describes a new method in the form of a software tool that allows researchers to infer various population genetic statistics related to the site frequency spectrum (SFS) from low coverage sequencing data. Summary statistics computed from allele frequencies based on genotype estimates from low-coverage sequencing data can be dramatically biased and lead to wrong conclusions, because genotypes are called with high uncertainty. Although various methods have been proposed to obtain SFS estimates from low-coverage data, their performance is computationally expensive and they require a large file storage capacity for large sample sizes.

Here, we propose a more efficient method to estimate the multidimensional SFS and related statistics from low-coverage sequencing data through an accelerated expectation maximization algorithm that uses a subset of sample allele frequency likelihoods for any given site. Through extensive simulations, we show that with this new implementation we dramatically reduce the computational cost and storage requirements without losing accuracy. Moreover, we apply our novel approach to a haploid species (a task not possible in previous implementations) and confirm findings of species divergence.

As more low-depth NGS data become available, we believe that our method will become an important and widely-used toolkit in population genomics and evolutionary biology. The software has been made available as a fast stand alone open source program (available on Github) and allows for most of the multiple input formats that are used within the field.

We are looking forward to hearing from you.

Yours sincerely,

Alex Mas-Sandoval, Nathaniel S. Pope, Knud Nor Nielsen, Isin Altinkaya, Matteo Fumagalli and Thorfinn Sand Korneliussen.
