# Supplementary material for: Fast and accurate estimation of multidimensional site frequency spectra from low-coverage high-throughput sequencing data
Source: Gigascience. 2022 May 17;11:giac032. doi: 10.1093/gigascience/giac032 (PMC9112775; doi:10.1093/gigascience/giac032)
Supplement: giac032_Supplemental_File [file giac032_supplemental_file.pdf]

Derivation of Equation 1

Given a sample of n haploids with sequence data D_j. Here D_j denotes all the information that goes into the calculation of the genotype likelihoods. This includes the sequenced bases, quality scores, position within the reads but possibly also the mapping quality and external information such as library preparation type. $G_j \in \{0,1\}$ are the (unknown) genotypesfor the jth haploid, the likelihood that the number of derived alleles in the sample equals i is,

$$\begin{aligned}
y_i &= \Pr(D_1, \ldots, D_n \mid \sum_{j=1}^{n} G_j = i) \\
&= \sum_{g \in \Omega_i^n} \Pr(G_1 = g_1, \ldots, G_n = g_n \mid \sum_{j=1}^{n} G_j = i) \prod_{j=1}^{n} \Pr(D_j \mid G_j = g_j) \\
&= \sum_{g \in \Omega_i^n} \frac{1}{|\Omega_i^n|} \prod_{j=1}^{n} x_{g_j}^{(j)}
\end{aligned}$$

where $\Omega_i^n = \{(g_1, \ldots, g_n) : \sum_k g_k = i, g_k \in \{0,1\}\}$ is the set of all binary tuples of length n that sum to i (e.g all collections of n genotypes consistent with a derived allele count of i), with cardinality $|\Omega_i^n| = \binom{n}{i}$. The expression $x_g^{(j)} \equiv \Pr(D_j \mid G_j = g)$ is the likelihood that the genotype of the jth sample is $g \in \{0,1\}$, as detailed in the main text. The substitution on the last line assumes that given a sample allele frequency, all possible genotype configurations are equally probable *a priori* (e.g. samples are exchangeable).

If the sample is polymorphic ($0 < i < n$), the set Ω_i^n can be divided into two parts, depending on the value of the nth genotype in each $g \in \Omega_i^n$, so that

$$\begin{aligned}
y_i &= \sum_{g \in \Omega_i^n} \frac{\delta_0^{g_n}}{|\Omega_i^n|} \prod_{j=1}^{n} x_{g_j}^{(j)} + \sum_{g \in \Omega_i^n} \frac{\delta_1^{g_n}}{|\Omega_i^n|} \prod_{j=1}^{n} x_{g_j}^{(j)} \\
&= x_0^{(n)} \frac{|\Omega_i^{n-1}|}{|\Omega_i^n|} \sum_{g \in \Omega_i^{n-1}} \frac{1}{|\Omega_i^{n-1}|} \prod_{j=1}^{n-1} x_{g_j}^{(j)} + \\
&\quad x_1^{(n)} \frac{|\Omega_{i-1}^{n-1}|}{|\Omega_i^n|} \sum_{g \in \Omega_{i-1}^{n-1}} \frac{1}{|\Omega_{i-1}^{n-1}|} \prod_{j=1}^{n-1} x_{g_j}^{(j)} \\
&= x_0^{(n)} \frac{|\Omega_i^{n-1}|}{|\Omega_i^n|} y_i^{\backslash n} + x_1^{(n)} \frac{|\Omega_{i-1}^{n-1}|}{|\Omega_i^n|} y_{i-1}^{\backslash n} \\
&= \frac{(n-i)}{n} x_0^{(n)} y_i^{\backslash n} + \frac{i}{n} x_1^{(n)} y_{i-1}^{\backslash n}
\end{aligned}$$

where $\delta_b^a = 1$ if $a = b$ and 0 otherwise and $y^{\backslash n}$ is the likelihood vector calculated after omitting the nth haploid from the sample. If the sample is monomorphic for the ancestral/derived allele ($i = 0$ or $i = n$), then the nth genotype is ancestral/derived by definition, and so

$$\begin{aligned}
y_0 &= x_0^{(n)} \frac{|\Omega_0^{n-1}|}{|\Omega_0^n|} y_0^{\backslash n} = x_0^{(n)} y_0^{\backslash n} \\
y_n &= x_1^{(n)} \frac{|\Omega_{n-1}^{n-1}|}{|\Omega_n^n|} y_{n-1}^{\backslash n} = x_1^{(n)} y_{n-1}^{\backslash n}.
\end{aligned}$$

A slight change in notation gives the recursion in Equation 1 of the main text.

Derivation of EM update in Algorithm 2

Let z be the joint site frequency spectrum (SFS) for P populations: $z[i_1, \ldots, i_P]$ is the probability that a randomly selected biallelic site has derived allele counts equal to i_1 in the first population, i_2 in the second population, etc. Let $n = (n_1, \ldots, n_P)$ contain the number of sampled chromosomes in each population (e.g. haploids), so that the dimension of z is $(n_1 + 1) \times \cdots \times (n_P + 1)$. The folded P-dimensional SFS has elements,

$$\tilde{z}[i_1, \ldots, i_P] = \begin{cases} z[i_1, \ldots, i_P] + z[n_1 - i_1, \ldots, n_P - i_P] & \text{if } 2\sum_{j=1}^{P} i_j < \sum_{j=1}^{P} n_j \\ z[i_1, \ldots, i_P] & \text{if } 2\sum_{j=1}^{P} i_j = \sum_{j=1}^{P} n_j \\ 0 & \text{otherwise,} \end{cases}$$

that are the probabilities that a randomly selected site has *minor allele* counts equal to i_1 in the first population, i_2 in the second population, etc.

Given sequence data $D^{(s)}$ for biallelic sites $s = 1 \ldots S$, the likelihood of $\tilde{z}$ is

$$\mathcal{L}(\tilde{z} \mid D^{(s)}) = \prod_{s=1}^{S} \sum_{i \in \Xi^n} \tilde{z}[i_1, \ldots, i_P] \left(\frac{\prod_{j=1}^{P} y_j^{(s)}[i_j]}{2} + \frac{\prod_{j=1}^{P} y_j^{(s)}[n_j - i_j]}{2} \right),$$

where $\Xi^n = \{(i_1, \ldots, i_P) : 2\sum_{j=1}^{P} i_j \le \sum_{j=1}^{P} n_j, i_j \in \{0, 1, \ldots, n_j\}\}$ is the set of all possible minor allele counts in the populations; and $y_j^{(s)}[i]$ is the likelihood that there are i "derived" alleles in population j at site s (the polarization/orientation of $y_j^{(s)}$ is arbitrary but must be consistent across populations). This assumes that given the minor allele count, both possible polarizations are equally likely *a priori*. Assume that Ξ^n is lexicographically ordered so that $i^{(k)} \equiv \{\Xi^n\}_k$ is the kth possible minor allele count, and let $\tilde{z}_k$ denote the corresponding element of $\tilde{z}$. Introducing per-site latent variables $u_s \in \{1, \ldots, |\Xi^n|\}$ and $v_s \in \{0,1\}$ that respectively indicate the minor allele count and the polarization, the likelihood becomes:

$$\mathcal{L}(\tilde{z}, u, v \mid D^{(s)}) = \prod_{s=1}^{S} \prod_{k=1}^{|\Xi^n|} \prod_{j=1}^{P} (\tilde{z}_k)^{\delta_k^{u_s}} (y_j^{(s)}[i_j^{(k)}])^{\delta_0^{v_s} \delta_k^{u_s}} (y_j^{(s)}[n_j - i_j^{(k)}])^{\delta_1^{v_s} \delta_k^{u_s}},$$

where $\delta_b^a = 1$ if $a = b$ and 0 otherwise.

The EM objective function is,

$$\begin{aligned}
Q(\tilde{z}' \mid \tilde{z}) &= \mathbb{E}_{u,v|\tilde{z}}[\log \mathcal{L}(\tilde{z}', u, v \mid D^{(s)})] \\
&= \sum_{k=1}^{|\Xi^n|} \log(\tilde{z}'_k) \sum_{s=1}^{S} p_k^{(s)} + \text{constant,}
\end{aligned}$$

where

$$\begin{aligned}
p_k^{(s)} &= \Pr(u_s = k \mid \tilde{z}, D^{(s)}) \\
&= \frac{\tilde{z}_k \left(\prod_{j=1}^{P} y_j^{(s)}[i_j^{(k)}] + \prod_{j=1}^{P} y_j^{(s)}[n_j - i_j^{(k)}] \right)}{\sum_{l=1}^{|\Xi^n|} \tilde{z}_l \left(\prod_{j=1}^{P} y_j^{(s)}[i_j^{(l)}] + \prod_{j=1}^{P} y_j^{(s)}[n_j - i_j^{(l)}] \right)}
\end{aligned}$$

and thus the EM update is

$$\arg\max \tilde{z}'_k = \frac{\sum_{s=1}^{S} p_k^{(s)}}{\sum_{l=1}^{|\Xi^n|} \sum_{s=1}^{S} p_l^{(s)}}.$$

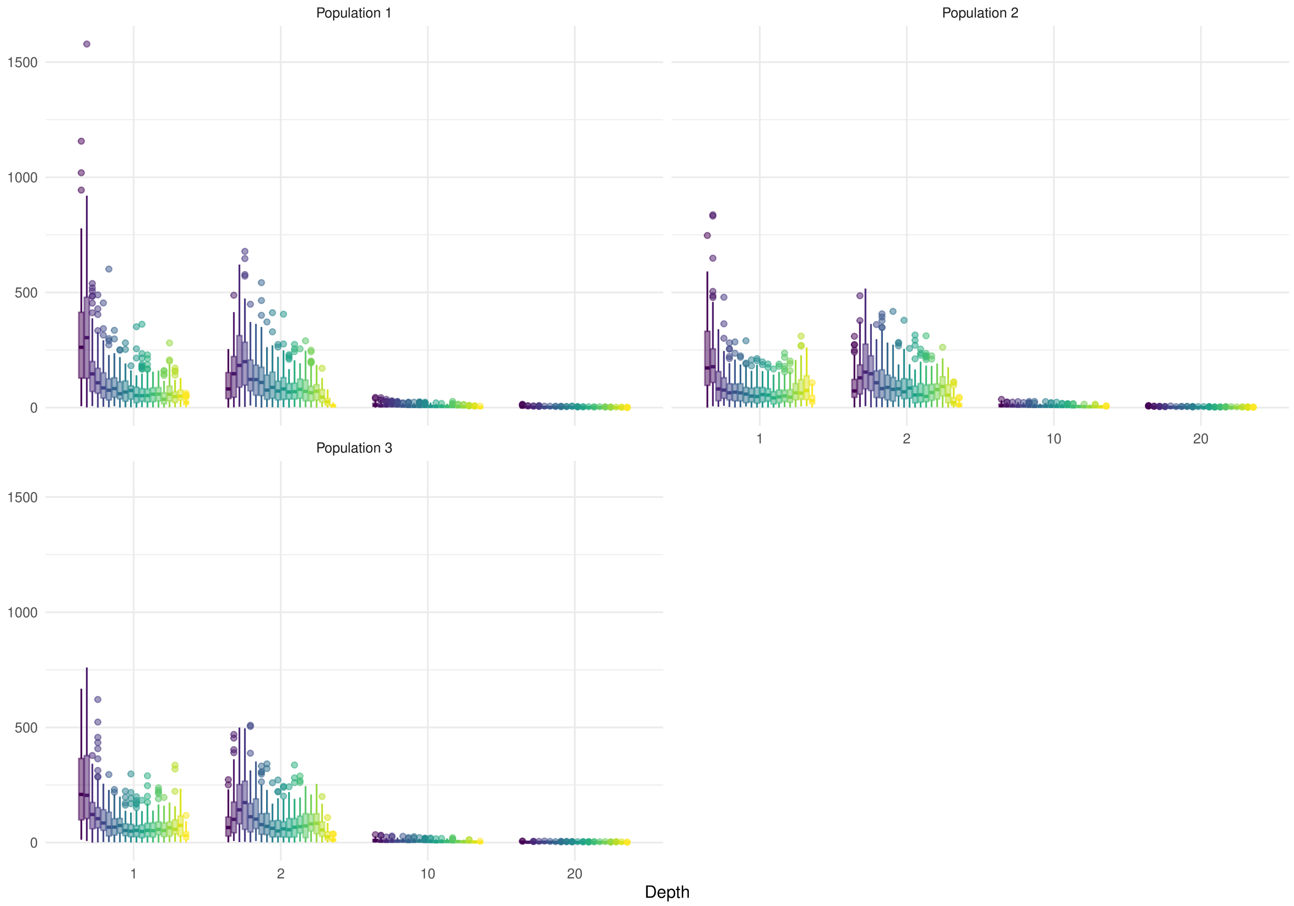

Fig S 1. Root mean squared deviation of population-based SFS values shown at Fig 1. Each panel represents the root mean squared deviation of the SFS for a different population. Each of the coloured distributions of the box plot shows the root mean squared deviation values for the log_{10} of the number of occurrences of each derived allele frequency.

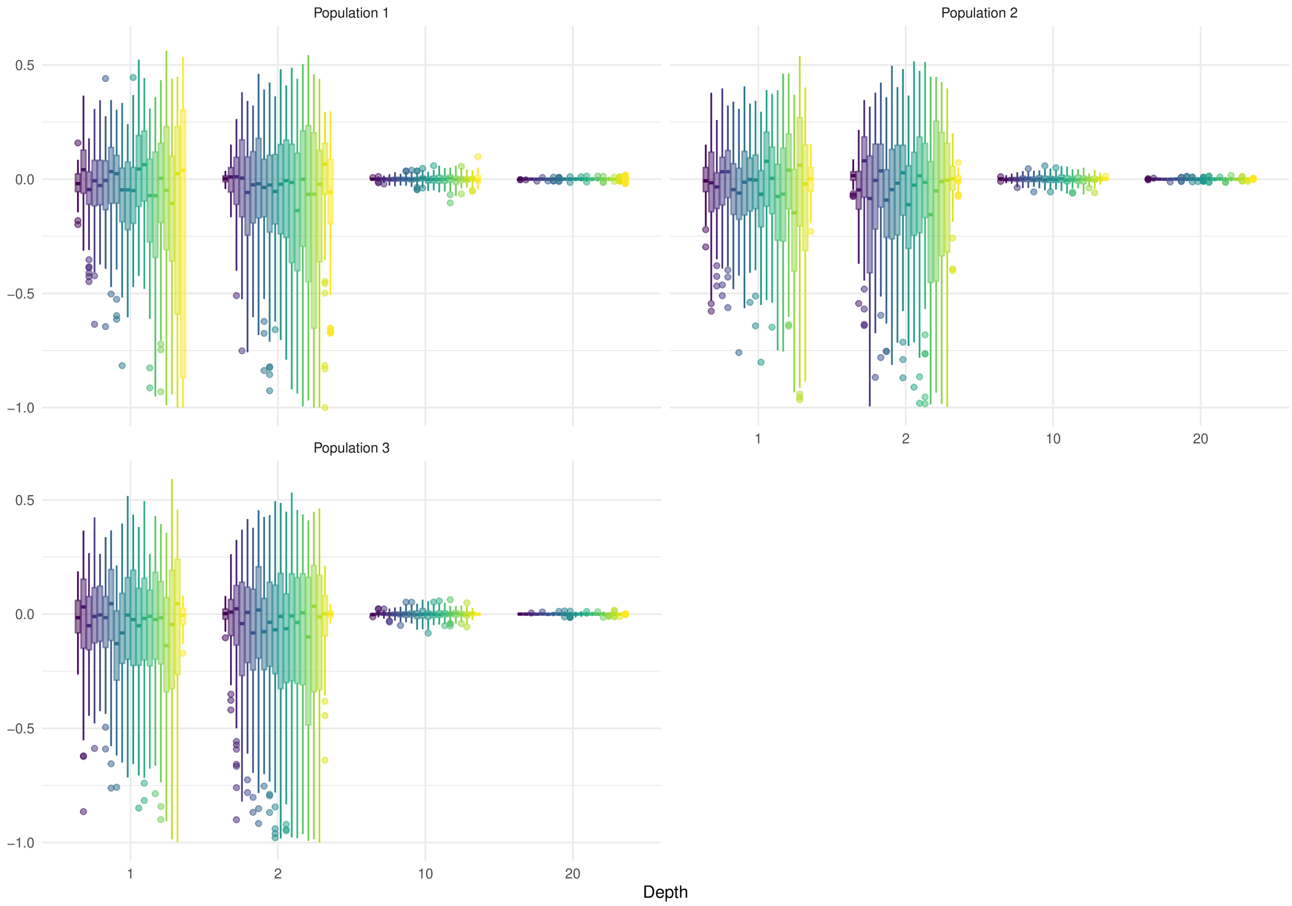

Fig S 2. Standardised bias of population-based SFS values shown at Fig 1. Each panel represents the standardised bias of the SFS for a different population, at different depths(1x,2x,10x and 20x). Each of the coloured distributions of the box plot shows the values for the standardised bias of the log_{10} of the number of occurrences of each derived allele frequency.

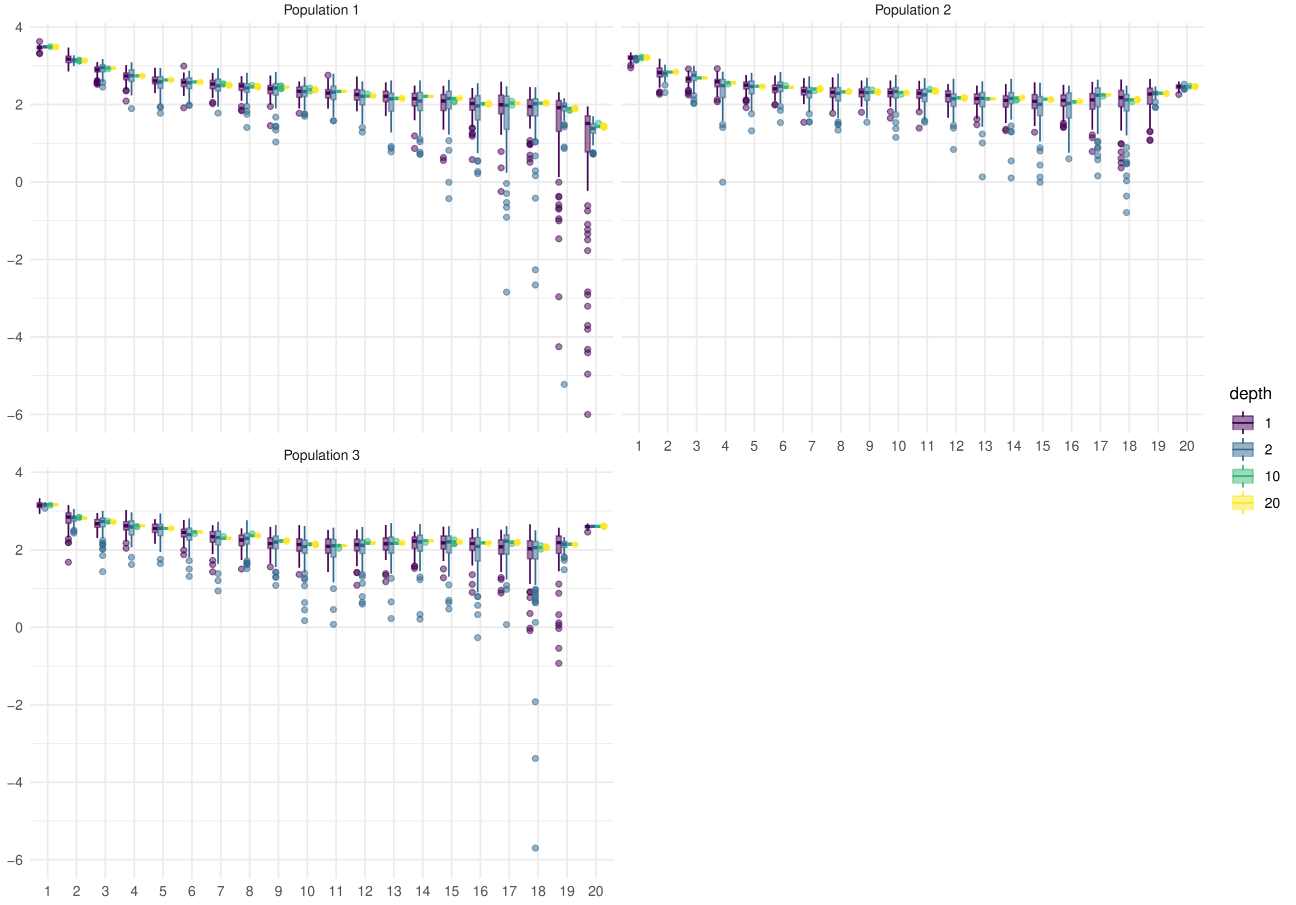

Fig S 3. Population-based SFS. Each panel represents the SFS for a different population, for each derived allele frequency. Each of the coloured distributions of the box plot shows the log_{10} of the number of occurrences in depths 1x, 2x, 10x and 20x.

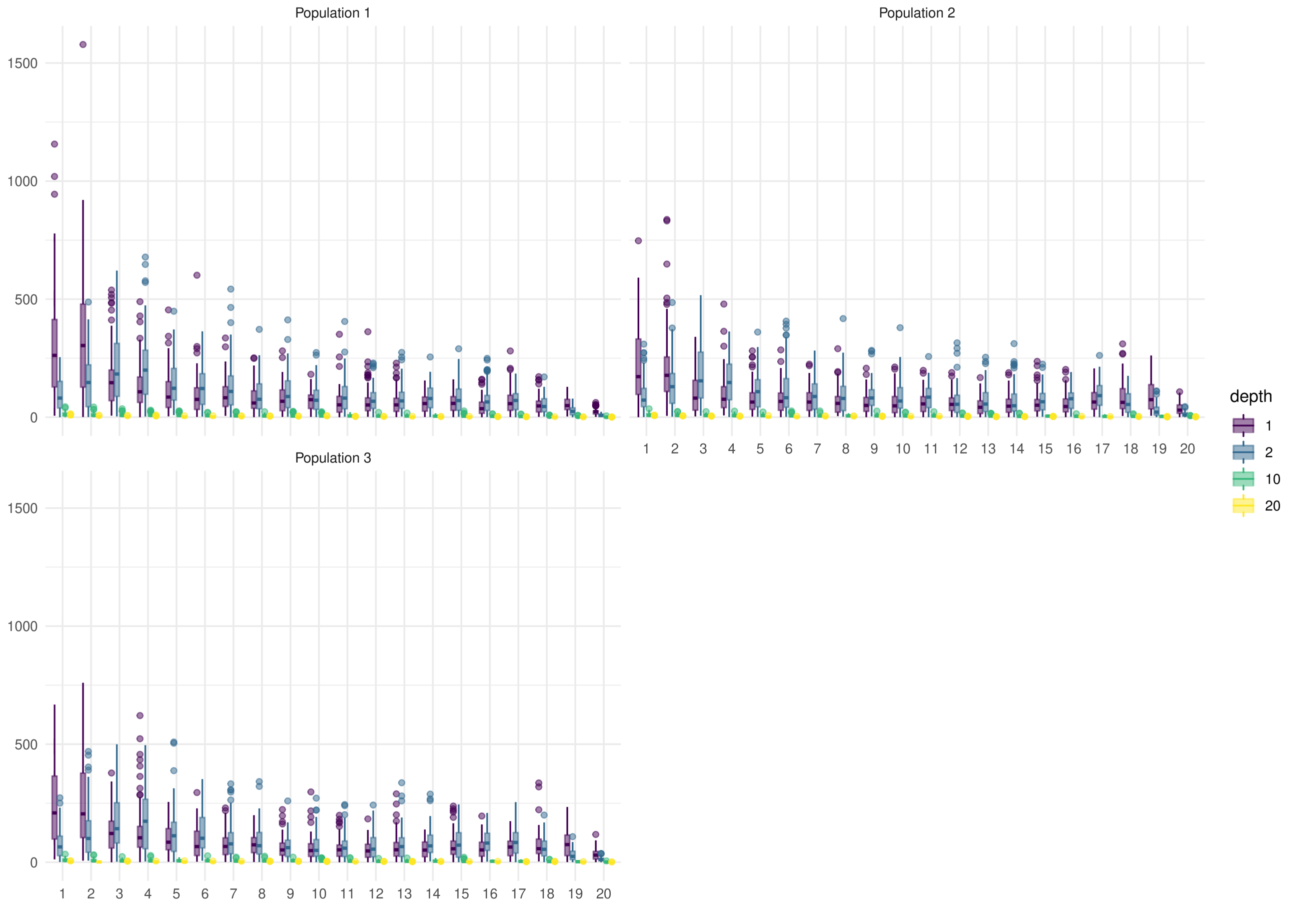

Fig S 4. Root mean squared deviation of population-based SFS values shown at Fig S3. Each panel represents the root mean squared deviation of the SFS for a different population, for each derived allele frequency. Each of the coloured distributions of the box plot shows root mean squared deviation of the log_{10} of the number of occurrences in depths 1x, 2x, 10x and 20x.

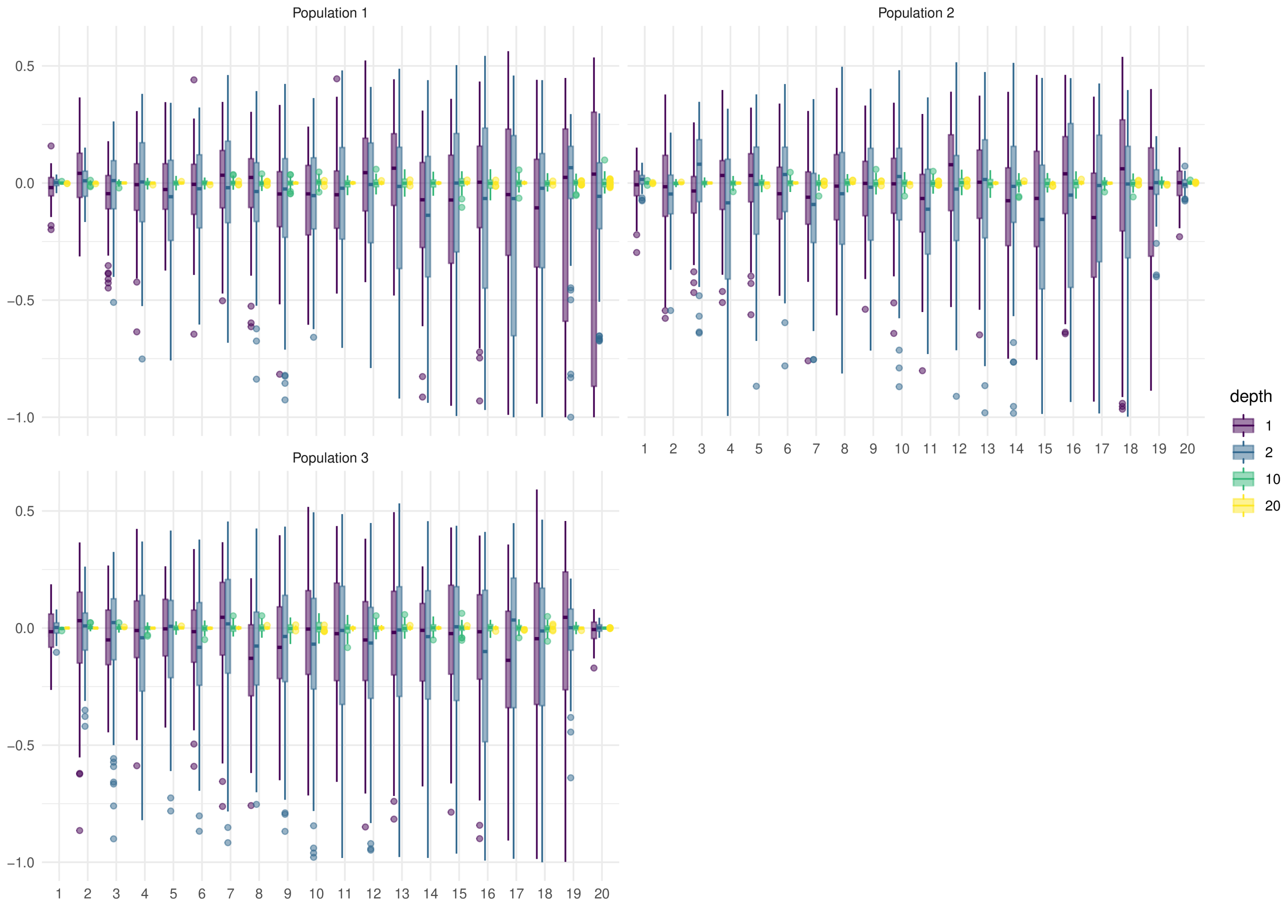

Fig S 5. Standardised bias of population-based SFS values shown at Fig S3. Each panel represents the standardised bias of the SFS for a different population, for each derived allele frequency. Each of the coloured distributions of the box plot shows the standardised bias of the log_{10} of the number of occurrences in depths 1x, 10x, 2x, 10x and 20x.

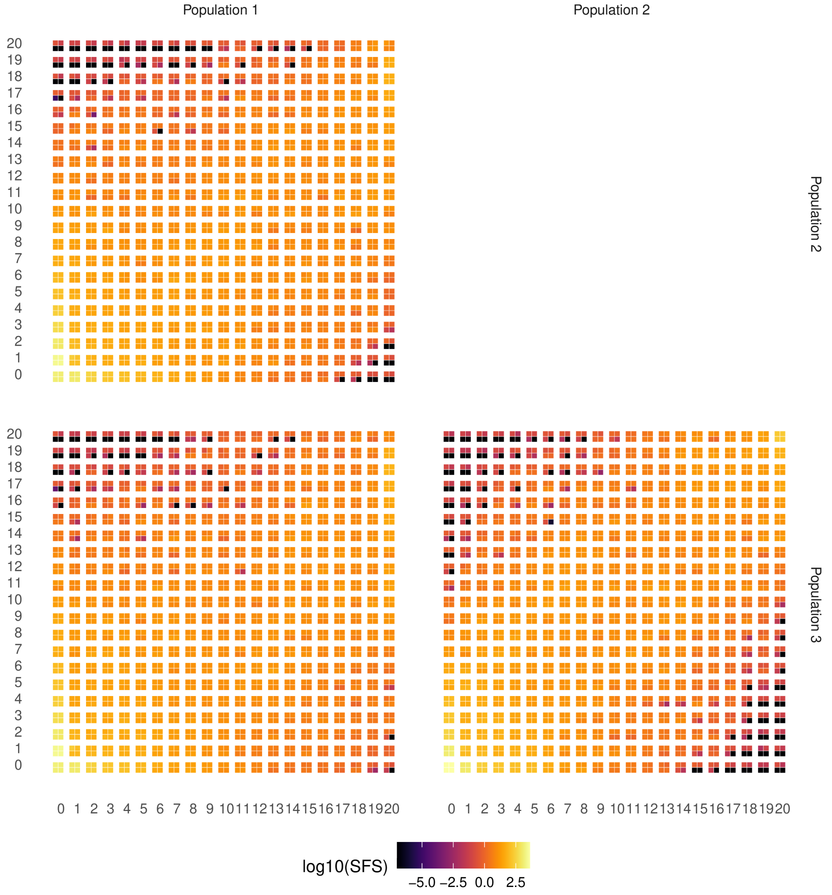

Fig S 6. Two-population joint SFS. Each panel represents the two-population joint SFS for a different pair of populations, for each derived allele frequency. Each color of the four-tile squares is according to the log_{10} of the number of occurrences at depths 1x, 2x, 10x and 20x, like in Fig 2

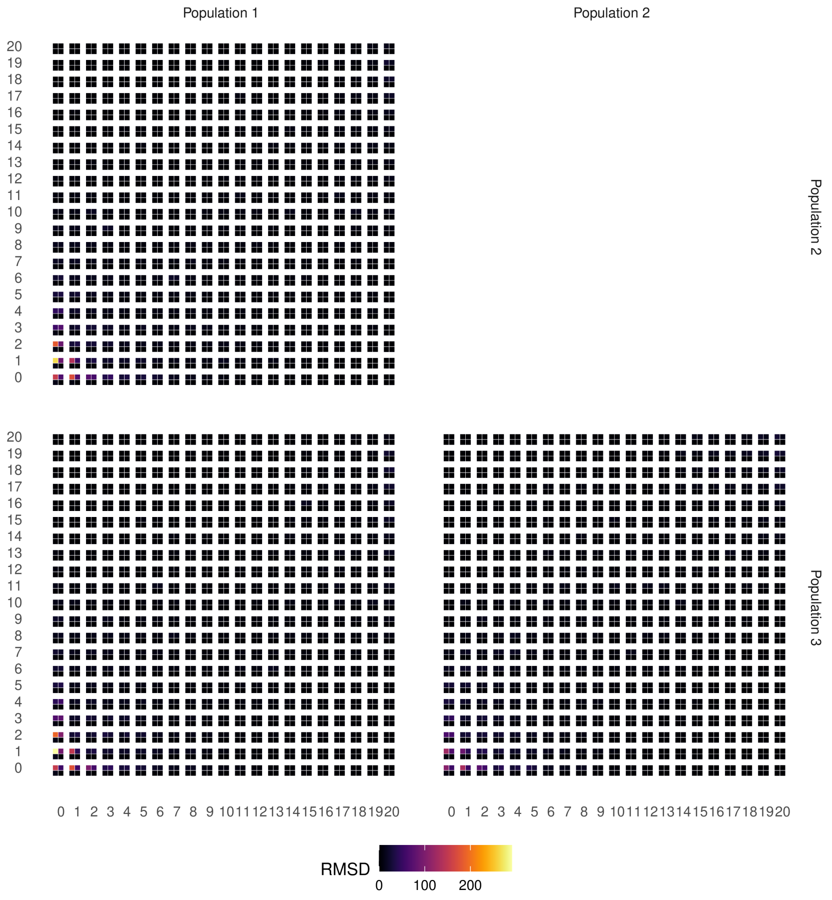

Fig S 7. Root mean squared deviation of the two-population joint SFS values shown at Fig S6. Each panel represents the root mean squared deviation of the two-population joint SFS for a different pair of populations, for each derived allele frequency. Each color of the four-tile squares is according to the root mean squared deviation of the log_{10} of the number of occurrences at depths 1x, 2x, 10x and 20x, like in Fig 2.

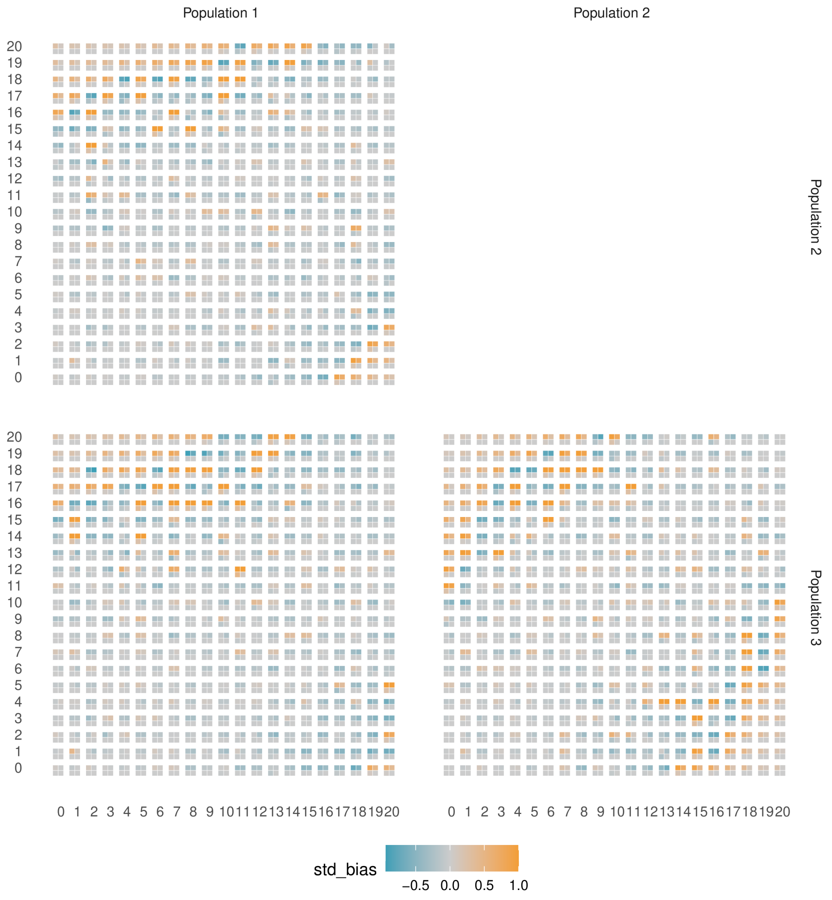

Fig S 8. Standardised bias of the Two-population Joint SFS values shown at Fig S6. Each panel represents the standardised bias of the two-population joint SFS for a different pair of populations, for each derived allele frequency. Each color of the four-tile squares is according to the standardised bias of the log_{10} of the number of occurrences at depths 1x, 2x, 10x and 20x, like in Fig 2.

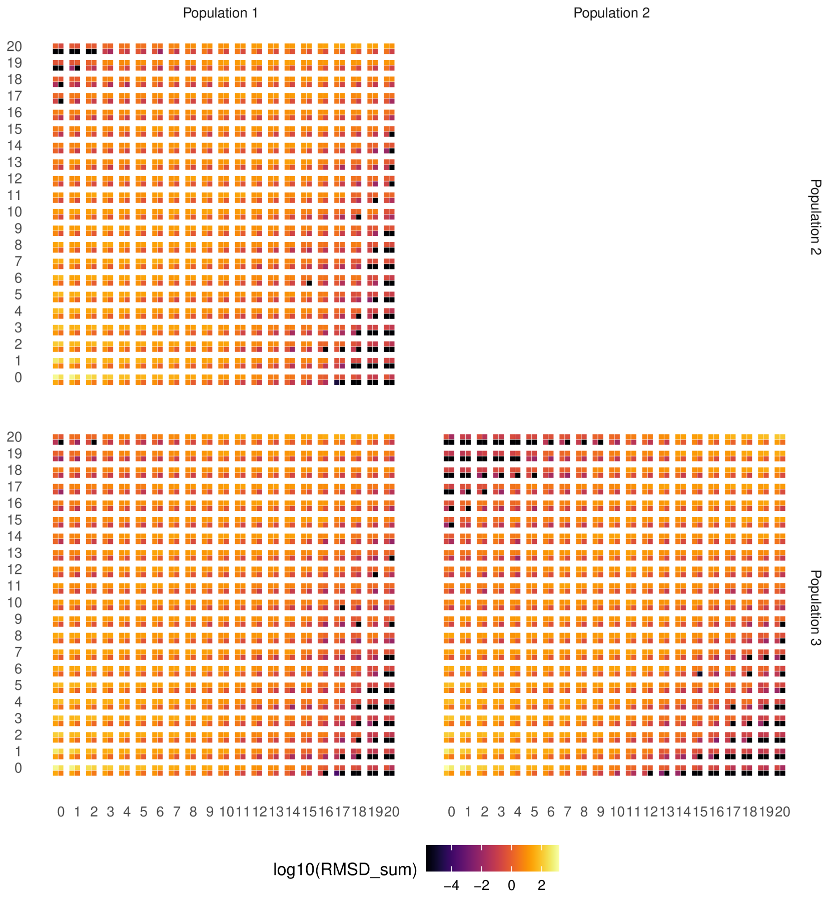

Fig S 9. Root mean squared deviation of the three-population joint SFS shown at Fig 2. Each panel represents the root mean squared deviation of the three-population joint SFS for a different pair of populations, for each derived allele frequency. Each color of the four-tile squares is according to the root mean squared deviation of the log_{10} of the **marginal sum** of the number of occurrences across the third axis at depths 1x, 2x, 10x and 20x, like in Fig 2.

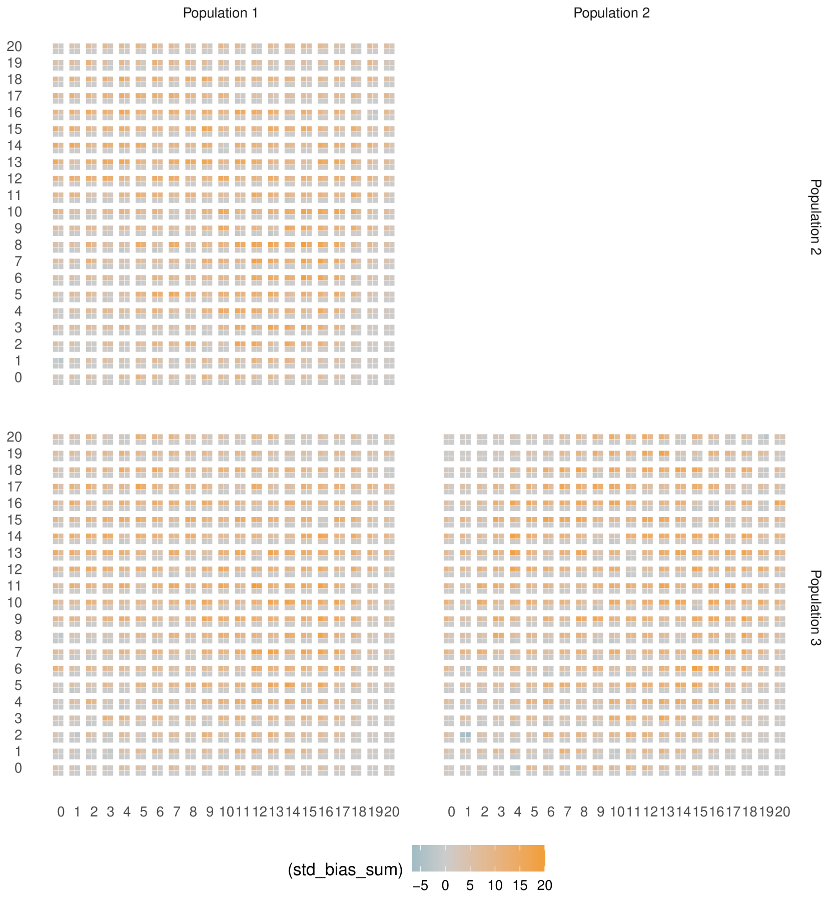

Fig S 10. Standardised bias of three-population joint SFS shown at Fig 2. Each panel represents the standardised bias of the three-population joint SFS for a different pair of populations, for each derived allele frequency. Each color of the four-tile squares is according to the standardised bias of the log_{10} of the **marginal sum** of the number of occurrences across the third axis at depths 1x, 2x, 10x and 20x, like in Fig 2.

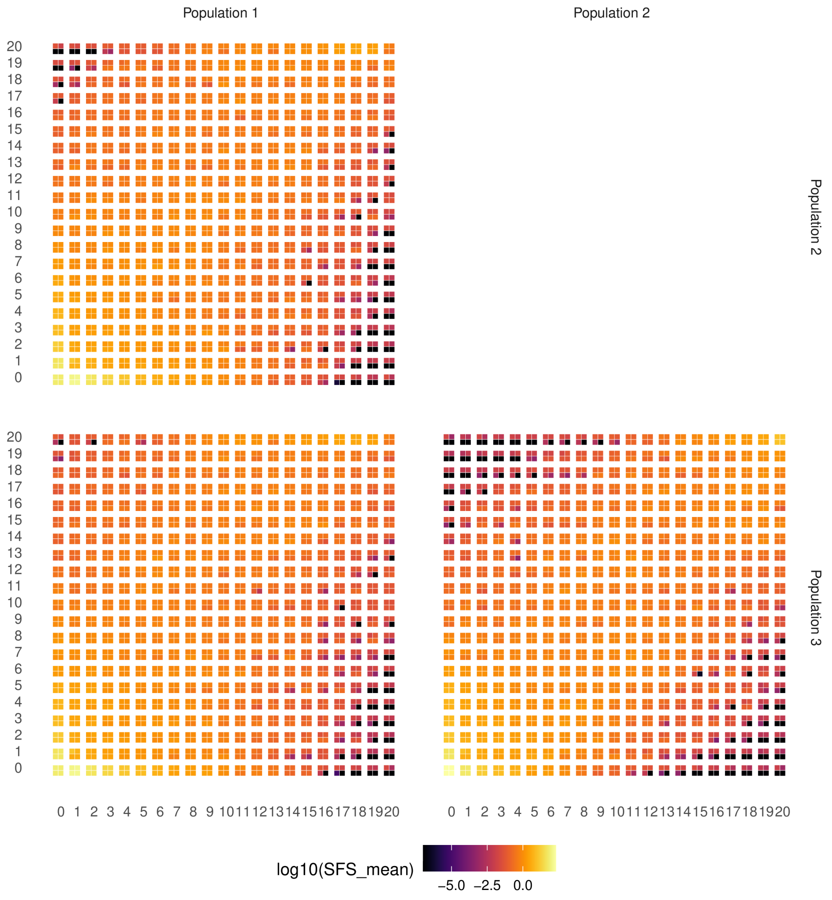

Fig S 11. Three-population joint SFS. Each panel represents the three-population joint SFS for a different pair of populations, for each derived allele frequency. Each color of the four-tile squares is according to the log_{10} of the **mean** of the number of occurrences across the third axis at depths 1x, 2x, 10x and 20x, like in Fig 2

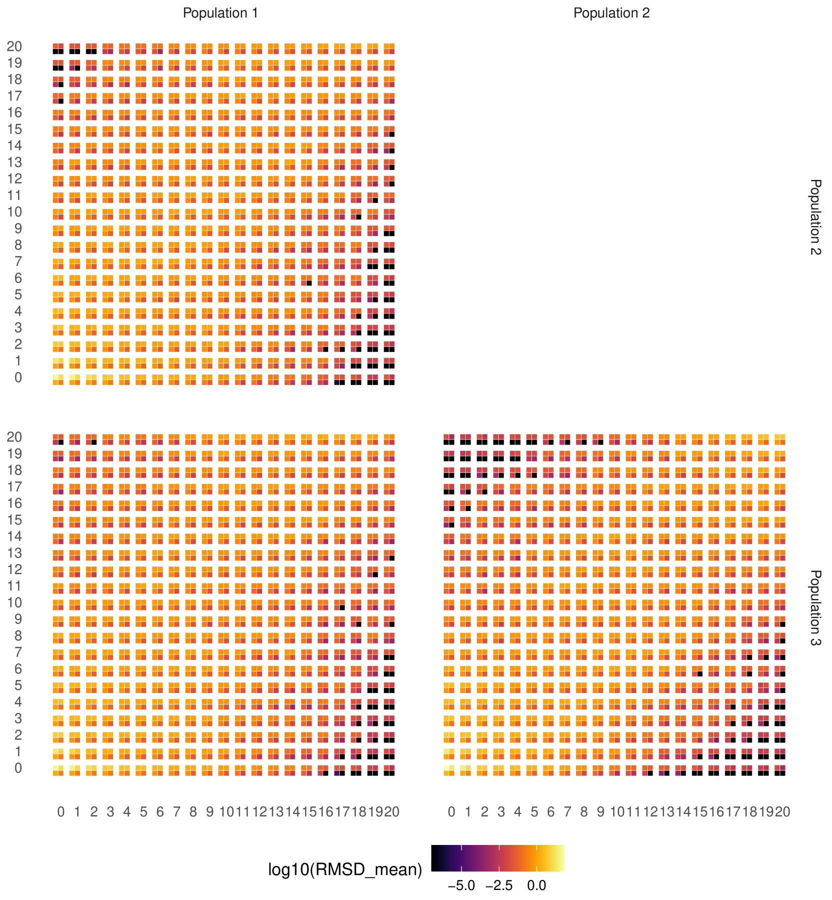

Fig S 12. Root mean squared deviation of the three-population joint SFS shown at Fig S11. Each panel represents the root mean squared deviation of the three-population joint SFS for a different pair of populations, for each derived allele frequency. Each color of the four-tile squares is according to the root mean squared deviation of the log_{10} of the **mean** of the number of occurrences across the third axis at depths 1x, 2x, 10x and 20x, like in Fig 2.

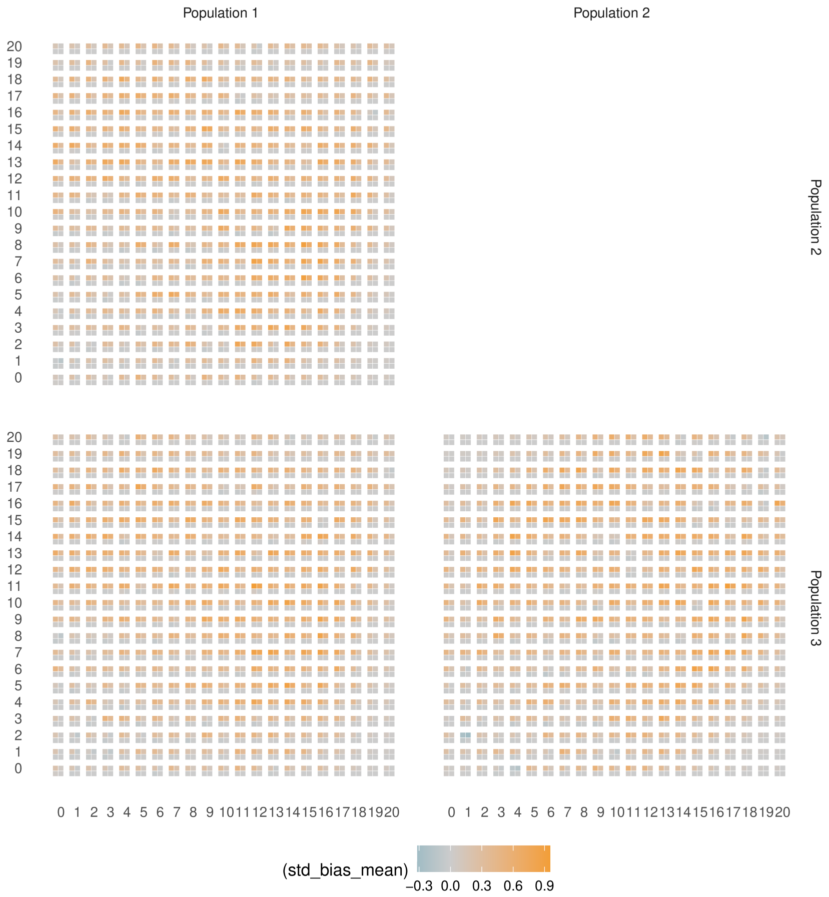

Fig S 13. Standardised bias of three-population joint SFS shown at Fig S11. Each panel represents the standardised bias of the three-population joint SFS for a different pair of populations, for each derived allele frequency. Each color of the four-tile squares is according to the standardised bias of the log_{10} of the **mean** of the number of occurrences across the third axis at depths 1x, 2x, 10x and 20x, like in Fig 2.

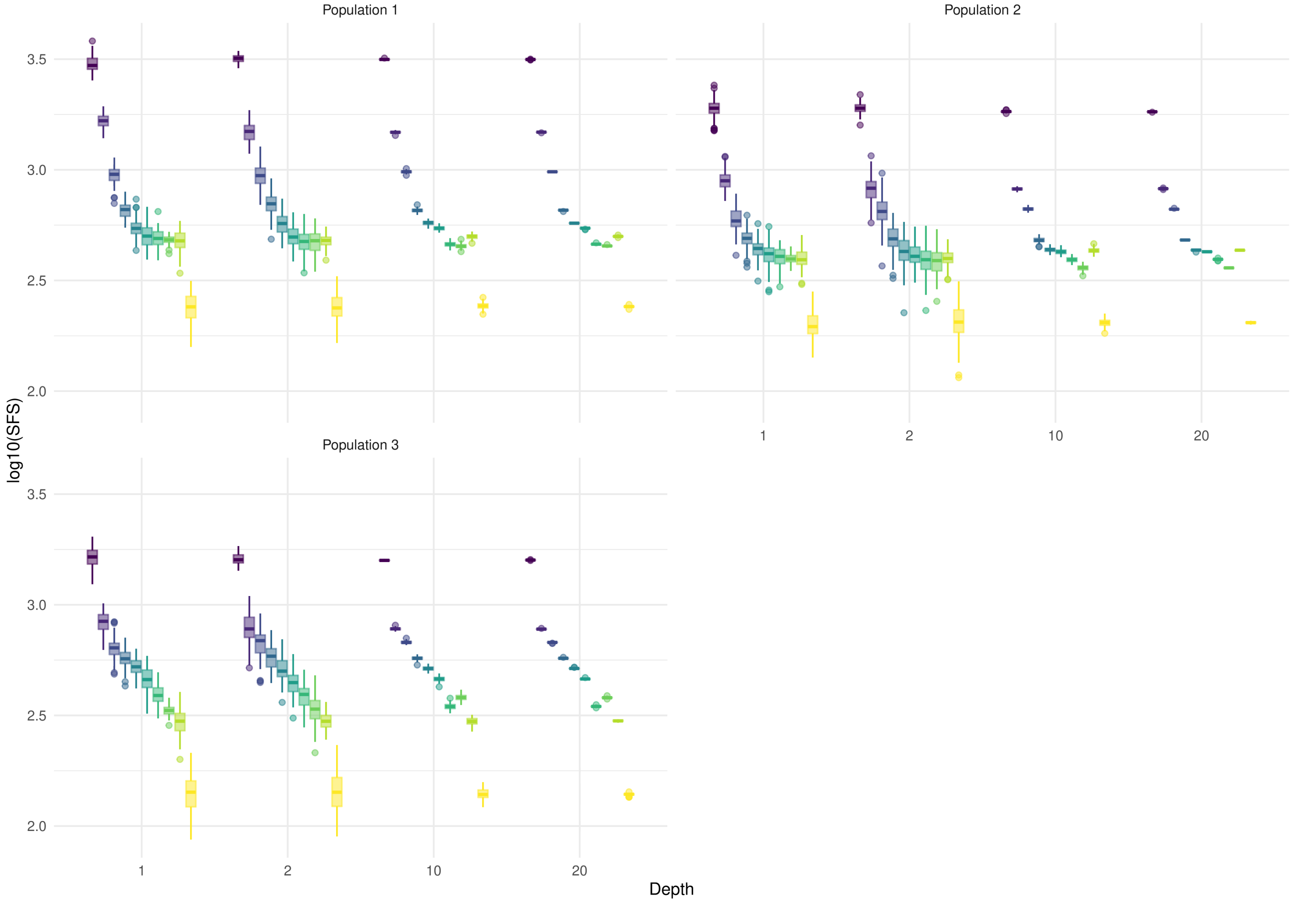

Fig S 14. Population-based folded SFS. Each panel represents the folded SFS for a different population, for depths 1x, 2x, 10x and 20x. Each of the coloured distributions of the box plot shows the values for the log_{10} of the number of each allele frequency.

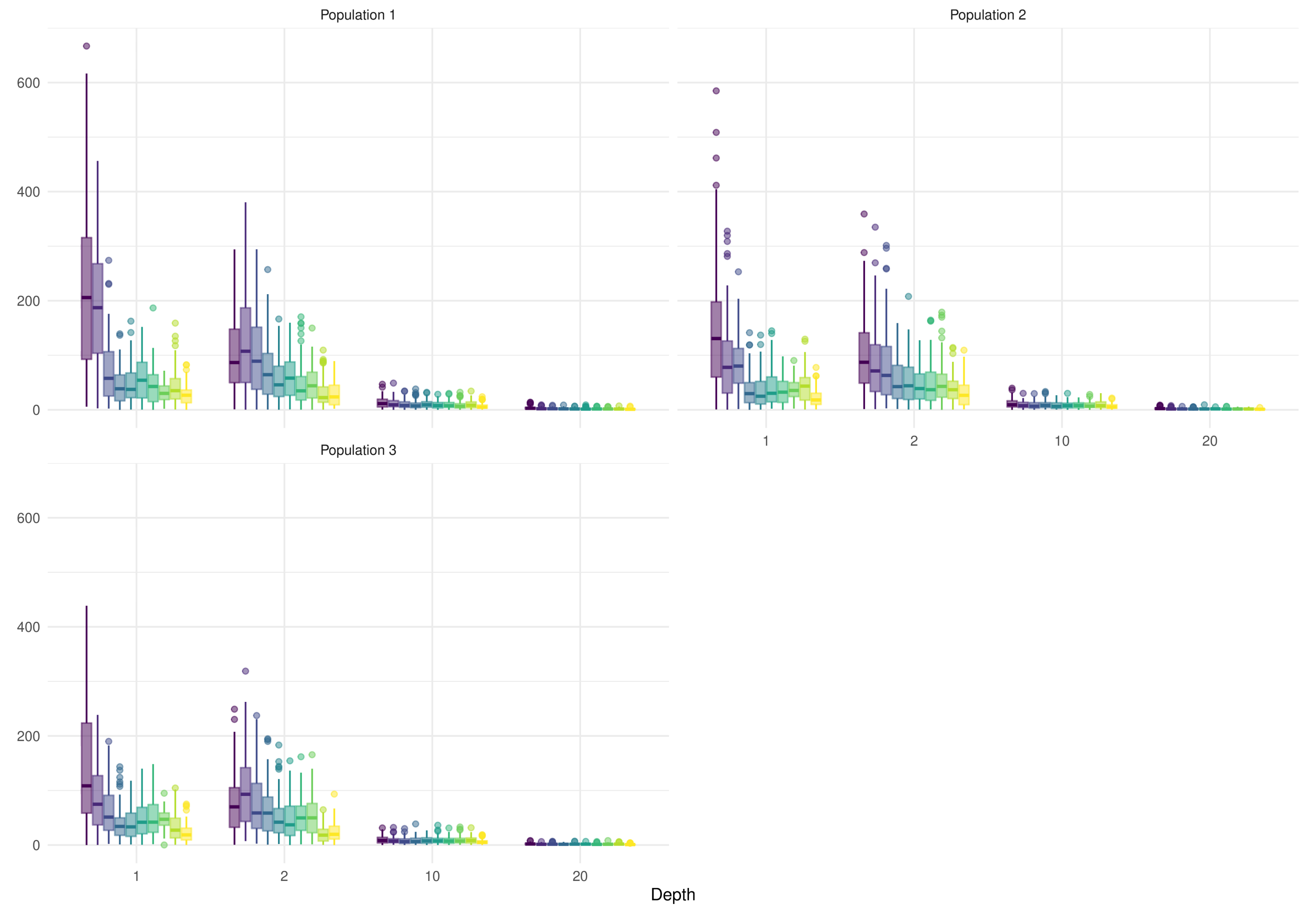

Fig S 15. Root mean squared deviation of the population-based folded SFS. Each panel represents the root mean squared deviation of the folded SFS for a different population. Each of the coloured distributions of the box plot shows the values for root mean squared deviation of the log_{10} of the number of each allele frequency.

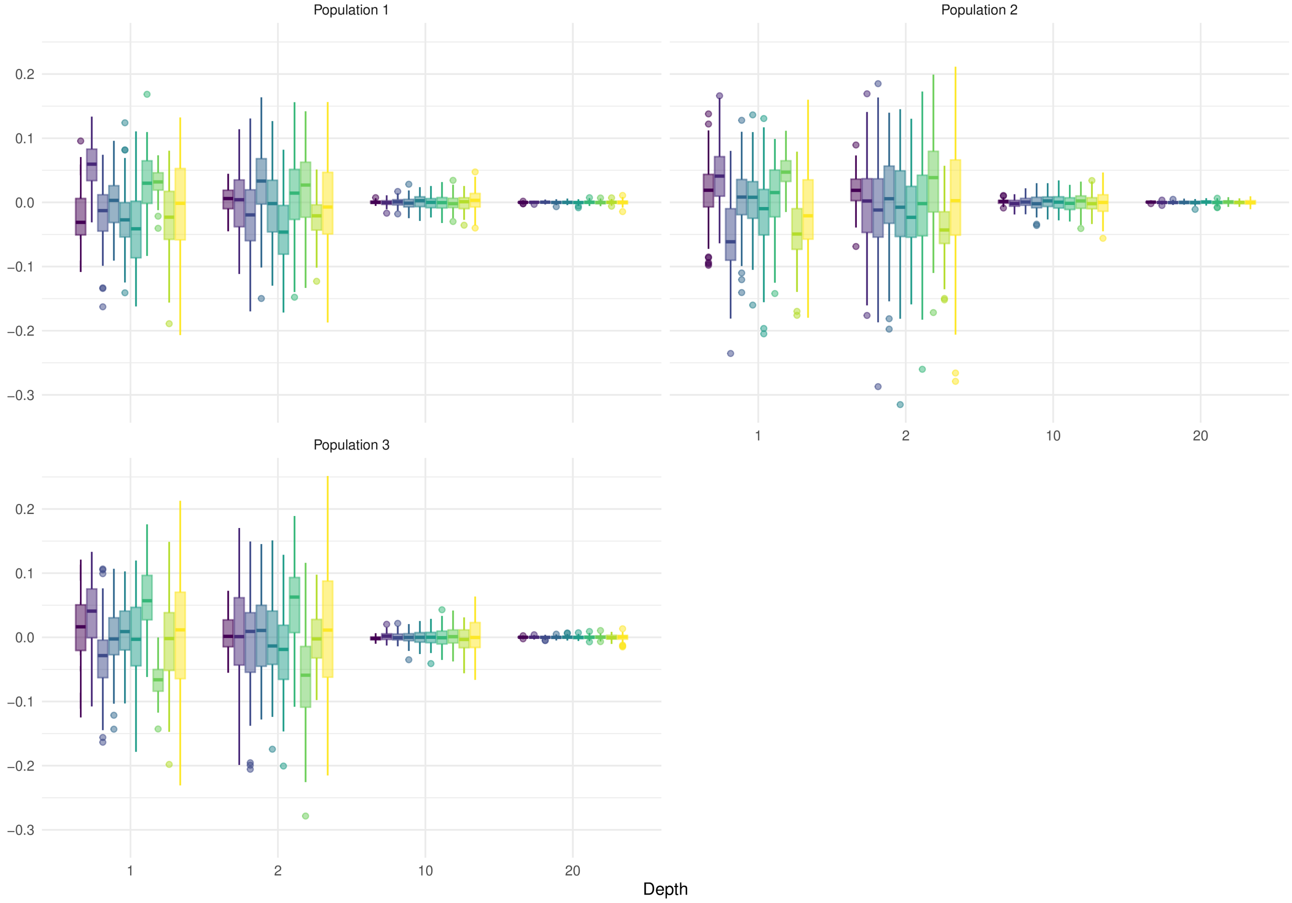

Fig S 16. Standardised bias of the population-based folded SFS. Each panel represents the standardised bias of the folded SFS for a different population. Each of the coloured distributions of the box plot shows the values for the standardised bias of the log_{10} of the number of each allele frequency.

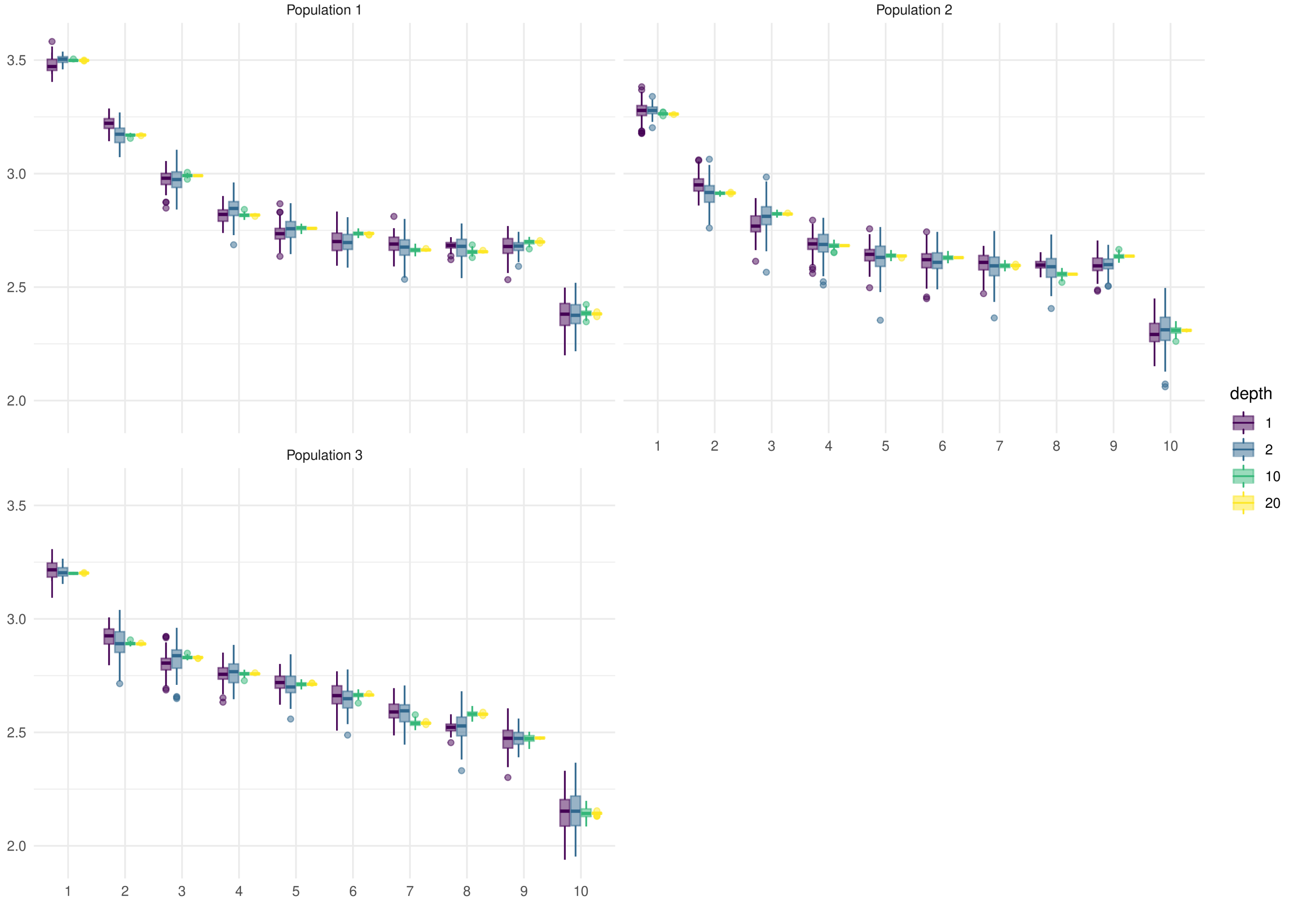

Fig S 17. Popualtion-based folded SFS. Each panel represents the folded SFS for a different population, for each allele frequency. Each of the coloured distributions of the box plot shows the log_{10} of the number of occurrences in depths 1x, 2x, 10x and 20x.

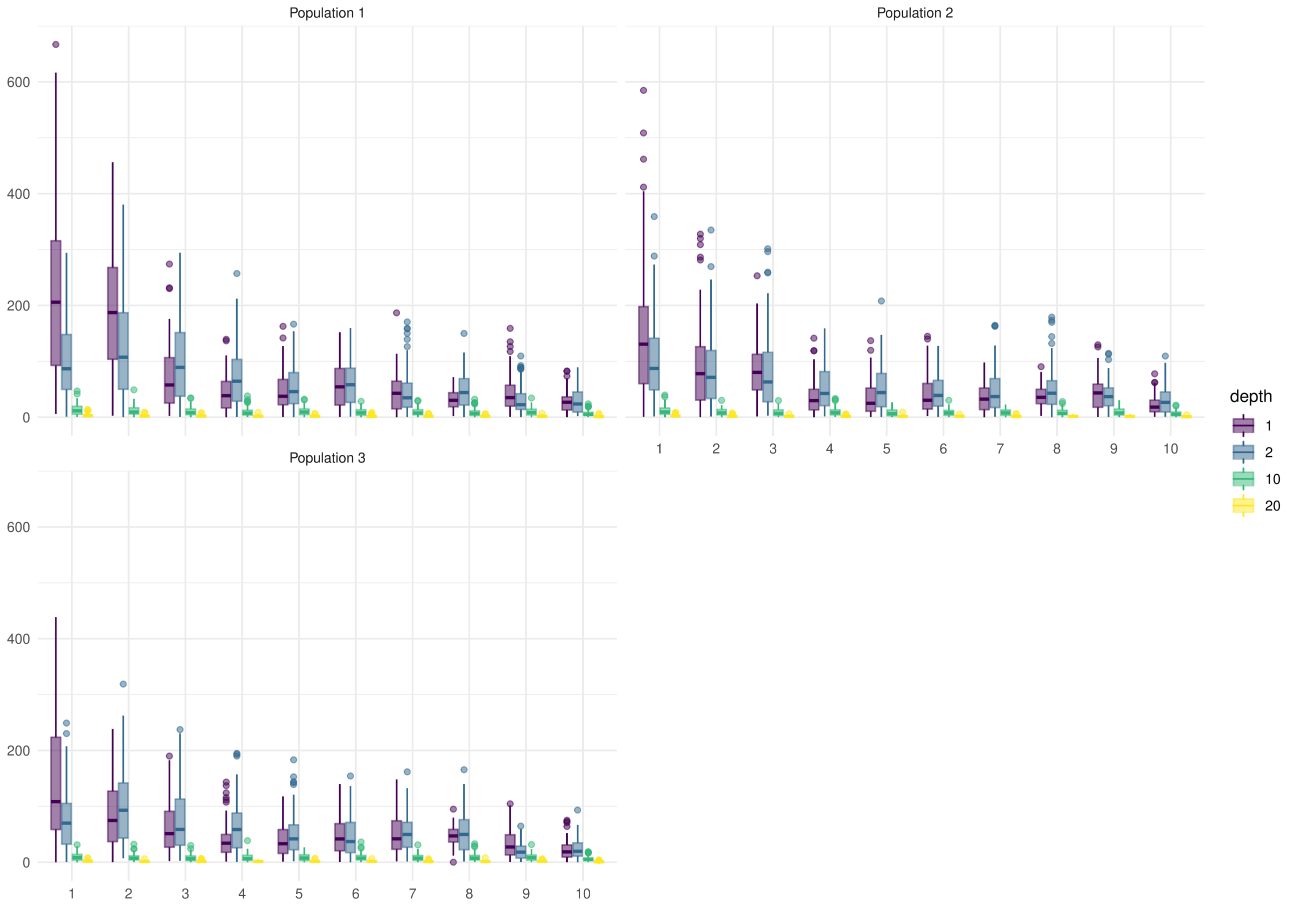

Fig S 18. Root mean squared deviation of population-based folded SFS values shown at Fig S17. Each panel represents the standardised bias of the folded SFS for a different population, for each allele frequency. Each of the coloured distributions of the box plot shows the standardised bias of the log_{10} of the number of occurrences in depths 1x, 2x, 10x and 20x.

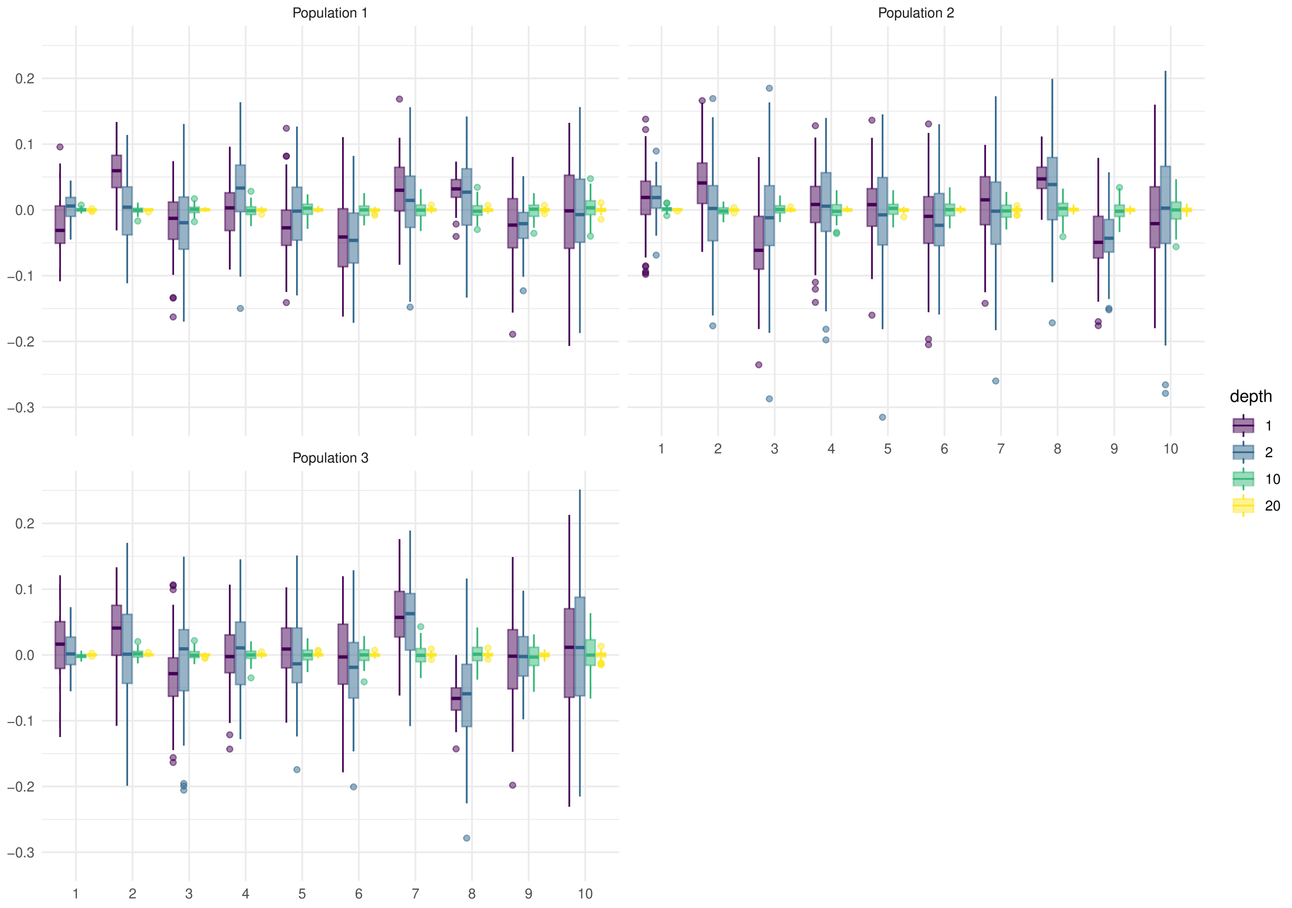

Fig S 19. Standardised bias of population-based folded SFS values shown at Fig S17. Each panel represents the standardised bias of the folded SFS for a different population, for each allele frequency. Each of the coloured distributions of the box plot shows the standardised bias of the log_{10} of the number of occurrences in depths 1x, 2x, 10x and 20x.

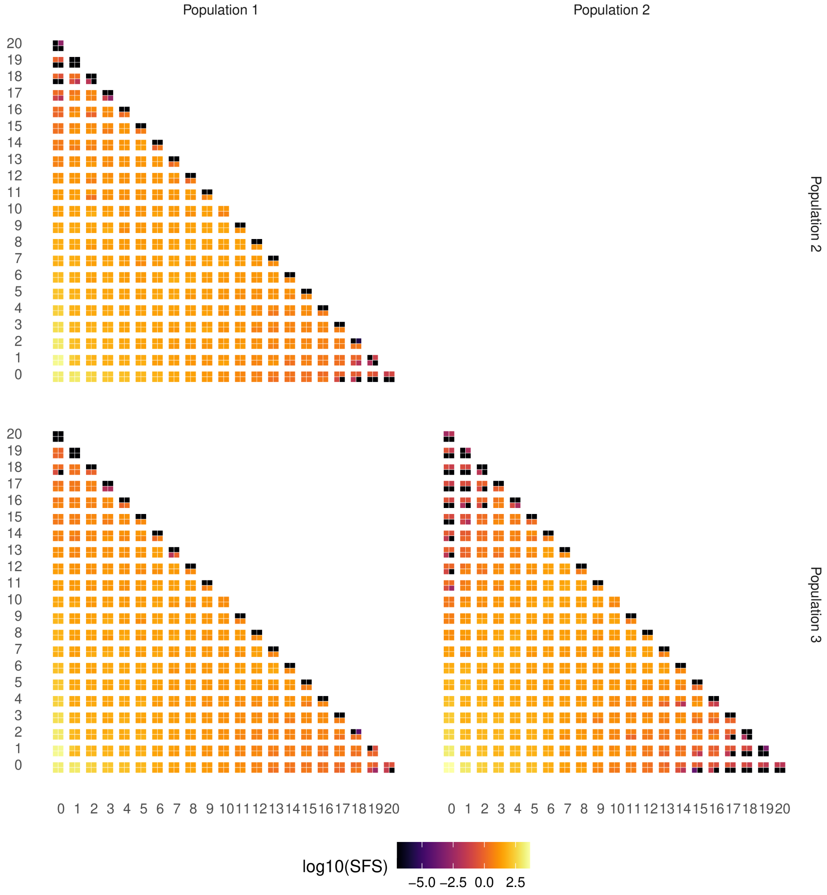

Fig S 20. Two-population joint folded SFS. Each panel represents the two-population joint folded SFS for a different pair of populations, for each allele frequency. Each color of the four-tile squares is according to the log_{10} of the number of occurrences at depths 1x, 2x, 10x and 20x, like in Fig 2.

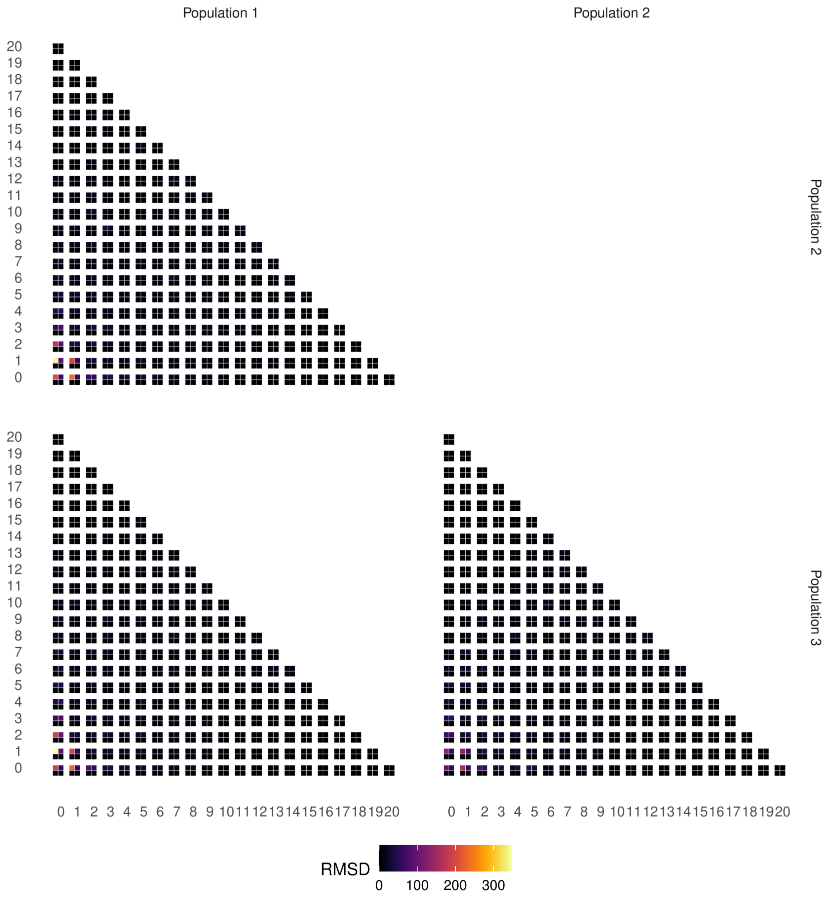

Fig S 21. Root mean squared deviation of the Two-population joint folded SFS values shown at Fig S20. Each panel represents the root mean squared deviation of the two-population Joint SFS for a different pair of populations, for each allele frequency. Each color of the four-tile squares is according to the root mean squared deviation of the log_{10} of the number of occurrences at depths 1x, 2x, 10x and 20x, like in Fig 2.

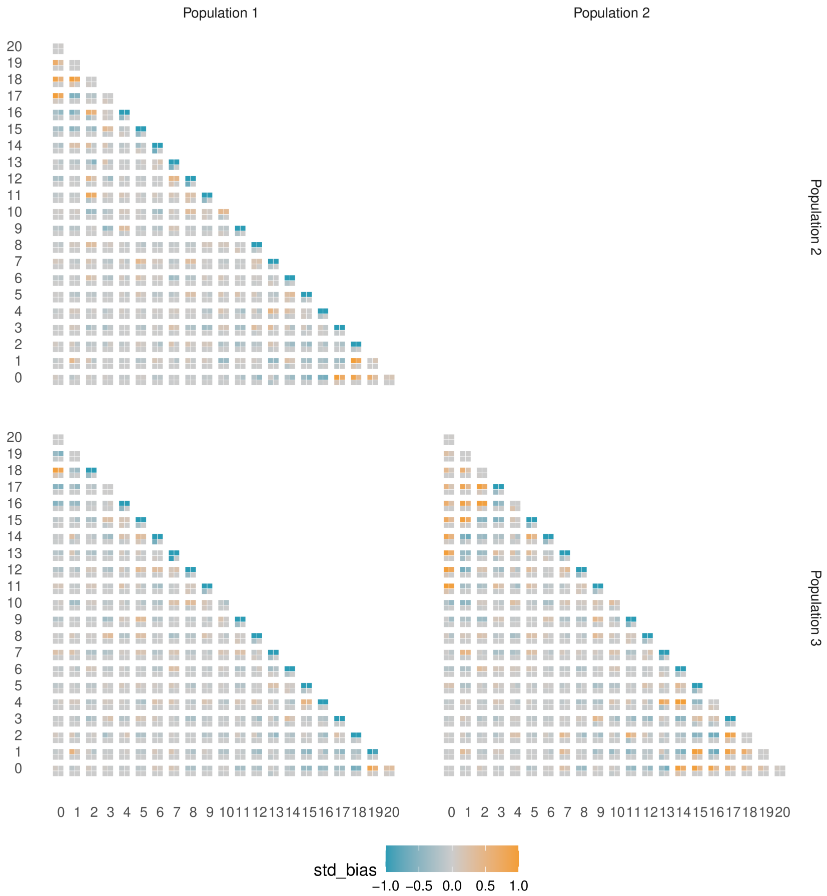

Fig S 22. Standardised bias of the Two-population joint folded SFS values shown at Fig S20. Each panel represents the standardised bias of the two-population joint SFS for a different pair of populations, for each allele frequency. Each color of the four-tile squares is according to the standardised bias of the log_{10} of the number of occurrences at depths 1x, 2x, 10x and 20x, like in Fig 2.

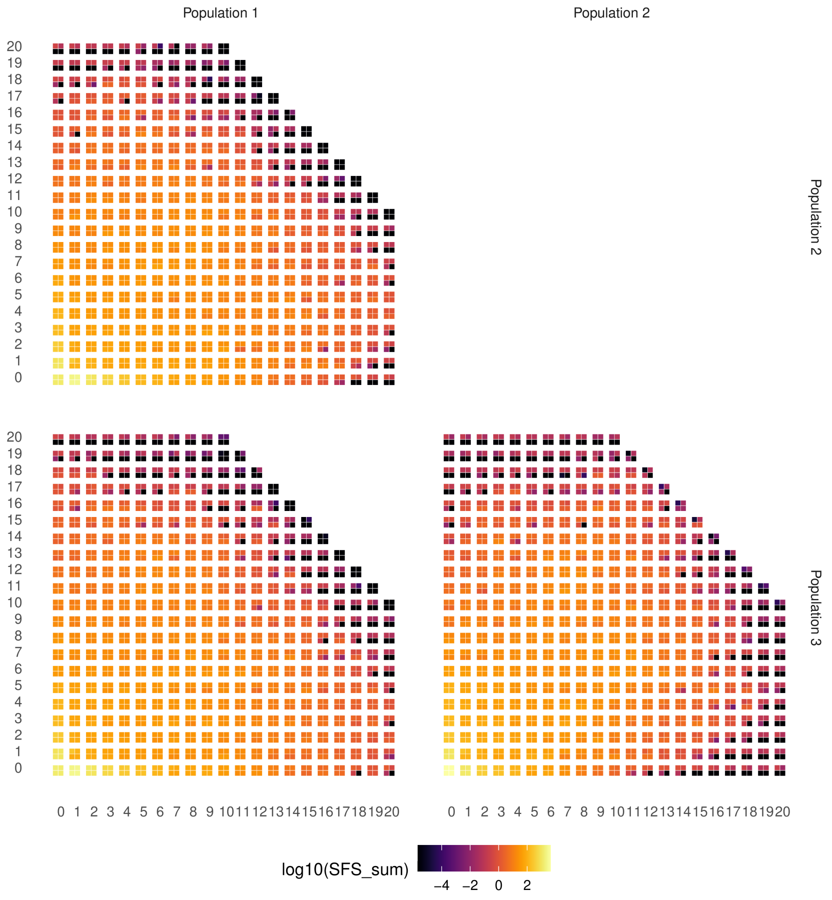

Fig S 23. Three-population joint folded SFS. Each panel represents the three-population joint folded SFS for a different pair of populations, for each allele frequency. Each color of the four-tile squares is according to the log_{10} of the **marginal sum** of the number of occurrences across the third axis at depths 1x, 2x, 10x and 20x, like in Fig 2.

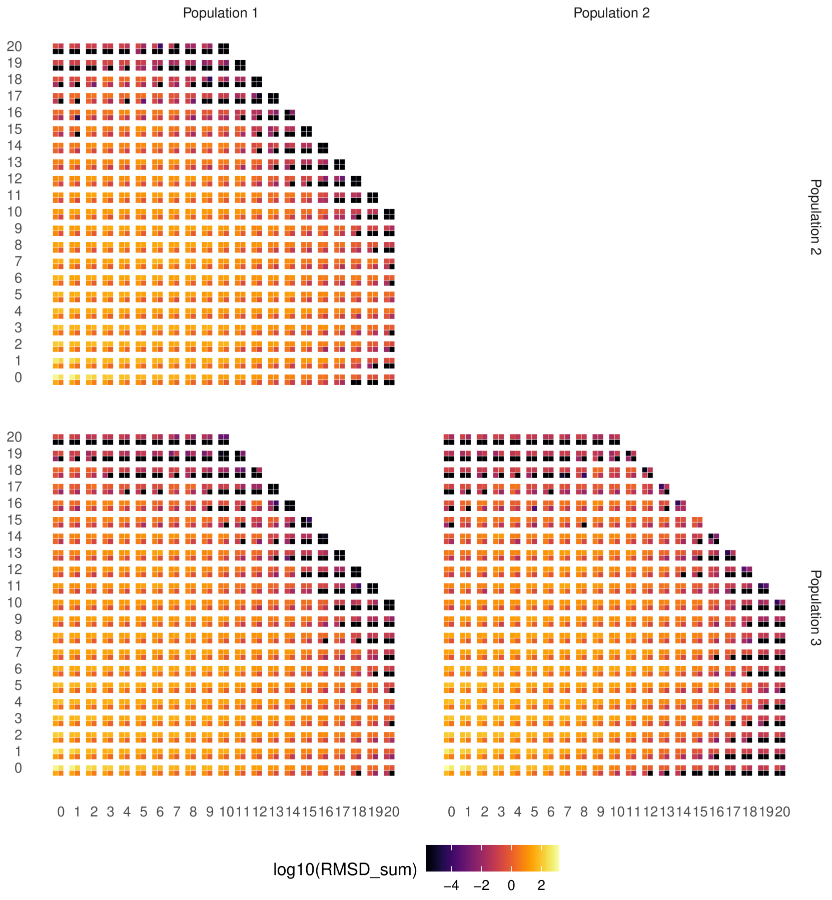

Fig S 24. Root mean squared deviation of the three-population joint folded SFS shown at Fig S23. Each panel represents the root mean squared deviation of the three-population joint SFS for a different pair of populations, for each allele frequency. Each color of the four-tile squares is according to the root mean squared deviation of the log_{10} of the **marginal sum** of the number of occurrences across the third axis at depths 1x, 2x, 10x and 20x, like in Fig 2.

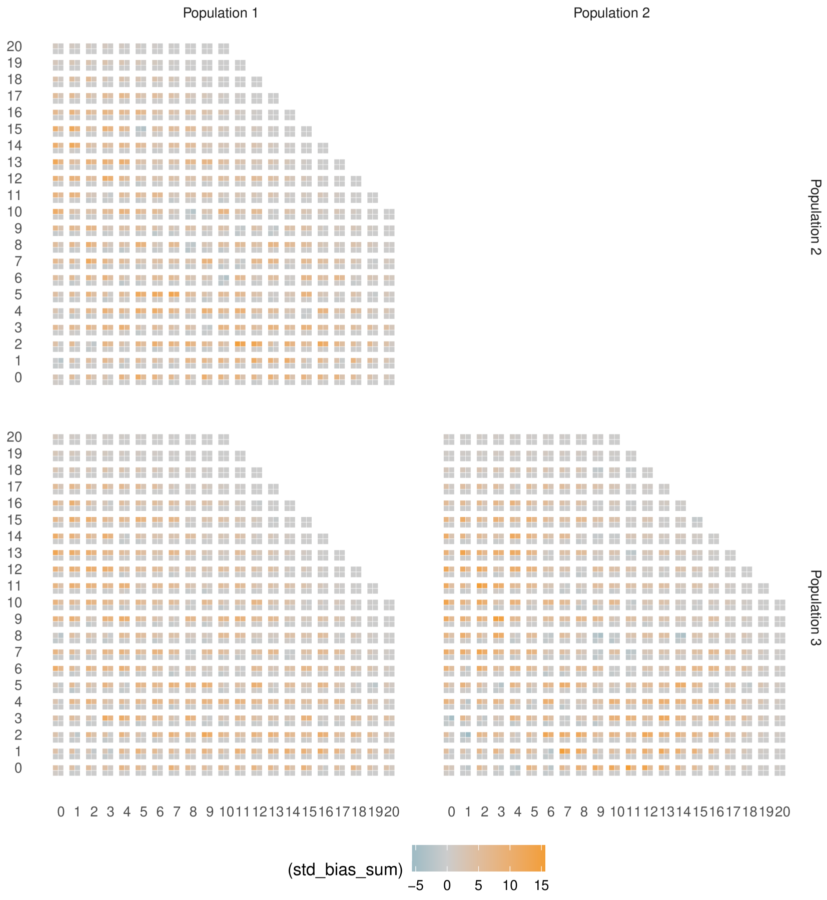

Fig S 25. Standardised bias of three-population joint folded SFS shown at Fig S20. Each panel represents the standardised bias of the three-population joint SFS for a different pair of populations, for each derived allele frequency. Each color of the four-tile squares is according to the standardised bias of the log_{10} of the **marginal sum** of the number of occurrences across the third axis at depths 1x, 2x, 10x and 20x, like in Fig 2.

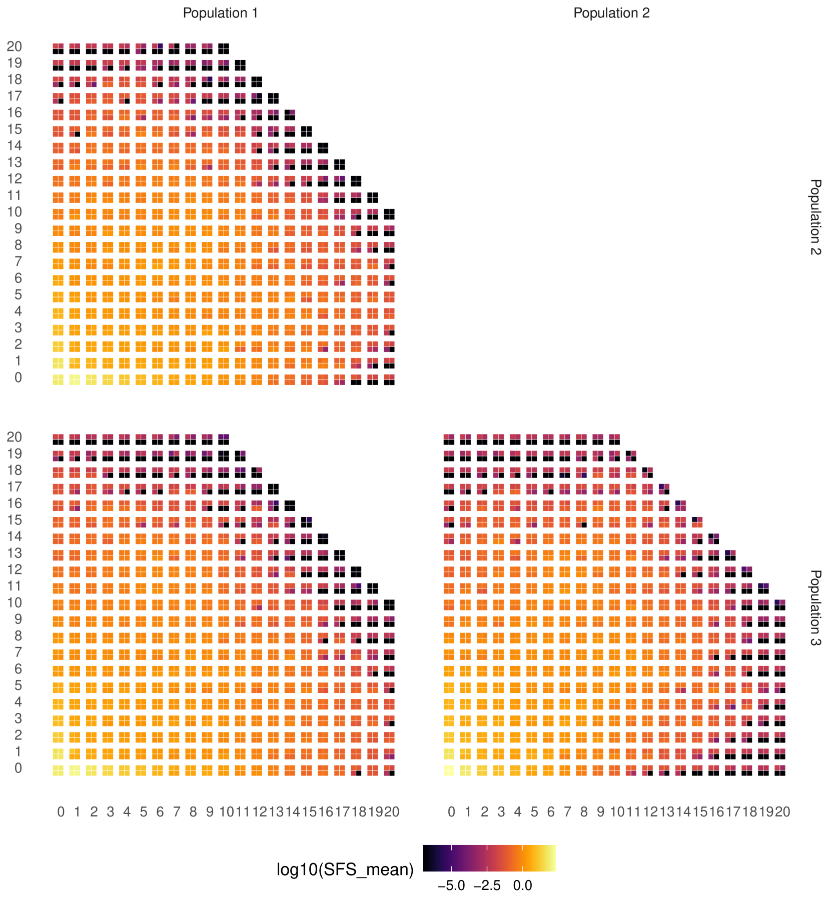

Fig S 26. Three-population joint SFS. Each panel represents the three-population joint folded SFS for a different pair of populations, for each derived allele frequency. Each color of the four-tile squares is according to the log_{10} of the **mean** of the number of occurrences across the third axis at depths 1x, 2x, 10x and 20x, like in Fig 2.

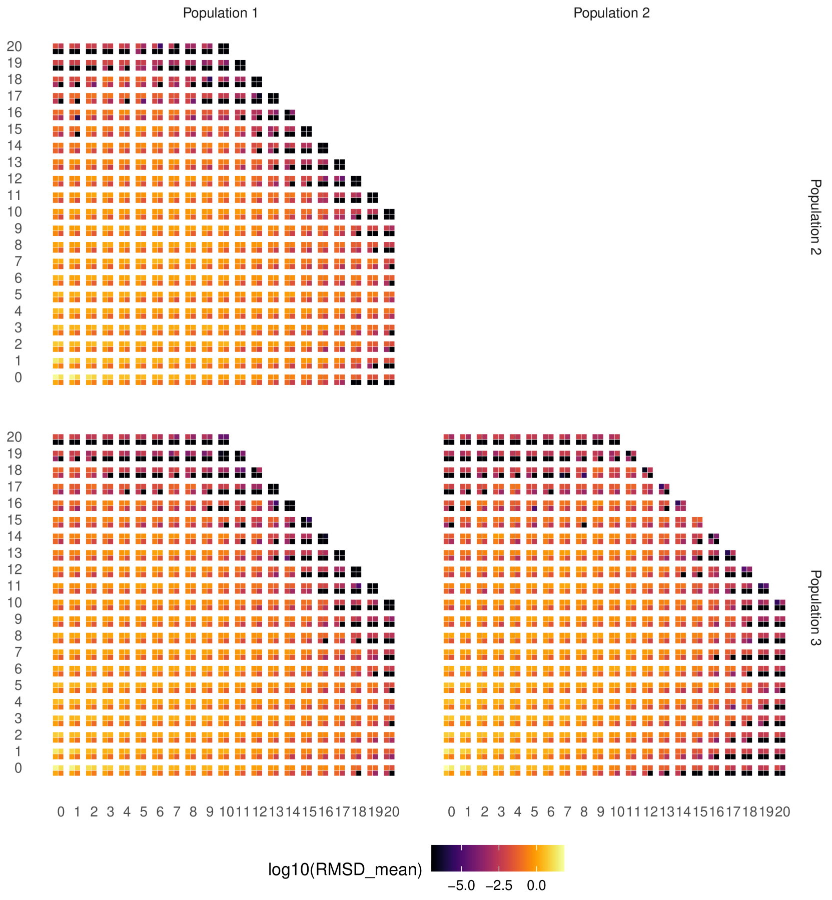

Fig S 27. Root mean squared deviation of the three-population joint SFS shown at Fig S11. Each panel represents the root mean squared deviation of the three-population joint SFS for a different pair of populations, for each derived allele frequency. Each color of the four-tile squares is according to the root mean squared deviation of the log_{10} of the **mean** of the number of occurrences across the third axis at depths 1x, 2x, 10x and 20x, like in Fig 2.

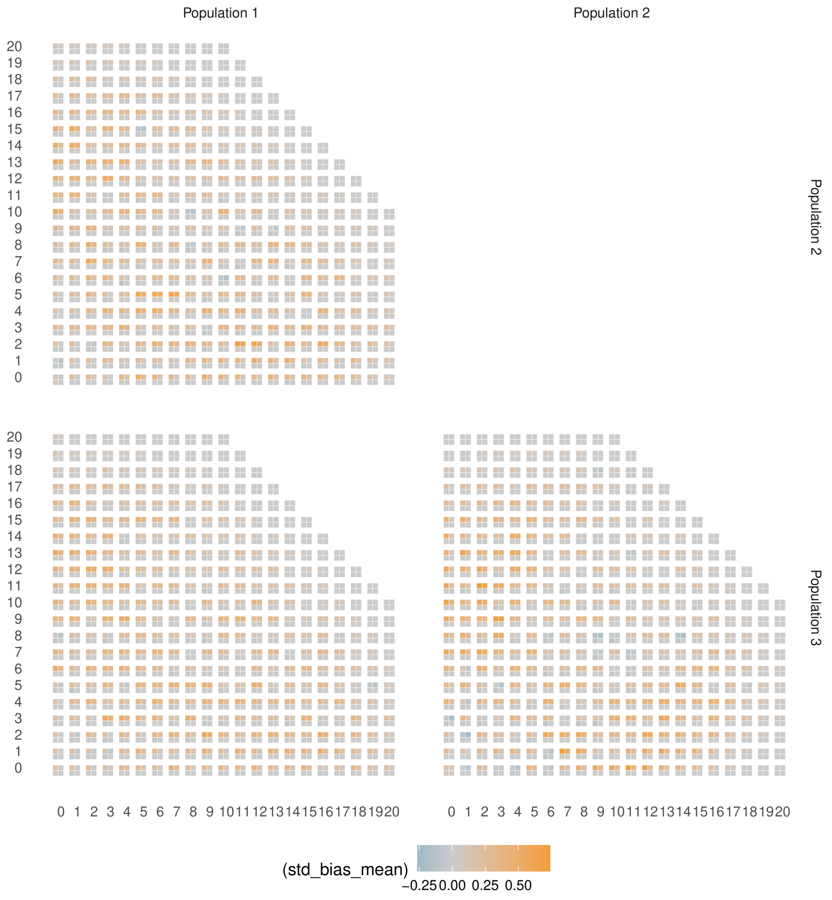

Fig S 28. Standardised bias of three-population joint SFS shown at Fig S11. Each panel represents the standardised bias of the three-population joint SFS for a different pair of populations, for each derived allele frequency. Each color of the four-tile squares is according to the standardised bias of the log_{10} of the **mean** of the number of occurrences across the third axis at depths 1x, 2x, 10x and 20x, like in Fig 2.

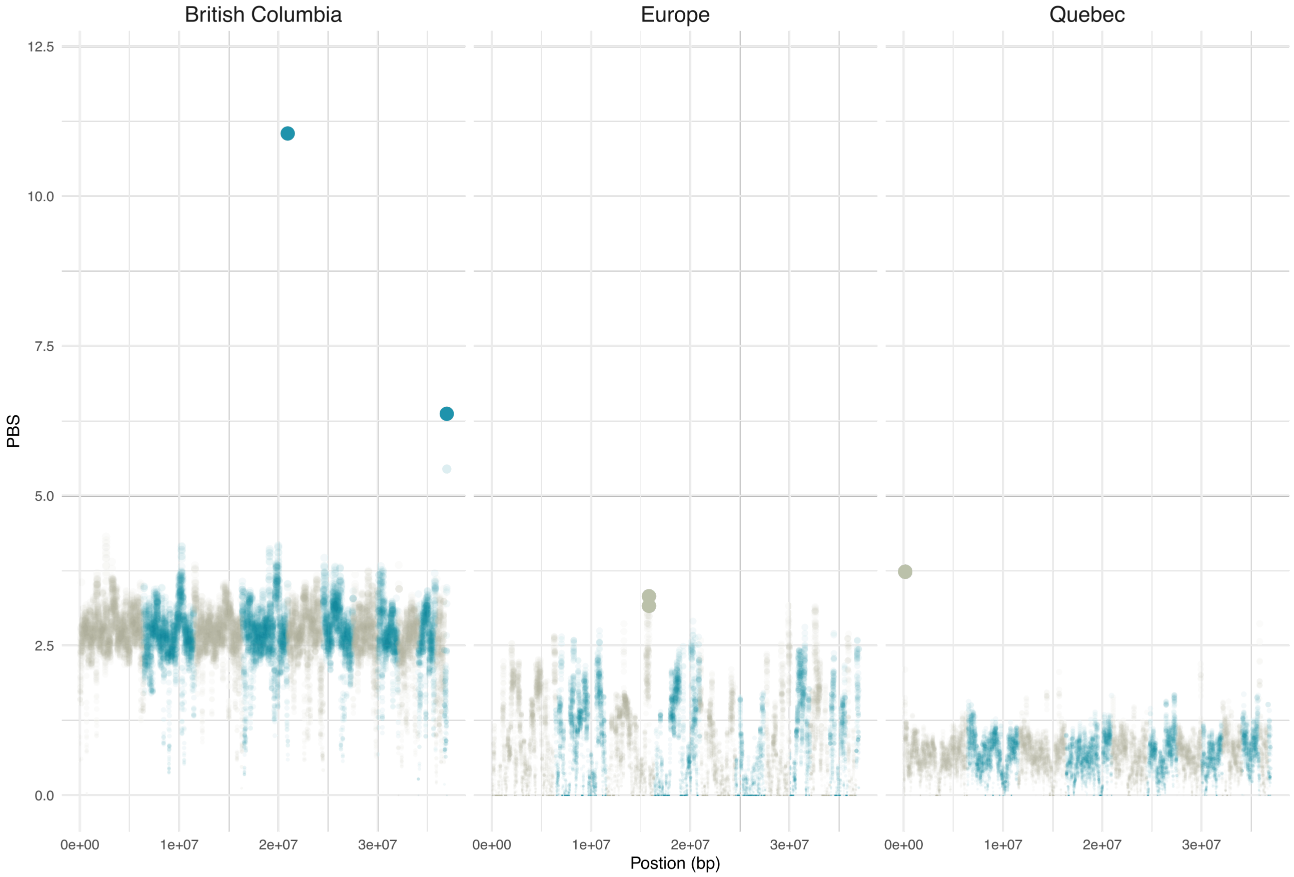

Fig S 29. Sliding windows scan for genetic differentiation in three populations of *Neonectria neomacrospora*. We calculated *PBS* in sliding windows of 20kbp with a step of 2kbp. Each panel represents a separate scan where each population was considered the target and the remaining two controls. The highlighted points indicate windows with an empirical rank p-value lower than 10^{-3} in each population. Compared to Figure 3, sequencing data is not downsampled.
