# Supplementary material for: Fast and accurate estimation of multidimensional site frequency spectra from low-coverage high-throughput sequencing data
Source: Gigascience. 2022 May 17;11:giac032. doi: 10.1093/gigascience/giac032 (PMC9112775; doi:10.1093/gigascience/giac032)
Supplement: giac032_Response_to_Reviewer_Comments_Original_Submission [file giac032_response_to_reviewer_comments_original_submission.pdf]

Author's Response To Reviewer Comments

Close

Dear editor, we thank the reviewers for their input and we have responded to all their comments below point-by-point. We have updated the manuscript and the supplementary information to resolve the comments and issues that were raised by the reviewers. We believe that the comments from reviewers have improved the manuscript and we thank them.

Best regards
Thorfinn Sand Korneliussen

GIGA-D-21-00341 answer to reviewer
Fast and accurate estimation of multidimensional site frequency spectra from low-coverage high-throughput sequencing data
Alex Mas-Sandoval; Nathaniel S. Pope; Knud Nor Nielsen; Isin Altinkaya; Matteo Fumagalli; Thorfinn Sand Korneliussen
GigaScience

Dear Dr Korneliussen,

Your manuscript "Fast and accurate estimation of multidimensional site frequency spectra from low-coverage high-throughput sequencing data" (GIGA-D-21-00341) has been assessed by our reviewers. Their reports are below. The reviewers are positive overall, but they also mention a couple of points that need to be clarified, and other suggestions for improvements.

Once you have made the necessary corrections, please submit a revised manuscript online at:

https://www.editorialmanager.com/giga/

In addition, please register any new software application in the bio.tools and SciCrunch.org databases to receive RRID (Research Resource Identification Initiative ID) and biotoolsID identifiers, and include these in your manuscript (in the "availability" section). This will facilitate tracking, reproducibility and re-use of your tool.

We have now obtained the RRID: SCR_021865 and have added this information to the availability section of the manuscript.

If you have forgotten your username or password please use the "Send Login Details" link to get your login information. For security reasons, your password will be reset.

Please include a point-by-point within the 'Response to Reviewers' box in the submission system. Please ensure you describe additional experiments that were carried out and include a detailed rebuttal of any criticisms or requested revisions that you disagreed with. Please also ensure that your revised manuscript conforms to the journal style, which can be found in the Instructions for Authors on the journal homepage. If the data and code has been modified in the revision process please be sure to update the public versions of this too.

The due date for submitting the revised version of your article is 06 Mar 2022.

We look forward to receiving your revised manuscript soon.

Best wishes,

Hans Zauner

GigaScience
www.gigasciencejournal.com

Reviewer reports:
Reviewer #1: GIGA-D-21-00341

review report

The authors introduce a new method for estimating the site-frequency spectrum from
low-coverage high-throughput DNA sequence data. This is important since it saves on the cost of sequencing,
enabling researchers to direct resources towards obtaining more samples.

The work is well motivated and well written, and the difference in memory usage is impressive.

My main comment (see below) concern the presentation of the arguments for Eq 1; and on providing additional
background on genotype likelihoods. If you run into space restrictions for the main text, then I assume you could provide this in Supporting Information.

We agree and have expanded the manuscript see 2) and 3) for details.

The comments aside, the methods are appropriate to the aims of the study, could be better described (see my comments),
necessary controls are included, and conclusions are adequately supported.

Comments:

0: I think for reviewing purposes it would have been better to format the manuscript into single-column mode
with line numbers; or maybe it is possible to include line numbers in two-column mode ?

We agree that this would be useful for peer reviewing purposes and hope that the editor will consider this for future manuscripts.

1: An over-representation of rare alleles (e.g. singletons) can be an indicator of many things, not just population expansion; and repeated strong bottlenecks can be shown to generate gene genealogies looking
like multiple-merger genealogies, which generally predict a U-shaped SFS, i.e. an excess of low and high-frequency
variants

We agree with the reviewer, this is an important point. We have now added the sentence: "Complex scenarios of repeated bottlenecks and gene flow may also generate an excess of rare alleles (Eldon et al. 2015 Genetics; Marchi and Excoffier. 2020 Evolutionary Applications.)"

2: I think it would be good to rewrite the paragraph around Eq 1. The vector y needs to be better explained,
i.e. is y[i] is the likelihood of seeing i derived alleles in a haploid sample of size n ? Can I interpret a 'sample' as
one chromosome, for which you have x number of reads ? I am also confused about the notation : if y[i] is the ith coordinate
of y, where does y^{(1)} fit in? And \phi(i,j) in Eq 1 is a component of y, the jth component maybe ? You also need
to give some arguments how you obtain Eq 1.

"y" is defined and explained for diploids in the previous paragraph. We edited the first sentence of the paragraph in question to make the translation to haploids clearer:

"We have also developed an analogous algorithm for haploids, in which case y has $n+1$ elements and $y[i]$ is the likelihood of i derived alleles in a sample of n haploids."

We also clarify the meaning of the superscript in a parenthetical statement,

“the superscript (i) indicates that the vector incorporates genotype likelihoods from i samples and thus has $i+1$ elements”

We clarify the relationship between $\phi(i,j)$ and $y^{(i)}[j]$ directly in Eq 1.

Finally, we include the derivation of Eq 1 from the underlying probability model in the supplementary information.

3: Please include background on genotype likelihoods, and how they are defined and calculated. If I remember correctly there
is an equation for the likelihood in the original paper on genotype likelihoods. This equation should be included here and
explained. I am aware the current manuscript is submitted as a technical note, and seems directed towards those using
these methods on a daily basis, but there should still be room for additional background and clarifications.

We agree with the reviewer that the manuscript would benefit from a section regarding how genotype likelihoods are calculated and we have added the following paragraph.

" In general the genotype likelihoods are calculated independently for each individual for each site and is the probability of the read data given the true unobserved genotype, which in a diploid context is given by
$$L(G=\{A_1,A_2\}\mid D) \propto Pr(D\mid G=\{A_1,A_2\}),\qquad A_1,A_2 \in \{A,C,G,T\}$$
Many genotype likelihood models exists \cite{samtools,gatk,atlas,soapsnp} and the canonical genotype likelihood model is shown below, M denotes sequencing depth and e being the error rate which is in practice given by the phred scaled base quality score of the read:
$$Pr(D\mid G=A_1A_2)=\prod_{i=1}^MPr(b_i\mid G=A_1A_2)=\prod_{i=1}^M\left (p(b_i\mid A_1)+p(b_i\mid A_2)\right)$$
$$p(b\mid A) = \begin{cases}
\frac{e}{3} & b \neq A \\
1-e & b = A
\end{cases}$$
"

4: by a 'genomic region' you are referring to a segment of a chromosome ?

Yes, we have expanded the sentence to "large genomic region such as a segment of a chromosome".

5: Having the algorithms is nice, but I think it would be good if you explained in words how you generalized the EM-algorithm in
Ref 5

The folded model is optimized by introducing a binary latent variable per site that indicates if the allele is correctly or incorrectly polarized (in addition to the per-site latent variables in the “unfolded” version of the algorithm). The usual EM recipe is then applied (find the conditional expectation of the full likelihood wrt the latent variables, find the values of the folded SFS that maximizes this conditional expected likelihood, rinse and repeat). The mentioned reference discusses the particular case of estimating the folded SFS of a single population & uses quasi-Newton methods to optimize; whereas we derive an EM update for the joint folded SFS of an arbitrary number of populations, that also uses the sparse banded per-site likelihoods. We’ve added a sentence to this effect,

“To this end, we generalized the single-population probability model for the folded SFS in \cite{nielsen2012snp} to an arbitrary number of populations and derived an EM update for efficient optimization (Algorithm \ref{emAlgo}). Briefly, this is accomplished by introducing per-site latent variables that indicate the number of non-ancestral alleles in the sample and whether the site is correctly polarized, then taking the expectation of the joint log probability function with regard to these latent variables to find the EM update \cite{mclachlan2007algorithm,korneliussen2014angsd}”

Finally, we included the derivation of the EM update in the supplementary information.

6: thank you for providing the command line for the program ms, but for those without detailed knowledge of the input for ms, maybe
provide a description of the input values you used ?

We have expanded on this in full details and have included the following sentence "The `ms` command produced 1 replicate of 60 sampled haplotypes (ms 60 1) for 3 populations with equal sample size (-I 3 20 20 20) with fixed mutation and recombination rates (-t 1935 -r 2167) scaled by the region length (5130456 bp). These values correspond to realistic average values of mutation (Scally 2016) and recombination rates (Myers et al 2005) in the human genome. The three populations experienced changes in effective sizes (switches -n, -eg and -en), gene flow (-ma and -ema), and splits (-ej) following a previously proposed demographic model (Gutenkunst et al 2009). "

7: do you have references for the 'realistic' values of the recombination and mutation rates you used ?

We have expanded on this and now include the sentence: "These values correspond to realistic average values of mutation (Scally 2016) and recombination rates (Myers et al 2005) in the human genome. "

8: the program ms has been around for a while, and used by many, but there is a new kid in town called msprime, which is not only
orders of magnitude more efficient than ms for common tasks, but is also thoroughly tested. So I was wondering if you would
be willing to redo your analysis in msprime, and so provide a msprime-based pipeline for your analysis. I think from now on people will
be using msprime, so for reproducibility I think this might be helpful.

We have now included a sentence on how simulations can be run using msprime: "Simulations can be run using `msprime` \cite{Baumdicker2021} with the program `mspms` which allows to replace `ms` commands."

9: are there estimates of the error rate in these new sequencing methods ? I was wondering if your 1% sequencing error rate was
somewhere near real values.

The error rates vary from 0.087% in HiSeq X Ten to 0.613% in MiniSeq. We choose 1% to show the performance in unrealistic high error rates scenarios. See this publication for background information and reference regarding error rates for different platforms. (NAR Genomics and Bioinformatics, Volume 3, Issue 1, March 2021, lqab019, https://doi.org/10.1093/nargab/lqab019). We added the following sentence to the manuscript "We choose an unrealistic high error rate of 1% to show the performance of our method in a worst case setting \cite{illuminaError}"

10: How did you simulate the reads of the simulated data?

We agree with the reviewer and this needed to be clarified. We have now added the following sentence to the manuscript. ", for details regarding the simulation algorithm we refer to \cite{korneliussen2013calculation}"

11: the site-frequency spectrum (SFS), by definition, is 'multidimensional' since it is a vector ? Or, framed another way, if you are
estimating the joint SFS for two or more populations, where (X_i, X_j) would be the (i,j) coordinate and X_i the count of sites
carrying i derived alleles in population 1, and X_j similar for population 2, I would call it the 'joint' SFS

We have added the word "joint" to the abstract so it is clear "Here we design and implement a method to efficiently and accurately estimate the multidimensional joint site frequency spectrum for large numbers of haploid or diploid individuals across an arbitrary number of populations, using low-coverage sequencing data."

12: correct me if I am wrong, but don't you need at least 2x coverage to compute genotype likelihoods ?

We have written a complete paragraph regarding genotype likelihoods, this include definition, references for different models and an example of how there are computed. We agree that this part was vague previously. See also answer to reviwer 3).

Reviewer #2: The authors present an implementation of a method for efficiently inferring the multidimensional site frequency spectrum from low-coverage genome data. The implementation is an update to ANGSD, a widely-used software package for manipulating genomic data.

Overall, the improved tool is likely to be of interest to those in the population genomics community, particularly because many applications will no longer require server-sized amounts of memory. The paper is generally clear, with exceptions noted below.

Major points:
1) The main text figures are absolutely illegible at printed size. They need to be carefully reworked with larger fonts and points.

We resized the size of the figures and the font size. For figure 1 we changed both the font size and the display of the four panels. For figure 2, the font size is accurate, but it should be shown with a text width, not column width. For figure 3 we have increased the font size.

2) I am confused by the benchmark in Table 1. The Methods section implies that the resulting spectrum is 1-pop ("assuming they are from a single population"), but the results seciton describes "estimating the multidimensional SFS". Which is it?

Yes this is an error, we have changed the text to: "We first compared the computational performance between the original and new (labeledf 'banded') implementation for estimating the site frequency spectrum at different sample sizes.

3) I am confused by the differences between Fig 2 and Fig S6. In principle, the 2-population spectra obtained from marginalizing the 3-pop spectrum should be identical to the 2-pop spectra obtained directly from the data. Can the authors comment on the differences between these approaches when their algorithm is applied?

If errors and information are not identical between the populations we should not expect the marginal to be the same. Assuming two populations, one with extremely low error the other with extreme high error, then the marginalized will be affected by the noisy joint distribution and will not be the same as the direct.

Minor points:
1) Results, Computational Performance: The phrase "no longer exhibits proportional increase of computational requirements with sample size" seems incorrect. The data show that for both the orginal and banded methods, the time increases with sample size. Do the authors mean "memory requirements"?
Yes we agree with reviewer. We have now changed the sentence to: " which no longer exhibits a proportional increase of memory with sample size."

2) Results, Estimation of frequency spectra: The claim is made that "mean estimates... do not differ". This is not quite true for the multipopulation spectra, where there is a systematic bias upward for low-coverage estimates of sfs cells which contain little to no data. This is acknowledged later in the section, but I would ask the authors to be more explicit. (I suspect such biases are unlikely to affect downstream analyses substantially, since they affect only a small fraction of the total weight in the SFS.)

We have modified the sentence to “We observe that the mean estimates of SFS across replicates do not differ between sequencing depths except for few private and low frequency bins, where lower depths tend to overestimate”

3) Figure 1 caption: log_{10} instead of "log_10 scale".
Fixed

--
Please also take a moment to check our website at https://www.editorialmanager.com/giga/l.asp?i=101996&l=HNBF21OR for any additional comments that were saved as attachments. Please note that as GigaScience has a policy of open peer review, you will be able to see the names of the reviewers.

____
In compliance with data protection regulations, you may request that we remove your personal registration details at any time. (Use the following URL: https://www.editorialmanager.com/giga/login.asp?a=r). Please contact the publication office if you have any questions.

Close
