# Supplementary material for: Fast and accurate estimation of multidimensional site frequency spectra from low-coverage high-throughput sequencing data
Source: Gigascience. 2022 May 17;11:giac032. doi: 10.1093/gigascience/giac032 (PMC9112775; doi:10.1093/gigascience/giac032)
Supplement: giac032_Response_to_Reviewer_Comments_Revision_1 [file giac032_response_to_reviewer_comments_revision_1.pdf]

Author's Response To Reviewer Comments

Close

Dear Editor and reviewers. We added the RRID in last resubmission and we have answered to the last two comments from reviewer1 below:

1) we have now expanded the paragraph with the explanation and information of genotype likelihoods:

"Many genotype likelihood models exists \cite{samtools,gatk,atlas,soapsnp} and the canonical genotype likelihood model is shown below, M denotes sequencing depth, b_i is the nucleotide for the ith read and e_i is the associated error rate which is in practice given by the phred scaled base quality score of the nucleotides of the read:"

2) We have now expanded the paragraph:

"Given a sample of n haploids with sequence data D_j. Here D_j denotes all the information that goes into the calculation of the genotype likelihoods. This includes the sequenced bases, quality scores, position within the reads but possibly also the mapping quality and external information such as library preparation type. $G_j \in \{0,1\}$ are the (unknown) genotypesfor the jth haploid, the likelihood that the number of derived alleles in the sample equals i is,"

Best
Thorfinn

GIGA-D-21-00341R1
Fast and accurate estimation of multidimensional site frequency spectra from low-coverage high-throughput sequencing data
Alex Mas-Sandoval; Nathaniel S. Pope; Knud Nor Nielsen; Isin Altinkaya; Matteo Fumagalli; Thorfinn Sand Korneliussen
GigaScience

Dear Dr Korneliussen,

Your revised manuscript "Fast and accurate estimation of multidimensional site frequency spectra from low-coverage high-throughput sequencing data" (GIGA-D-21-00341R1) has been assessed by our reviewers. I am pleased to inform you that it is acceptable for publication in GigaScience, in principle.

Before we proceed, please address the minor, final reviewer's comments below.
Our data curators will also contact you shortly to prepare the supporting data for GigaDB. Please include the GigaDB citation (including doi), which you will get from our curators, in the bibliography and cite it by number from the "availability" section.

In addition, please register any new software application in the bio.tools and SciCrunch.org databases to receive RRID (Research Resource Identification Initiative ID) and biotoolsID identifiers, and include these in your manuscript. This will facilitate tracking, reproducibility and re-use of your tool. (If the ANGSD platform already has RRID and biotool accessions, you can also include this).

Once you have made the necessary corrections, please submit a revised manuscript online at:

https://www.editorialmanager.com/giga/

If you have forgotten your username or password please use the "Send Login Details" link to get your login information. For security reasons, your password will be reset.

Please include a point-by-point within the 'Response to Reviewers' box in the submission system. Please ensure you describe additional experiments that were carried out and include a detailed rebuttal of any

criticisms or requested revisions that you disagreed with. Please also ensure that your revised manuscript conforms to the journal style, which can be found in the Instructions for Authors on the journal homepage. If the data and code has been modified in the revision process please be sure to update the public versions of this too.

The due date for submitting the revised version of your article is 07 Apr 2022.

We look forward to receiving your revised manuscript soon.

Best wishes,

Hans Zauner
GigaScience
www.gigasciencejournal.com

Reviewer reports:
Reviewer #1: thank you for responding to the comments;

I have only a few very minor additional comments, which can be dealt with without going through another round of revision

1 : maybe explain b_i in your equation for a genotype likelihood; is b_i the base at site i ?

2: In the derivation of Eq 1, maybe relate 'sequence data D_j' more clearly to the genotypes G_j, i.e. explain the format of each D_j

Reviewer #2: The authors have addressed my concerns.

Close
