# Supplementary material for: Fast and accurate estimation of multidimensional site frequency spectra from low-coverage high-throughput sequencing data
Source: Gigascience. 2022 May 17;11:giac032. doi: 10.1093/gigascience/giac032 (PMC9112775; doi:10.1093/gigascience/giac032)
Supplement: giac032_Response_to_Reviewer_Comments_Revision_2 [file giac032_response_to_reviewer_comments_revision_2.pdf]

Author's Response To Reviewer Comments

Close

Dear Editor.

Below is a point to point list of the changes:

- Please include your new GigaDB dataset in the bibliography. The citation is:

[38] Mas-Sandoval A, Pope NS, Nielsen KN, Altinkaya I, Fumagalli M, Korneliussen TS. Supporting data for "Fast and accurate estimation of multidimensional site frequency spectra from low-coverage high-throughput sequencing data" GigaScience Database 2022. http://dx.doi.org/10.5524/100988

Done

- Please cite the GigaDB entry from the "availability" section (i.e. replace "XXX" with [38].

Done

- Also in the availability section, please add a biotoolsID identifier in addition to the RRID.

Done
- Please add the ORCIDs to the title page:
Alex Mas-Sandoval [0000-0002-1712-9404]; Nathaniel S Pope [0000-0001-8409-7812]; Knud Nor Nielsen [0000-0002-5777-1485]; Isin Altinkaya [0000-0002-6364-3332]; Matteo Fumagalli [0000-0002-4084-2953]; Thorfinn Sand Korneliussen [0000-0001-7576-5380];

Done

Close
