# Supplementary material for: Fast and accurate estimation of multidimensional site frequency spectra from low-coverage high-throughput sequencing data
Source: Gigascience. 2022 May 17;11:giac032. doi: 10.1093/gigascience/giac032 (PMC9112775; doi:10.1093/gigascience/giac032)
Supplement: giac032_Reviewer_1_Report_Original_Submission — Bjarki Eldon, PhD -- 12/3/2021 Reviewed [file giac032_reviewer_1_report_original_submission.pdf]

Reviewer Report

Title: Fast and accurate estimation of multidimensional site frequency spectra from low-coverage high-throughput sequencing data

Version: Original Submission **Date:** 12/3/2021

Reviewer name: Bjarki Eldon, PhD

Reviewer Comments to Author:

GIGA-D-21-00341
review report
The authors introduce a new method for estimating the site-frequency spectrum from
low-coverage high-throughput DNA sequence data. This is important since it saves on the cost of sequencing,
enabling researchers to direct resources towards obtaining more samples.
The work is well motivated and well written, and the difference in memory usage is impressive.
My main comment (see below) concern the presentation of the arguments for Eq 1; and on providing additional
background on genotype likelihoods. If you run into space restrictions for the main text, then I assume you could provide this in Supporting Information.
The comments aside, the methods are appropriate to the aims of the study, could be better described (see my comments),
necessary controls are included, and conclusions are adequately supported.
Comments:
0: I think for reviewing purposes it would have been better to format the manuscript into single-column mode
with line numbers; or maybe it is possible to include line numbers in two-column mode ?
1: An over-representation of rare alleles (e.g. singletons) can be an indicator of many things, not just population expansion; and repeated strong bottlenecks can be shown to generate gene genealogies looking
like multiple-merger genealogies, which generally predict a U-shaped SFS, i.e. an excess of low and high-frequency
variants
2: I think it would be good to rewrite the paragraph around Eq 1. The vector y needs to be better explained,
i.e. is y[i] is the likelihood of seeing i derived alleles in a haploid sample of size n ? Can I interpret a 'sample' as
one chromosome, for which you have x number of reads ? I am also confused about the notation : if y[i] is the ith coordinate
of y, where does y^{(1)} fit in? And \phi(i,j) in Eq 1 is a component of y, the jth component maybe ? You also need

to give some arguments how you obtain Eq 1.
3: Please include background on genotype likelihoods, and how they are defined and calculated. If I remember correctly there
is an equation for the likelihood in the original paper on genotype likelihoods. This equation should be included here and
explained. I am aware the current manuscript is submitted as a technical note, and seems directed towards those using
these methods on a daily basis, but there should still be room for additional background and clarifications.
4: by a 'genomic region' you are referring to a segment of a chromosome ?
5: Having the algorithms is nice, but I think it would be good if you explained in words how you generalized the EM-algorithm in
Ref 5
6: thank you for providing the command line for the program ms, but for those without detailed knowledge of the input for ms, maybe
provide a description of the input values you used ?
7: do you have references for the 'realistic' values of the recombination and mutation rates you used ?
8: the program ms has been around for a while, and used by many, but there is a new kid in town called msprime, which is not only
orders of magnitude more efficient than ms for common tasks, but is also thoroughly tested. So I was wondering if you would
be willing to redo your analysis in msprime, and so provide a msprime-based pipeline for your analysis. I think from now on people will
be using msprime, so for reproducibility I think this might be helpful.
9: are there estimates of the error rate in these new sequencing methods ? I was wondering if your 1% sequencing error rate was
somewhere near real values.
10: How did you simulate the reads of the simulated data?
11: the site-frequency spectrum (SFS), by definition, is 'multidimensional' since it is a vector ? Or, framed another way, if you are
estimating the joint SFS for two or more populations, where (X_i, X_j) would be the (i,j) coordinate and X_i the count of sites
carrying i derived alleles in population 1, and X_j similar for population 2, I would call it the 'joint' SFS
12: correct me if I am wrong, but don't you need at least 2x coverage to compute genotype likelihoods ?

Level of Interest

Please indicate how interesting you found the manuscript: Choose an item.

Quality of Written English

Please indicate the quality of language in the manuscript: Choose an item.

Declaration of Competing Interests

Please complete a declaration of competing interests, considering the following questions:

- Have you in the past five years received reimbursements, fees, funding, or salary from an organisation that may in any way gain or lose financially from the publication of this manuscript, either now or in the future?
- Do you hold any stocks or shares in an organisation that may in any way gain or lose financially from the publication of this manuscript, either now or in the future?
- Do you hold or are you currently applying for any patents relating to the content of the manuscript?
- Have you received reimbursements, fees, funding, or salary from an organization that holds or has applied for patents relating to the content of the manuscript?
- Do you have any other financial competing interests?
- Do you have any non-financial competing interests in relation to this paper?

If you can answer no to all of the above, write 'I declare that I have no competing interests' below. If your reply is yes to any, please give details below.

I declare that I have no competing interests

I agree to the open peer review policy of the journal. I understand that my name will be included on my report to the authors and, if the manuscript is accepted for publication, my named report including any attachments I upload will be posted on the website along with the authors' responses. I agree for my report to be made available under an Open Access Creative Commons CC-BY license (http://creativecommons.org/licenses/by/4.0/). I understand that any comments which I do not wish to be included in my named report can be included as confidential comments to the editors, which will not be published.

Choose an item.

To further support our reviewers, we have joined with Publons, where you can gain additional credit to further highlight your hard work (see: https://publons.com/journal/530/gigascience). On publication of this paper, your review will be automatically added to Publons, you can then choose whether or not to claim your Publons credit. I understand this statement.

Yes Choose an item.
