# Supplementary material for: Fast and accurate estimation of multidimensional site frequency spectra from low-coverage high-throughput sequencing data
Source: Gigascience. 2022 May 17;11:giac032. doi: 10.1093/gigascience/giac032 (PMC9112775; doi:10.1093/gigascience/giac032)
Supplement: giac032_Reviewer_2_Report_Original_Submission — Ryan Gutenkunst -- 12/3/2021 Reviewed [file giac032_reviewer_2_report_original_submission.pdf]

Reviewer Report

Title: Fast and accurate estimation of multidimensional site frequency spectra from low-coverage high-throughput sequencing data

Version: Original Submission **Date:** 12/3/2021

Reviewer name: Ryan Gutenkunst

Reviewer Comments to Author:

The authors present an implementation of a method for efficiently inferring the multidimensional site frequency spectrum from low-coverage genome data. The implementation is an update to ANGSD, a widely-used software package for manipulating genomic data.

Overall, the improved tool is likely to be of interest to those in the population genomics community, particularly because many applications will no longer require server-sized amounts of memory. The paper is generally clear, with exceptions noted below.

Major points:

1) The main text figures are absolutely illegible at printed size. They need to be carefully reworked with larger fonts and points.

2) I am confused by the benchmark in Table 1. The Methods section implies that the resulting spectrum is 1-pop ("assuming they are from a single population"), but the results seciton describes "estimating the multidimensional SFS". Which is it?

3) I am confused by the differences between Fig 2 and Fig S6. In principle, the 2-population spectra obtained from marginalizing the 3-pop spectrum should be identical to the 2-pop spectra obtained directly from the data. Can the authors comment on the differences between these approaches when their algorithm is applied?

Minor points:

1) Results, Computational Performance: The phrase "no longer exhibits proportional increase of computational requirements with sample size" seems incorrect. The data show that for both the orginal and banded methods, the time increases with sample size. Do the authors mean "memory requirements"?

2) Results, Estimation of frequency spectra: The claim is made that "mean estimates... do not differ". This is not quite true for the multipopulation spectra, where there is a systematic bias upward for low-coverage estimates of sfs cells which contain little to no data. This is acknowledged later in the section, but I would ask the authors to be more explicit. (I suspect such biases are unlikely to affect downstream analyses substantially, since they affect only a small fraction of the total weight in the SFS.)

3) Figure 1 caption: log_{10} instead of "log_10 scale".

Level of Interest

Please indicate how interesting you found the manuscript: Choose an item.

Quality of Written English

Please indicate the quality of language in the manuscript: Choose an item.

Declaration of Competing Interests

Please complete a declaration of competing interests, considering the following questions:

- Have you in the past five years received reimbursements, fees, funding, or salary from an organisation that may in any way gain or lose financially from the publication of this manuscript, either now or in the future?
- Do you hold any stocks or shares in an organisation that may in any way gain or lose financially from the publication of this manuscript, either now or in the future?
- Do you hold or are you currently applying for any patents relating to the content of the manuscript?
- Have you received reimbursements, fees, funding, or salary from an organization that holds or has applied for patents relating to the content of the manuscript?
- Do you have any other financial competing interests?
- Do you have any non-financial competing interests in relation to this paper?

If you can answer no to all of the above, write 'I declare that I have no competing interests' below. If your reply is yes to any, please give details below.

I declare that I have no competing interests

I agree to the open peer review policy of the journal. I understand that my name will be included on my report to the authors and, if the manuscript is accepted for publication, my named report including any attachments I upload will be posted on the website along with the authors' responses. I agree for my report to be made available under an Open Access Creative Commons CC-BY license (http://creativecommons.org/licenses/by/4.0/). I understand that any comments which I do not wish to be included in my named report can be included as confidential comments to the editors, which will not be published.

Choose an item.

To further support our reviewers, we have joined with Publons, where you can gain additional credit to further highlight your hard work (see: https://publons.com/journal/530/gigascience). On publication of this paper, your review will be automatically added to Publons, you can then choose whether or not to claim your Publons credit. I understand this statement.

Yes Choose an item.
